# A subset of diffuse-type gastric cancer is susceptible to mTOR inhibitors and checkpoint inhibitors

**DOI:** 10.1186/s13046-019-1121-3

**Published:** 2019-03-12

**Authors:** Hiroshi Fukamachi, Seon-Kyu Kim, Jiwon Koh, Hye Seung Lee, Yasushi Sasaki, Kentaro Yamashita, Taketo Nishikawaji, Shu Shimada, Yoshimitsu Akiyama, Sun-ju Byeon, Dong-Hyuck Bae, Keisuke Okuno, Masatoshi Nakagawa, Toshiro Tanioka, Mikito Inokuchi, Hiroshi Kawachi, Kiichiro Tsuchiya, Kazuyuki Kojima, Takashi Tokino, Yoshinobu Eishi, Yong Sung Kim, Woo Ho Kim, Yasuhito Yuasa, Shinji Tanaka

**Affiliations:** 1 Department of Molecular Oncology, Graduate School of Medical and Dental Sciences, Tokyo Medical and Dental University, Tokyo, Japan; 2 Personalized Genomic Medicine Research Center, Korea Research Institute of Bioscience and Biotechnology, Daejeon, Korea; 3 Department of Pathology, Seoul National University Bundang Hospital, Gyeonggi-do, Korea; 4 Medical Genome Sciences, Research Institute for Frontier Medicine, Sapporo Medical University School of Medicine, Sapporo, Japan; 5 Department of Gastroenterology and Hepatology, Sapporo Medical University School of Medicine, Sapporo, Japan; 6 Department of Pathology, Seoul National University College of Medicine, Seoul, Korea; 7 Genome Editing Research Center, Korea Research Institute of Bioscience & Biotechnology, Daejeon, Korea; 8 Department of Gastrointestinal Surgery, Graduate School of Medical and Dental Sciences, Tokyo Medical and Dental University, Tokyo, Japan; 9 Department of Human Pathology, Graduate School of Medical and Dental Sciences, Tokyo Medical and Dental University, Tokyo, Japan; 10 Department of Gastroenterology and Hepatology, Tokyo Medical and Dental University, Tokyo, Japan; 11 Center of Minimally Invasive Surgery, Graduate School of Medical and Dental Sciences, Tokyo Medical and Dental University, Tokyo, Japan; 12 Present Address: Division of Cancer Biology and Therapeutics, Miyagi Cancer Center Research Institute, Miyagi, 981-1293 Japan; 13 Present Address: Department of Pathology, The Cancer Institute Hospital of the Japanese Foundation for Cancer Research, Tokyo, 135-8550 Japan

**Keywords:** Diffuse-type gastric cancer, mTOR inhibitor, Temsirolimus, PIK3CA, Microsatellite unstable, Immune checkpoint inhibitor, PD-L1, Patient-derived xenograft, Primary culture

## Abstract

**Background:**

Mechanistic target of rapamycin (mTOR) pathway is essential for the growth of gastric cancer (GC), but mTOR inhibitor everolimus was not effective for the treatment of GCs. The Cancer Genome Atlas (TCGA) researchers reported that most diffuse-type GCs were genomically stable (GS). Pathological analysis suggested that some diffuse-type GCs developed from intestinal-type GCs.

**Methods:**

We established patient-derived xenograft (PDX) lines from diffuse-type GCs, and searched for drugs that suppressed their growth. Diffuse-type GCs were classified into subtypes by their gene expression profiles.

**Results:**

mTOR inhibitor temsirolimus strongly suppressed the growth of PDX-derived diffuse-type GC-initiating cells, which was regulated via Wnt-mTOR axis. These cells were microsatellite unstable (MSI) or chromosomally unstable (CIN), inconsistent with TCGA report. Diffuse-type GCs in TCGA cohort could be classified into two clusters, and GS subtype was major in cluster I while CIN and MSI subtypes were predominant in cluster II where PDX-derived diffuse-type GC cells were included. We estimated that about 9 and 55% of the diffuse-type GCs in cluster II were responders to mTOR inhibitors and checkpoint inhibitors, respectively, by identifying *PIK3CA* mutations and MSI condition in TCGA cohort. These ratios were far greater than those of diffuse-type GCs in cluster I or intestinal-type GCs. Further analysis suggested that diffuse-type GCs in cluster II developed from intestinal-type GCs while those in cluster I from normal gastric epithelial cells.

**Conclusion:**

mTOR inhibitors and checkpoint inhibitors might be useful for the treatment of a subset of diffuse-type GCs which may develop from intestinal-type GCs.

**Electronic supplementary material:**

The online version of this article (10.1186/s13046-019-1121-3) contains supplementary material, which is available to authorized users.

## Background

Gastric cancer (GC) is the fifth most common cancer and the third leading cause of cancer-related deaths worldwide with nearly one million new cases every year [1]. GCs are classified into intestinal and diffuse types [2]. Intestinal-type GCs are composed of glandular solid cells, while diffuse-type GCs consist of poorly cohesive cells that tend to infiltrate the gastric wall. Intestinal-type GCs are more commonly diagnosed in aged patients, have a male preponderance, and are strongly associated with gastric mucosal atrophy and intestinal metaplasia, both of which are induced by chronic *Helicobacter pylori* infection. In contrast, diffuse-type GCs are diagnosed in younger patients, and occur in both sexes [3], but their mechanism of development has not yet been fully understood. Ikeda et al. found that the ratio of diffuse-type GCs was increased in advanced GCs compared with that in early ones, and suggested that, in some GCs, the predominant histologic type may be altered from intestinal- to diffuse-type with progression of the tumor [4]. Arai et al. reported that microsatellite unstable (MSI) GCs were significantly related with older age, female gender, and predominant papillary adenocarcinoma and solid-type, poorly differentiated adenocarcinoma, and they suggested that GC with MSI may originate from differentiated-type carcinomas [5]. However, further analyses do not appear to have been reported. Histological heterogeneity is often found in GC tissues, and mixed-type GCs composed of intestinal- and diffuse-type tissues are found in about 22–25% of cases, and they exhibit worse prognosis than non-mixed-type GCs [6, 7]. However, it is not clear how the development of mixed-type GCs is related to that of other GC types.

Diffuse-type GC cells often exhibit more aggressive characteristics than intestinal-type cells, such as rapid infiltrative growth accompanied by massive stromal fibrosis. These diffuse-type GCs are called scirrhous-type GCs, and are associated with frequent metastasis to lymph nodes and the peritoneum, which contributes to their poor prognosis [8]. Many GC patients with advanced disease eventually experience relapse, even after curative surgical resection. Following progression on first-line platinum- and fluoropyrimidine-based chemotherapy, prognosis for advanced diffuse-type GC patients is extremely poor. The poor outcomes suggest a need for molecularly targeted agents that may confer a better survival benefit, but only a few targeted therapies have been developed for such patients [9].

Cancer stem cells, or cancer-initiating cells, are cells that possess the capacity to self-renew and generate heterogeneous neoplastic cell lineages that can reconstitute the cancer [10]. Recent research has shown that the stem cell condition is plastic and reversible in response to the microenvironment. Thus, eradication of cancer-initiating cells by targeted therapeutics has not been as easy as was initially hoped [11]. Clinical trials targeting cancer-initiating cells are now being conducted to evaluate the efficacy of this approach in a variety of cancers [12–14]. However, further efforts to explore new and more effective drugs remain warranted. With a greater understanding of the growth regulatory mechanisms in GC-initiating cells, we may improve patient selection for biological therapy, and thus further enhance outcomes for GC patients.

We previously found that CD49f, a biomarker commonly found in various stem cells including some cancer stem cells [15], was a marker of GC-initiating cells in both intestinal- and diffuse-type GCs while CD44^+^/CD133^+^ cells were not always tumorigenic [16]. We also succeeded in cultivating tumor-initiating cells from patient-derived xenograft (PDX) lines established using intestinal-type GCs. These intestinal-type GC-initiating cells strongly expressed CD49f, and grew to form spheres in primary culture [16]. However, diffuse-type GC-initiating cells have not yet been fully characterized. In the present study, we therefore identified GC-initiating cells in PDX lines established from diffuse-type GCs, analyzed their characteristics, and searched for drugs that suppress their growth. We found that some molecularly targeted drugs might be useful for the treatment of a subset of diffuse-type GCs.

## Methods

### Establishment of PDX lines and characterization of PDX cells by FACS

Methods of establishment of PDX lines and their characterization with fluorescence activated cell sorting (FACS) have been described in a previous paper [16]. HGC-18 and -20 lines were used at passage number 1–4, but HGC-3 at passage number 5–9, because HGC-3 tissues at early passages were exhausted by 2015. Features of PDX lines established from intestinal-type GCs have been described in the previous paper [16]. To detect programmed death-ligand 1 (PD-L1) by FACS, PE-labelled anti-human CD274 (B7-H1, PD-L1) antibody, and PE-labelled mouse IgG2b, κ Isotype control antibody (both from BioLegend) were used.

### Primary culture of PDX cells

PDX tissues were dissociated into single cells as previously described [16], with some modifications. In the previous study, we used trypsin-EDTA to dissociate intestinal-type GC PDX tissues, but in the present study, we first treated diffuse-type GC PDX tissues with 0.1% collagenase (Wako) for 30 min at 37 °C, followed by 0.05% trypsin-0.53 mM EDTA with 0.01% DNase for 30 min at 37 °C, because diffuse-type GC PDXs contain greater amount of collagen fibers in them. In the previous study, we used Renal Epithelial Basal Medium (REBM; Lonza) supplemented with B27 (Invitrogen), epidermal growth factor (EGF), gastrin, and Y-27632 (all from Wako) to cultivate intestinal-type GC-initiating cells [16]. In the first part of our experiments where we examined effect of anti-tumor drugs on the growth of diffuse-type GC-initiating cells, we used this REBM-based medium to compare effect of the drugs on the growth of intestinal-, diffuse-, and mixed-type GC-initiating cells in culture.

Recently, Bartfeld et al. reported that human non-tumor gastric epithelial cells grew to form organoids in advanced Dulbecco’s modified Eagle medium /F12 (ADF; Invitrogen)-based medium supplemented with growth factors [17]. We confirmed their results (Additional file 1: Figure S1), and found that PDX cells grew much faster in the ADF-based medium than in the REBM-based medium (Additional file 1: Figure S2). We thus used the ADF-based medium supplemented with 10 mM HEPES, GlutaMAX (all Invitrogen), B27, Primocin (Invivogen), 1 mM N-acetylcysteine (Sigma-Aldrich), 30 ng/mL EGF, 1 nM gastrin, and 10 μM Y-27632 as basal medium in the second part of our experiments where we examined effect of molecularly targeted drugs on the growth of diffuse- and intestinal-type GC-initiating cells. For the cultivation of PDX cells, the basal medium was supplemented with 25% R-spondin1-conditioned medium (CM) and 25% Wnt3a-CM, which were prepared according to a protocol by Broutier et al. [18].

The expression of PD-L1 on cultured cells was assessed by culturing 5 × 10^5^ PDX cells in rat tail collagen-treated 12-well plates for 14 days. To examine effects of chemicals on the expression of PD-L1, drugs were added on day 9 in culture so that cells were treated with them for 5 days. Easily-detached cells were harvested by washing the cells with a Pasteur pipette, and then attaching cells were dissociated by trypsin-EDTA treatment. In some plates, easily-detached and attached cells were combined and they were called “whole cells”. This method was used to obtain easily-detached, attached, and whole cell fractions from HGC-3, HGC-20, MKN45, and NUGC-4 cells, and their cell surface antigen was analyzed by FACS with the anti-CD274 antibody described above.

### Tumorigenicity assay and histological analysis

The method has been described in the previous paper [16]. Briefly, cultured cells were dissociated into single cells by trypsin-EDTA treatment, and graded numbers of cells were suspended in 50 mL CMF-PBS. Cell suspension was mixed with an equal volume of Matrigel (Corning), and subcutaneously injected into NOD-SCID mice (Charles River Japan, Yokohama, Japan) on the dorsal side of each flank. To minimize experimental variability due to individual differences in recipient mice, cell populations that were to be compared were injected on opposite flanks of the same animal. The injected mice were maintained for up to 6 months, and killed when tumor diameters reached 10 mm. Tumor tissues were fixed with 95% ethyl alcohol, embedded in paraffin and sectioned at 5 µM. Sections were deparaffinized and hydrated using xylene and ethyl alcohol, and stained with hematoxylin and eosin, and Alcian blue (pH2.5)-PAS-hematoxylin.

### Culture of established cell lines

MKN45, MKN74, and NUGC-4 human GC cell lines were obtained from JCRB Cell Bank (Osaka, Japan), and their quality was assessed by their short tandem repeat profiles by BEX Co. (Tokyo, Japan). They were maintained in Dulbecco’s modified Eagle medium (DMEM; Wako) (MKN45 and MKN74 cells) or RPMI1640 medium (Wako) (NUGC-4 cells) supplemented with 10% heat-inactivated fetal bovine serum (FBS; Hyclone), but they were cultured in serum-free media used for the culture of PDX cells when effect of drugs on their growth was examined.

### Organoid culture of non-tumor gastric epithelial cells

Non-tumor gastric epithelial cells were cultured by using a method developed by Hans Clevers’ laboratory [17]. Non-tumor gastric tissues were washed with cold Hanks’ buffered saline solution, and fats and connective tissues were removed. The tissues were cut into pieces of approximately 1 × 3 mm, thoroughly washed with cold DMEM supplemented with 10% FBS and penicillin-streptomycin solution (Wako), and stored in the medium overnight at 4 °C to remove contaminated microorganisms. Then, they were thoroughly washed with CMF-PBS and incubated in chelating solution (distilled water with 5.6 mM Na_2_HPO_4_, 8.0 mM KH_2_PO_4_, 96.2 mM NaCl, 1.6 mM KCl, 43.4 mM sucrose, 54.9 mM D-sorbitol, 0.5 mM DL-dithiothreitol, 2 mM EDTA) for 30 min at room temperature. Epithelial and stromal tissues were separated with the aid of forceps under a dissecting microscope. Isolated glands were re-suspended in DMEM supplemented with 10% FBS, dissociated into small fragments by pipetting, and mixed with Matrigel, and seeded on dishes pre-coated with Matrigel. After the gel was polymerized, the tissues with gel was overlaid with ADF medium supplemented with 10 mM HEPES, GlutaMAX. B27, Primocin, 200 ng/ml fibroblast growth factor-10, 25 ng/ml noggin (all from Peprotech), 2 μM A-83-01, 10 mM nicotinamide (all from Sigma-Aldrich), 1 mM N-acetylcysteine, 30 ng/ml EGF, 1 nM gastrin, 10 μM Y-27632, 25% R-spondin1-CM, and 25% Wnt3a-CM. Cultures were kept at 37 °C, and at 5% CO_2_ in a humidified incubator.

### Effect of drugs on the growth of diffuse- and mixed-type GC-initiating cells in primary culture

The method has been described in the previous paper [16]. Briefly, HGC-3, − 18 and − 20 PDXs were dissociated into single cells by collagenase and Trypsin-EDTA treatment as described above, and seeded in rat tail collagen-treated 24-well plates. Effect of anti-tumor drugs on the growth of PDX cells was examined by seeding HGC-3, -18, and -20 cells at 50,000 cells per well, and by seeding MKN45 and NUGC-4 cells at 1000 cells per well, in 0 .5mL REBM-based medium on day 0, and by adding 0 .5mL media containing × 2 concentrated 5-fluorouracil (5-FU), doxorubicin, or doxifluridine (all from Wako) on day 1. 5-FU and doxifluridine were dissolved in dimethyl sulfoxide (DMSO), while doxorubicin in water. DMSO (final concentration = 0.1%) was added to each well when cells were treated with 5-FU or doxifluridine. Cell numbers were determined after 2 weeks by using (4,5-dimethylthiazol-2-yl)-2,5-diphenyltetrazolium bromide (MTT; Dojindo) as substrate as described previously [16].

Effect of molecularly targeted drugs and signaling inhibitors on the growth of HGC-3 and -20 cells was examined by seeding 10^5^ cells in 0 .5mL ADF-based medium on day 0, and by adding 0 .5mL medium containing 2x concentrated temsirolimus (Abcam), everolimus (Adooq), PP242 (Adooq), CHIR99021 (Wako), or FH535 (Sigma-Aldrich) on day 1. DMSO was used as solvent for the drugs and was used as a control. Cell number was determined by MTT assay on day 5.

### RNA extraction, RNA sequencing, and data processing

Total RNAs were isolated from kelp-like structures (KLSs) formed by HGC-3, -18, and -20 cells by using RNeasy Mini Kit (Qiagen) according to the manufacturer’s protocol. The sequencing library was prepared and RNA-seq data were processed as has been reported previously [19]. Quantification of each gene was performed using HTseq-count utility involved in the HTSeq framework (ver. 0.9.1). To handle expression levels of the genes in the RNA-seq data, counts per million mapped reads of each sample were calculated. The counts per million data were normalized by the quantile method, log2-transformed, and median-centered across genes and samples. Expression differences in genes in the RNA-seq data were considered significant if the fold difference in expression between two sample groups was ≥3.

To compare the molecular characteristics between PDXs and other GC samples, a hierarchical clustering analysis was carried out. For cluster analysis, we obtained public data sets of primary diffuse-type GCs from TCGA (https://cancergenome.nih.gov/) and those of GC cell lines from Cancer Cell Line Encyclopedia (CCLE; https://portals.broadinstitute.org/ccle). Before clustering, the expression levels of each gene in each data set were standardized independently, to a mean of zero and a standard deviation of 1. Then, the data sets of TCGA, CCLE, and PDX samples were pooled into a whole gene expression data matrix, which were normalized by quantile normalization method. A hierarchical clustering algorithm, using the centered correlation coefficient as the measure of similarity and centroid linkage clustering, was applied.

Gene set enrichment analysis was carried out to identify the most significant gene sets associated with the Kyoto Encyclopedia of Genes and Genome pathway database, in which the significance of over-represented gene sets was estimated by Fisher’s exact test. Gene set enrichment analysis was performed using the DAVID Bioinformatics Resources (ver. 6.8).

To verify molecular subtypes of GC cells, we generated four prediction models for Epstein-Barr virus (EBV), MSI, genomically stable (GS), or chromosomally unstable (CIN) subtypes using data from TCGA cohort according to the previous study [20]. Briefly, to select EBV, MSI, GS, or CIN subtype specific genes, multiple two-class t-tests were performed for all possible combination of the four subtypes (*P* < 0.001 by two sample t-test). Genes having statistical significance in expression in all three possible comparisons were selected as subtype-specific genes, revealing 718 genes for the EBV subtype, 499 genes for the MSI subtype, 1965 genes for the GS subtype, and 241 genes for the CIN subtype. The top 200 genes in each subtype were further selected for development of the prediction models. For construction of four subtype prediction models, we applied a machine learning-based prediction algorithm using the Bayesian compound covariate prediction method. After construction of the models, RNA-seq quantification data of three diffuse- and mixed-type GC-initiating cells were applied into the models and their molecular subtypes were classified. Subtype prediction procedure was performed based on the BRB-array tools (version 4.5.0).

### Screening of drugs that suppress the growth of diffuse-type GC-initiating cells and identification of signaling pathway that regulates their growth

The SCADS inhibitor kit I and II, kinase inhibitor kit, and molecularly targeted drug kit (total number of drugs was 386) were kindly provided by the Screening Committee of Anticancer Drugs (Japan). We regarded drugs “positive” when the growth of HGC-20 cells was suppressed to less than 50% of controls by the drug at 1 μM. Experiments were repeated at least twice, and dose-response relationships were confirmed with positive drugs. We then examined whether the drugs affected the growth of PDX tissues in vivo. HGC-3 and -20 PDX tissues were dissociated into single cells, and tumors were produced on the backs of nude mice (KSN/Slc, Japan SCL, Japan) by subcutaneously injecting 2 × 10^5^ cells mixed with Matrigel. After 7 days, tumor-bearing animals were randomized, and temsirolimus, bortezomib (Adooq), and YM155 (Focus) were intraperitoneally administrated at 1–10 mg/kg body weight to the animals twice weekly. After 6–7 weeks when the tumor was above 1 cm in diameter, animals were sacrificed and tumors were recovered to examine their weight. We then compared effect of mechanistic target of rapamycin (mTOR) inhibitor analogues including temsirolimus, everolimus, and PP242 on the growth of HGC-3 and -20 cells in vitro and in vivo, using the method described above. We also used CHIR99021 and FH535 to identify the signal transduction system in the PDX cells in culture.

### Tissue array analyses

A total of 610 and 372 primary GCs from formalin-fixed paraffin-embedded tissue microarray samples in Seoul National University Hospital and Seoul National University Bundang Hospital were used for immunohistochemical analyses of mTOR and PD-L1 expressions, respectively, in GCs. Expression of phosphorylated-mTOR (p-mTOR) was examined by using anti-p-mTOR rabbit monoclonal antibody (clone 49F9) from Cell Signaling Technology, and Ventana BenchMark XT automatic immunostainer systems. We classified tissues into high- and low-expressing by using a score system where we called a tissue “high” when more than 40% of the tumor cells were positively stained, and “low” when less than 40% of the tumor cells were stained. Kaplan-Meier relationship was examined, and univariate and multivariate analyses were performed by using GC tissues obtained.

Expression of PD-L1 was examined by using anti-PD-L1 antibody (clone E1L3N, rabbit monoclonal; Cell Signaling Technology) and Ventana BenchMark XT automatic immunostainer systems. Features of patients used for the analysis are described in a previous paper [21]. Membrane staining of PD-L1 on more than 5% of tumor cells was interpreted as positive. mTOR was immunohistochemically detected as described above, and membranous and cytoplasmic staining of mTOR was evaluated using intensity score: 0 (no tumor cell staining), 1 (weak), 2 (moderate), and 3 (strong), as described in a previous paper [22]. By combining results obtained with staining for mTOR and PD-L1, we examined the correlation between mTOR and PD-L1 expressions in diffuse-type GCs, by using Chi-square test.

### Mutation analysis

Mutation profiles of primary GCs in TCGA are obtained from The cBioPortal for Cancer Genomics (http://www.cbioportal.org/). Mutations and copy number variations (gain and loss) of PDX cells in 409 cancer-related genes were analyzed using the Ion Comprehensive Cancer Panel and Ion Proton sequencer (Thermo Fisher Scientific). A non-tumor gastric tumor mucosa (5Y26) was used as a control. Sequencing and data analysis were performed as described [23].

### Western blot analysis

Total protein was extracted using RIPA buffer and was then electrophoresed on SDS-PAGE. Western blotting was conducted with mouse monoclonal anti-E-cadherin (1:500; BD Bioscience), anti-MLH1 (1:500; Pharmingen) and anti-MSH2 (1:500; Merck) antibodies, as has been reported [24].

### Methylation-specific polymerase chain reaction analysis

Methylation-specific polymerase chain analysis was performed as described previously [24]. The primer sequences for methylation analyses are shown in a previous paper [25].

### MSI analysis and CIN analysis

MSI analysis was performed according to Yamashita et al. [26], by using BAT25, BAT26, and BAT40 as microsatellite markers. High frequency MSI (MSI-H) was defined as MSI in > 30% of the markers examined. Chromosome number was analyzed according to Fleicher [27].

### Statistical analyses

Results were statistically analyzed using the non-parametric Mann-Whitney’s U test or Student’s t-test. For the analysis of data in tables, Chi-square test was used. A *P* values of < 0.05 were considered statistically significant. Patient survival was calculated from the date of surgery until death or the date of last follow-up. Survival was evaluated by the Kaplan-Meier method, and uni- and multi-variate survival analyses were performed using the Cox proportional hazard model.

## Results

### Features of diffuse-type GC PDXs and diffuse-type GC-initiating cells in primary culture

**Fig. 1:**
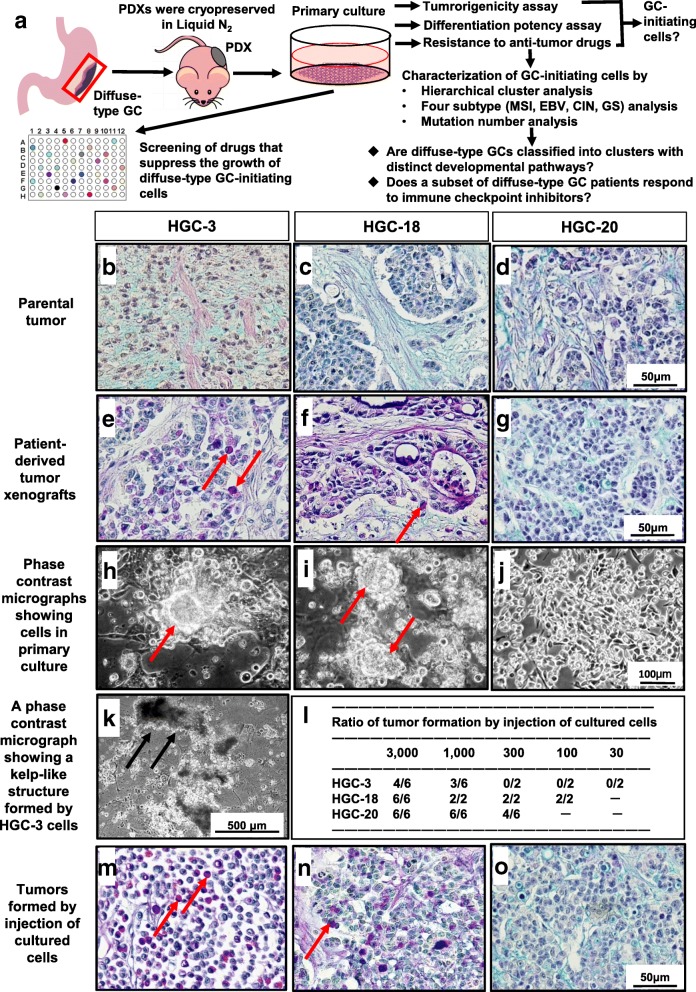
Schematic diagram of the procedures and features of PDX lines in vivo and in vitro. **a** Schematic diagram of the experimental procedures. Tumorigenicity and differentiation potency of PDX cells grown in primary culture were examined to determine whether they were GC-initiating cells. They were further characterized by gene expression profiles and mutation number analyses. By identifying drugs that suppress their growth, new targeted therapy for diffuse-type GCs was proposed. **b**-**o** Light micrographs showing histological features of (**b**-**d**) parental tumors, (**e**-**g**) PDXs, and (**m**-**o**) tumors formed by injection of cultured cells. Signet ring cells (red arrows in **e**, **f**, **m**, **n**) were found in HGC-3 and -18 PDXs and tumors formed by injection of cultured HGC-3 and -18 cells. Stained with Alcian Blue (pH2.5)-PAS-hematoxylin. **h**-**k** Phase contrast micrographs of **h**, **k** HGC-3, **i** HGC-18, and **j** HGC-20 cells in primary culture. Spheres (red arrows) were found in culture of HGC-18 and -3 cells. When HGC-3 cells were cultured for weeks, kelp-like structures were found (**k**, black arrows) (also see Additional file 3: Movie S1). **l** Ratio of tumor formation by injection of cultured cells into NOD-SCID mice. Clinicopathological features of diffuse-type GC patients whose cancers were used for the establishment of PDX lines are described in Additional file 2: Table S1

Schematic diagram of experimental procedures is illustrated in Fig. 1a. We established three PDX lines (HGC-3, -18, and -20) from diffuse-type GCs. Clinicopathological features of diffuse-type GC patients whose cancers were used for the establishment of PDX lines are described in Additional file 2: Table S1. We confirmed that PDX lines retained histological features of the original tumors (Fig. 1b-g). Signet ring cells were found in all PDXs. In HGC-3 and -20 PDX lines, no glandular components were found, while in HGC-18 PDX line, both diffuse and glandular components always co-existed, indicating that it is a mixed-type GC (Fig. 1e-g). The HGC-18 PDX line may be useful to examine how mixed-type GCs are formed.

Cancer-initiating cells have been reported to form spheres in serum-free conditions, and we confirmed this in our previous study with intestinal-type GC-initiating cells [16]. In the present study, we found sphere formation of HGC-3 and -18 cells in culture, but only rarely in HGC-20 cells (Fig. 1h-j), indicating that sphere formation may not be a feature of diffuse-type GC-initiating cells. When these PDX cells were maintained for weeks in culture, some cells formed KLSs (Fig. 1k, Additional files 3, 4, 5: Movies S1-S3). Similar KLSs were also found in culture of MKN45 and NUGC-4 cells established from diffuse-type GCs (Additional files 6, 7: Movies S4, S5). Thus, KLSs may be a feature of diffuse-type GC cells in vitro. In the present study, we used KLS cells for the RNA sequencing analyses below.


**Additional file 3: Movie S1.** Kelp-like structures found in culture of HGC-3 cells. Please refer to Fig. 1k for scale bar. (MP4 673 kb)



**Additional file 4: Movie S2.** Kelp-like structures found in culture of HGC-18 cells. Please refer to Fig. 1k for scale bar. (MP4 587 kb)



**Additional file 5: Movie S3.** Kelp-like structures found in culture of HGC-20 cells. Please refer to Fig. 1k for scale bar. (MP4 1121 kb)



**Additional file 6: Movie S4.** Kelp-like structures found in culture of MKN45 cells. Please refer to Fig. 1k for scale bar. (MP4 1130 kb)



**Additional file 7: Movie S5.** Kelp-like structures found in culture of NUGC-4 cells. Please refer to Fig. 1k for scale bar. (MP4 1057 kb)


The tumorigenicity of cultured PDX cells was examined by subcutaneously injecting them into NOD-SCID mice. We found them to be highly tumorigenic, since 300–3000 cells were enough to induce tumor formation in the mice (Fig. 1l). Cultured PDX cells strongly expressed *BMI1, CD44, EpCAM,* and *SOX9* (Additional file 1: Figure S3), and HGC-3, -18, and -20 cells formed diffuse-, mixed-, and scirrhous-type tumors, respectively, in mice (Fig. 1m-o). We thus concluded that PDX cells in primary culture are GC-initiating cells.

We previously reported that intestinal-type GC-initiating cells expressed CD49f [16]. We examined whether diffuse- and mixed-type GC-initiating cells also expressed it. We found that about 5–25% of dissociated PDX cells strongly expressed it, and that their ratio increased to 10–30% in primary culture (Additional file 1: Figure S4). For intestinal-type cells, the ratio increased 5 to 10 times in culture, consistent with the idea that only CD49f^high^ cells could grow in culture, but for diffuse- and mixed-type cells, the increase was less, suggesting that CD49f may play a minor role in the growth of diffuse- and mixed-type GC-initiating cells.

### Diffuse- and mixed-type GC-initiating cells are more resistant to anti-tumor drugs than established cell lines and intestinal-type cells in culture

In our previous study, we found that some intestinal-type GC-initiating cells were more resistant to anti-tumor drugs than MKN45 and MKN74 cells, while others were equally as sensitive [16]. In the present study, HGC-3, -18 and -20 cells in primary culture were far more resistant to anti-tumor drugs than MKN45 or NUGC-4 cells (Additional file 1: Figure S5), suggesting that diffuse- and mixed-type GC-initiating cells are more resistant to the drugs than intestinal-type cells. This may be related to the fact that diffuse- and mixed-type GCs have a worse prognosis than intestinal-type GCs [6, 7].

### Diffuse-type GCs are classified into two clusters by gene expression profiles

**Fig. 2:**
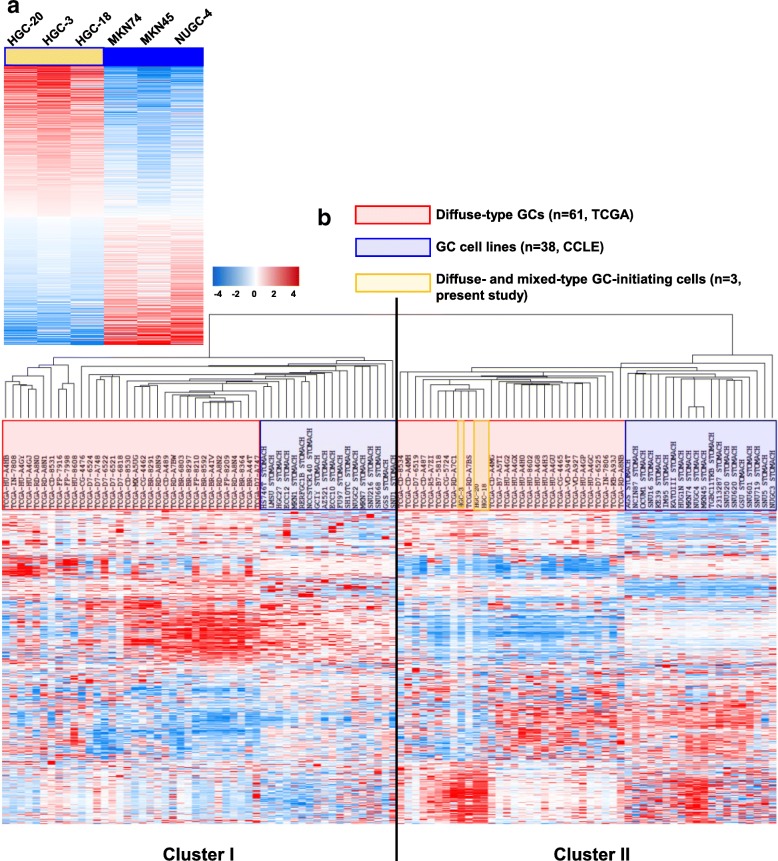
Diffuse- and mixed-type GCs are classified into two clusters by gene expression profiles. **a** A heat map of 1048 genes which were differentially expressed in HGC-20, -3, and -18 GC-initiating cells compared with established cell lines (MKN74, MKN45, and NUGC-4 cells), listed in Additional file 2: Table S2. **b** A cluster analysis on the expression of these 1048 genes using primary diffuse-type GCs (*n* = 61 from TCGA), GC cell lines (*n* = 38 from CCLE), and diffuse- and mixed-type GC-initiating cells (*n* = 3, present study)

To explore why diffuse- and mixed-type GC-initiating cells were more resistant to anti-tumor drugs than established GC cell lines, we compared gene expression profiles of HGC-3, -18, and -20 cells and established cell lines (MKN74, MKN45, and NUGC-4 cells) (Fig. 2a). We identified 1048 genes that were differentially expressed in these two groups (*P* < 0.05 by two sample t-test) (Additional file 2: Table S2). These genes may be related to the resistance to anti-tumor drugs, because several drug resistance-related genes such as solute carrier family genes were included in the list.

Using this gene list, hierarchical cluster analysis was performed on primary diffuse-type GCs from TCGA (*n* = 61) [28], on GC cell lines from CCLE (*n* = 38) [29], and on PDX cells in primary culture (*n* = 3, present study). We found that samples could be classified into two clusters (Fig. 2b), each of which consisted of primary diffuse-type GCs and established GC cell lines. Primary diffuse-type GCs and established GC cell lines were well separated, and HGC-3, -18, and -20 cells in primary culture were included in cluster II together with primary diffuse-type GCs, indicating that they retained features of primary GCs. In the following, diffuse-type GCs in clusters I and II are referred to as diffuse-type C-I and C-II GCs, respectively.

### Identification of temsirolimus as a potent growth suppressor of diffuse-type GC-initiating cells

Previously, established cell lines such as NCI-60 panel were used for drug screening, but in vivo activity in a specific tumor model did not closely correlate with activity in the same human cancer patients [30]. Gillet et al. found no correlation between clinical samples and established cancer cell lines on their multidrug resistance transcriptome, possibly because survival genes were up-regulated across all cultured cancer cell lines evaluated, and all cell lines bore more resemblance to each other, regardless of the tissue of origin, than to the clinical samples that they were supposed to model [31]. Thus, it is widely recognized that new primary tumor models are necessary for drug development.

Tumor organoid culture systems have recently been extensively developed, and they are now used for drug screening [32–34], and for prediction of responses to chemotherapy [35]. Primary GC organoid biobanks have recently been reported [36, 37]. Nanki et al. reported that diffuse-type GCs formed amorphous solid configurations with a loss of apico-basal polarity [36] while Yan et al. reported diffuse-type GCs grew as isolated cells or in loosely cohesive to solid cell clusters without glandular lumen formation [37]. The diffuse-type GC-initiating cells in the present study did not form organoids (Additional file 1: Figure S6) when cultured in a condition where non-tumor gastric epithelial cells formed organoids (Additional file 1: Figure S1), indicating that features of diffuse-type GC cells differed between laboratories. This may be caused by the different methods used. To eliminate overgrowing normal organoids, Nanki et al. selected *TP53* mutant, RHO-dysregulated, TGF-β-insensitive, and receptor tyrosine kinase-activated GC cells [36], and Yan et al. used Nutlin3a to enrich *TP53* mutants [37] to establish GC organoid cultures, but we just cultured cells without selection because normal gastric epithelial cells were not included in PDX. In the present study, HGC-20 cells in primary culture were used to identify drugs that suppress the growth of diffuse-type GC-initiating cells because (i) we confirmed that the cells retained features of primary GCs (Fig. 2b), (ii) they formed scirrhous-type tumors in immuno-deficient mice (Fig. 1o), (iii) they grew rapidly in culture, and (iv) PDXs at early passages were available.

By second-round screenings with 364 chemicals, nine, three, and three inhibitors were identified from kits I, III, and IV, respectively, which reproducibly suppressed cell growth (Additional file 1: Figure S7). The three inhibitors from kit IV were bortezomib, temsirolimus, and YM155. Temsirolimus and bortezomib are used for the treatment of patients with renal cell carcinoma [38], and multiple myeloma and mantle cell lymphoma [39], respectively, but they are not used for GC patients. Considering that it is essential to identify new molecularly targeted drugs for their treatment, effects of these molecularly targeted drugs on the growth of diffuse-type GC-initiating cells were further investigated.

**Fig. 3:**
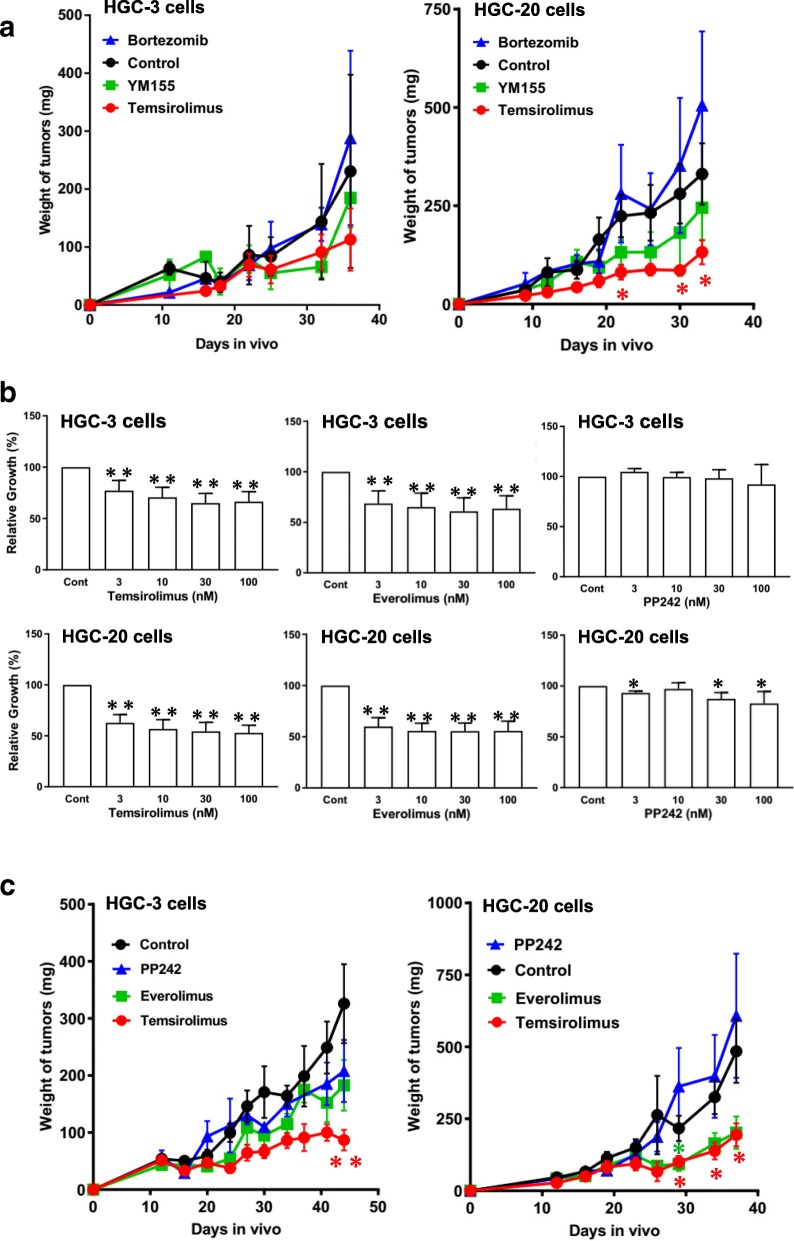
Identification of temsirolimus as a potent growth inhibitor for diffuse-type GC-initiating cells. **a** Effect of temsirolimus, bortezomib, and YM155 on the growth of HGC-3 and -20 cells in vivo, compared with controls (DMSO). **b** Effect of temsirolimus, everolimus, and PP242 on the growth of HGC-3 and -20 cells in primary culture. **c** Effect of temsirolimus, everolimus and PP242 on the growth of HGC-3 and -20 cells in vivo, compared with controls (DMSO). Mean ± SD of three (**a**, **c**) and five (**b**) independent experiments. * *P* < 0.05, ** *P* < 0.01 (Student’s t-test compared with controls (Cont))

We first examined whether the drugs suppressed the growth of PDX cells in vivo, and found that only temsirolimus significantly suppressed it (Fig. 3a). Thus, the focus was on temsirolimus and its analogues for their effects on diffuse-type GC-initiating cells.

### Temsirolimus is the best among mTOR inhibitors examined to suppress the growth of diffuse-type GC-initiating cells

mTOR, or mammalian target of rapamycin, is a central regulatory kinase that regulates the production of proteins involved in key cellular processes such as cell growth, metabolism, and angiogenesis. Suppression of the mTOR pathway has been reported to inhibit the growth of GC cells in vitro and delay tumor progression in animal models [40]. However, the effect of temsirolimus on their growth has not been examined either in vivo or in vitro. In previous studies, everolimus, another mTOR inhibitor, was found not to be effective in improving the overall survival of advanced GC patients [41, 42], although Park et al. reported that the drug was satisfactory in a diffuse-type GC patient with *PIK3CA* mutation [43]. Therefore, the effects of temsirolimus, everolimus, and PP242, a second generation mTOR inhibitor [44], on the growth of diffuse-type GC-initiating cells were evaluated in primary culture and in vivo.

We found that temsirolimus and everolimus significantly suppressed the growth of HGC-3 and -20 cells in vitro, while PP242 had only a weak effect (Fig. 3b). The growth-suppressive action of temsirolimus appeared to be modest, but this was because temsirolimus was not toxic to the cells. Temsirolimus almost completely suppressed the growth of HGC-20 cells in culture (Additional file 1: Figure S8). Temsirolimus significantly suppressed the growth of HGC-3 and -20 cells in vivo and showed stronger activity than other mTOR inhibitors (Fig. 3c). Thus, temsirolimus may be the best among the three mTOR inhibitors examined in the present study.

### p-mTOR expression is a prognostic factor for diffuse-type GC patients

**Fig. 4:**
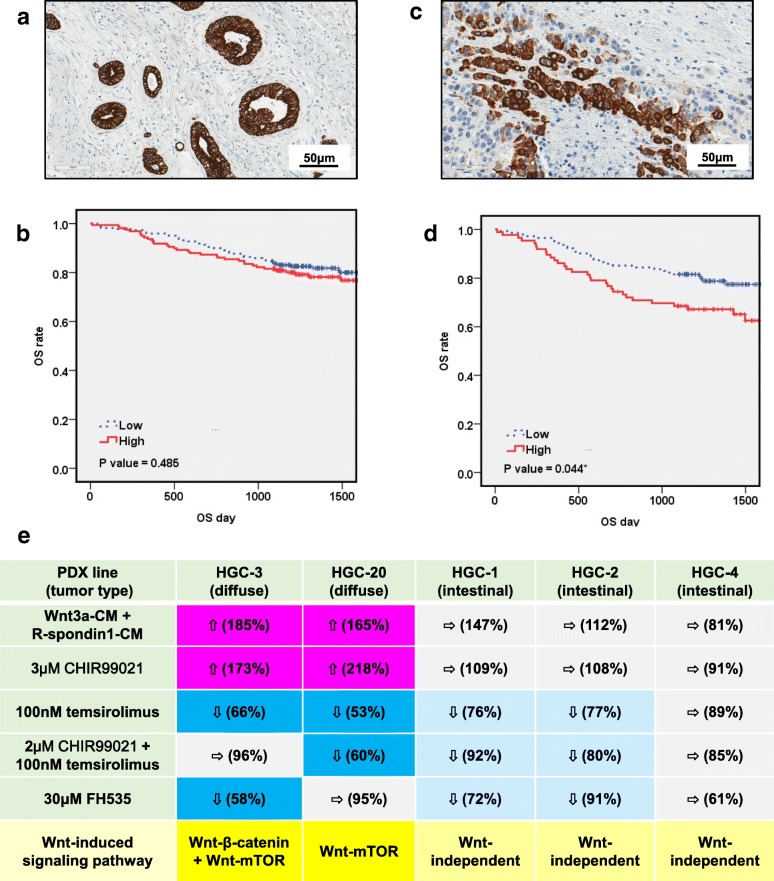
Phosphorylated mTOR expression is a prognostic marker for diffuse-type GC patients, and Wnt-mTOR axis is involved in the growth regulation of diffuse-type GC-initiating cells. **a**, **c** Representative immunohistochemical micrographs of phosphorylated mTOR expressions in primary intestinal-type (**a**) and diffuse-type (**c**) GC tissues. **b**, **d** Kaplan-Meier analyses of overall survival for non-diffuse-type (**b**) and diffuse-type (**d**) GC patients with phosphorylated mTOR expression. Diffuse-type GC patients with strong phosphorylated mTOR expression (solid red line) had a significant poorer outcome than those with weak phosphorylated mTOR expression (dotted blue line) (*P* = 0.044), while no significant differences were found between the two in non-diffuse type GC patients. **e** Wnt-mTOR axis is involved in the growth regulation of diffuse-type GC-initiating cells. Results on effects of temsirolimus, Wnt3a, R-spondin-1, CHIR99021, and FH535 on the growth of diffuse- and intestinal-type GC-initiating cells in primary culture, shown in Additional file 1: Figure S9, and Figures S11 to S14 are summarized. Significant growth stimulation is shown by upward arrows, while significant growth suppression by downward arrows, respectively (no significant changes by horizontal arrows). Strong (more than 151%) growth stimulations are shown by purple, and strong (less than 66%) and weak (67–100%) growth suppressions are shown by blue and light blue, respectively (no significant changes by light gray)

mTOR is an evolutionarily conserved member of the phosphoinositol kinase-related kinase family whose activity is regulated by phosphorylation in response to insulin or muscle activity [45]. Thus, the relationship between the expression of p-mTOR and the prognosis of GC patients was examined immunohistochemically. p-mTOR was detected in both non-diffuse- and diffuse-type GC tissues (Fig. 4a and c). Kaplan-Meier analysis showed that diffuse-type GC patients with strong p-mTOR expression had a significantly worse prognosis than those with weak p-mTOR expression (*n* = 227; *P* = 0.044; Fig. 4d), while significant differences were not found in non-diffuse type GC patients (*n* = 377; *P* = 0.485; Fig. 4b). The relationship between p-mTOR expression and other clinicopathological characteristics of GC cases is summarized in Additional file 2: Table S3. In diffuse-type GCs, p-mTOR was significantly (*P* < 0.044) associated with prognosis on univariate analysis, but not on multivariate analysis (Additional file 2: Table S4). In the total GC cohort, p-mTOR was not significantly associated with prognosis on either univariate or multivariate analyses (Additional file 2: Table S5). These results suggest that mTOR is specifically involved in the prognosis of diffuse-type GC.

### mTOR inhibitors may be useful for the treatment of diffuse-type GC-initiating cells

We compared the effect of temsirolimus on the growth of diffuse- and intestinal-type GC-initiating cells in primary culture. HGC-20 cells were the most sensitive among the cells examined to the growth-suppressive action of temsirolimus. Temsirolimus more strongly suppressed the growth of diffuse-type GC-initiating cells than that of intestinal-type cells (Additional file 1: Figure S9). Considering that diffuse-type GC-initiating cells were more resistant to anti-tumor drugs than intestinal-type cells or established GC cell lines (Additional file 1: Figure S5), these results indicate that mTOR inhibitors may be useful for the treatment of diffuse-type GC-initiating cells.

### Wnt-mTOR axis is involved in the growth regulation of diffuse-type GC-initiating cells

We tried to identify upstream signaling components that activated mTOR. Bartfeld et al. have reported that human gastric epithelial cells require EGF, noggin, R-spondin1 and Wnt3a for their growth [17], and our RNA sequencing analysis showed that the Wnt signaling pathway was up-regulated in HGC-20 cells (Additional file 1: Figure S10). Thus, we compared effect of Wnt3a/R-spondin1 on the growth of diffuse-type GC-initiating cells. Wnt binding to its receptor activates a linear signal transduction cascade, which culminates in the stabilization of β-catenin. However, it has recently become clear that this pathway branches off to trigger a variety of β-catenin-independent pathways including the activation of mTORC1 signaling, regulation of the Hippo effectors YAP and TAZ, and the Wnt-STOP pathway [46]. Thus, the involvement of specific downstream cascades in the Wnt-induced response was assessed according to Acebron and Niehrs [47].

We found that the growth of diffuse-type GC-initiating cells was significantly stimulated by Wnt3a and R-spondin1, while that of intestinal-type cells was not (Additional file 1: Figure S11). CHIR99021, a Wnt signaling activator, significantly stimulated the growth of HGC-3 and -20 cells, while it did not stimulate that of intestinal-type cells (Additional file 1: Figure S12), suggesting that Wnt is specifically involved in the growth regulation of diffuse-type GC-initiating cells. Moreover, CHIR99021-induced growth was partly and completely suppressed by mTOR inhibitor temsirolimus in culture of HGC-3 and -20 cells, respectively (Additional file 1: Figure S13). These results suggest that the growth of diffuse-type GC-initiating cells is regulated by the Wnt-mTOR axis, but that other signaling cascades may function in HGC-3 cells.

FH535, a β-catenin signaling inhibitor, did not affect the growth of HGC-20 cells, but it suppressed that of HGC-3 cells (Additional file 1: Figure S14), indicating that the Wnt signaling is β-catenin-independent in HGC-20 cells, but that it is partly regulated by a β-catenin-dependent pathway in HGC-3 cells. We concluded that the Wnt-mTOR pathway was active in diffuse-type GC-initiating cells, but not in intestinal-type cells. In HGC-3 cells, the Wnt-β-catenin pathway was also active (Fig. 4e).

### Mutation profiles of GCs in TCGA cohort

**Fig. 5:**
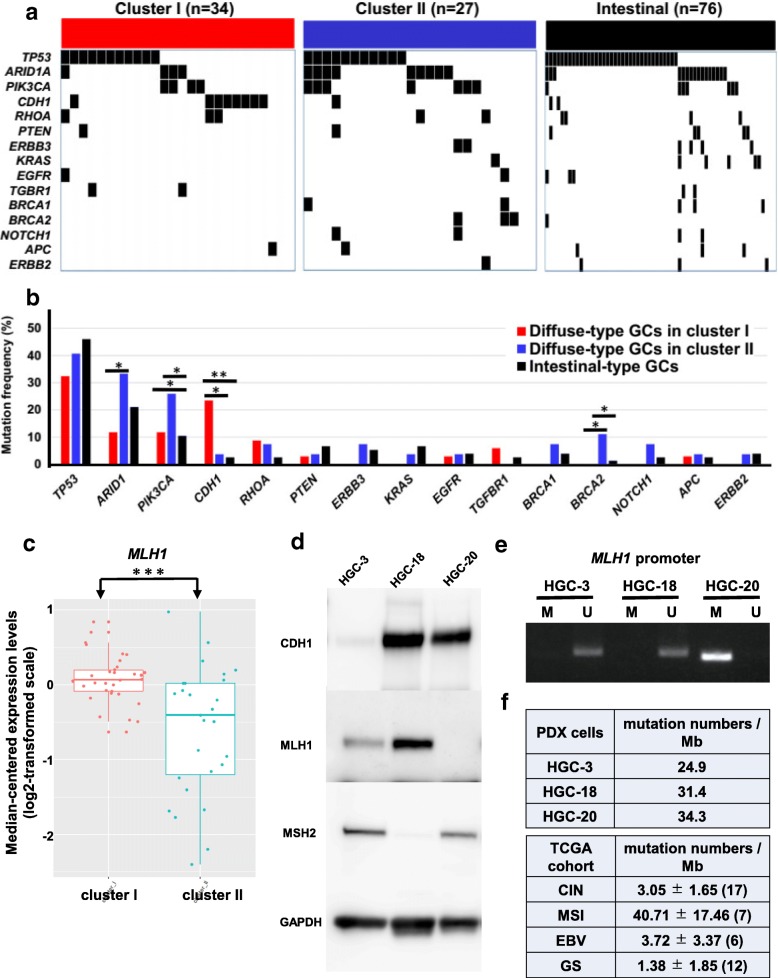
Features of mutations in primary GCs in TCGA cohort and PDX cells. **a** Overview of mutations in diffuse-type GCs in clusters I and II, and intestinal-type GCs in TCGA cohort. Top 15 most frequently mutated genes are shown. **b** Mutation frequencies of the top 15 genes in diffuse-type GCs in clusters I and II, and intestinal-type GCs in TCGA cohort. * *P* < 0.05, ** *P* < 0.01 by Chi-square test between two populations. In the case of *PIK3CA*, mutation frequency in diffuse-type GCs in cluster II (7/27) was significantly greater than that in intestinal-type GCs (7/76; *P* = 0.029), or than that in non-diffuse-type GCs in cluster II (diffuse-type GCs in cluster I + intestinal-type GCs; 11/110; *P* = 0.028). In the case of *CDH1*, mutation frequency in diffuse-type GCs in cluster I (8/34) was significantly greater than that in intestinal-type GCs (2/76; *P* < 0.001), or than that in diffuse-type GCs in cluster II (1/27; *P* = 0.030). **c** Expression of *MLH1* in diffuse-type GCs in cluster I and II in TCGA cohort. *** *P* < 0.001 by two sample t-test. **d** Western blot analysis of CDH1, MLH1, and MSH2 in HGC-3, − 18, and − 20 PDXs. GAPDH was used as a loading control. **e** Methylation-specific polymerase chain reaction analysis of *MLH1* promoter region in HGC-3, − 18, and − 20 PDXs. PCR products recognizing methylated (lanes M) and unmethylated (lanes U) CpG sites were loaded in 2.5% agarose gels. **f** Mutation numbers/Mb in HGC-3, − 18, and − 20 cells, and in CIN, MSI, EBV, and GS subtype GCs in TCGA cohort

We compared mutation profiles of diffuse-type C-I and C-II GCs and intestinal-type GCs in TCGA. We found that *ARID1A*, *PIK3CA, and BRCA2* were significantly more frequently mutated in diffuse-type C-II GCs than in diffuse-type C-I or intestinal-type GCs, while mutations in *CDH1* were significantly more frequently found in diffuse-type C-I GCs (23.5%) than in diffuse-type C-II (3.7%) or intestinal-type GCs (2.6%) (Fig. 5a and b).

Cho et al. reported that somatic mutations in *CDH1 or TGFBR1* were more frequently found in early-onset diffuse-type GCs [48]. Our results that *CDH1* and *TGFBR1* mutations were exclusively found in diffuse-type C-I GCs (Fig. 5b) suggest that diffuse-type C-I GCs have features of early-onset diffuse-type GCs. Consistent with this, we found that patients with diffuse-type C-I GC were significantly younger than those with intestinal-type GC in TCGA cohort (Additional file 1: Figure S15), though we could not find differences between various GCs on the male-to-female ratio.

ARID1A was shown to recruit mismatch repair protein MSH2 to chromatin during DNA replication and to promote mismatch repair. Conversely, ARID1A deficiency correlated with MSI genomic signature, and increased mutation load across multiple human cancer types [49]. BRCA1/2 are tumor suppressors involved in pathways important for regulating DNA damage, and *BRCA2* mutations were found to be associated with familial aggregations of not only breast but also of stomach cancers [50]. Then, the results that *ARID1A and BRCA2* were more frequently mutated in diffuse-type C-II GCs than in C-I GCs (Fig. 5b) suggest that many mutations may be induced in the tumor by the loss of function of these tumor suppressors. Consistent with this, we found that the expression level of *MLH1,* which encodes a protein involved in DNA mismatch repair, was significantly (*P* < 0.001) reduced in diffuse-type C-II GCs than in C-I GCs in TCGA cohort (Fig. 5c). We expected that some diffuse-type C-II GCs might be MSI type with defects in DNA mismatch repair system.

We found that *PIK3CA* was more frequently mutated in diffuse-type C-II GCs than in diffuse-type C-I or intestinal-type GCs (Fig. 5b). Janku et al. [51] reported that *PIK3CA* mutations were detected in 11.5% of patients with diverse solid tumors, and that the response rate was significantly higher for patients with *PIK3CA* mutations treated with PI3K/AKT/mTOR pathway inhibitors than for those without documented mutations (35% vs 6%). Thus, diffuse-type C-II GCs may be more responsive to temsirolimus than other GCs.

### Mutations and epigenetic gene regulation in PDX cells

We also analyzed mutations and copy number variations in PDX cells. A mutation and a copy number gain in *PIK3CA* were found in HGC-20 and -3 PDXs, respectively (Additional file 2: Table S6). We found that HGC-20 cells were more responsive to temsirolimus than HGC-3 cells (Additional file 1: Figure S9). This may be related to that *PIK3CA* was mutated in HGC-20 cells.

Mutations in *ARID1A* were found in HGC-20 and -3 PDXs, and mutations in *CDH1* and *TP53* were found only in HGC-3 PDX (Additional file 2: Table S6). Consistent with this, Western blot analysis showed that E-cadherin was expressed in HGC-18 and -20 cells, but not in HGC-3 cells (Fig. 5d).

MLH1 and MSH2 were not expressed in HGC-20 and -18 cells, respectively (Fig. 5d). Methylation-specific polymerase chain reaction analysis showed that *MLH1* promoter was methylated in HGC-20 cells, while it was not methylated in HGC-3 and -18 cells (Fig. 5e), indicating that *MLH1* is epigenetically silenced in HGC-20 cells. A frameshift mutation in *MSH2* was found in HGC-18 cells (Additional file 2: Table S6), which may result in the loss of its expression. These results are consistent with the results that *ARID1A* and *BRCA2* were more frequently mutated (Fig. 5b), and expression of *MLH1* was suppressed in diffuse-type C-II GCs in TCGA cohort (Fig. 5c), because PDX cells were classified into diffuse-type C-II GCs (Fig. 2b). These results also indicate that expression of DNA mismatch repair proteins are suppressed by genetic and/or epigenetic mechanisms during the carcinogenesis process of diffuse-type C-II GCs.

### HGC-3, -18, and -20 cells retain features of MSI or CIN subtypes

In a previous study by TCGA researchers, GCs were classified into EBV, MSI, GS, or CIN subtypes [28], and the clinical significance of these GC subtypes has been reported [20]. We therefore attempted to identify the molecular subtypes of HGC-3, -18, and -20 cells using TCGA dataset. To construct the precise prediction model, a Bayesian compound covariate prediction-based classification algorithm was developed with the RNA-sequencing data of the cells in primary culture, and the accuracy of subtype prediction was estimated (Additional file 1: Figure S16). When the data of the cells were applied to the MSI or CIN subtype predictors, all three samples were predicted as MSI (> 93.3%) or CIN (> 90.7%) subtypes. On the other hand, when the EBV or GS subtype prediction model were applied to the cells, all samples were not classified into EBV or GS subtypes with at least 99% probability (Additional file 1: Figure S16e). These results indicate that HGC-3, -18, and -20 cells had shared molecular characteristics of MSI or CIN subtypes.

### HGC-18 and -20 cells are MSI-H, and HGC-3 cells are CIN

Tumor mutation burden can be accurately assessed by comprehensive genomic profiling assay targeting ~ 1 Mb of coding genome [52]. Numbers of mutated genes in HGC-3, -18, and -20 cells were estimated to be about 1244, 1569, and 1717, respectively, using data obtained with the Ion Comprehensive Cancer Panel. The mutation numbers/Mb were similar between PDX cells and MSI subtypes in TCGA cohort, and their numbers were far greater than those of CIN, EBV, and GS subtypes (Fig. 5f). These results suggest that PDX cells have a feature of MSI subtype.

HGC-3, -18 and -20 cells were tested for microsatellite status, as described previously [26]. We found allelic shifts in the three microsatellites in the panel in both HGC-18 and -20 cells, but not in HGC-3 cells (Additional file 1: Figure S17). Chromosome number analysis showed that aneuploid cells were often found in HGC-3 cells in primary culture (Additional file 1: Figure S18), indicating that they are chromosome unstable. Thus, we concluded that HGC-18 and -20 cells were MSI-H, and HGC-3 cells were CIN, consistent with the results obtained with the prediction model (Additional file 1: Figure S16e). Usually CIN GCs have a small number of mutations, but HGC-3 cells had many (Fig. 5f). Ben-David et al. reported rapid accumulation of copy number alterations during PDX passages [53]. Thus, HGC-3 may have many mutations because we used HGC-3 cells at later passages.

### Diffuse-type GCs in cluster II may develop from intestinal-type GCs while diffuse-type GCs in cluster I from normal gastric epithelial cells

**Fig. 6:**
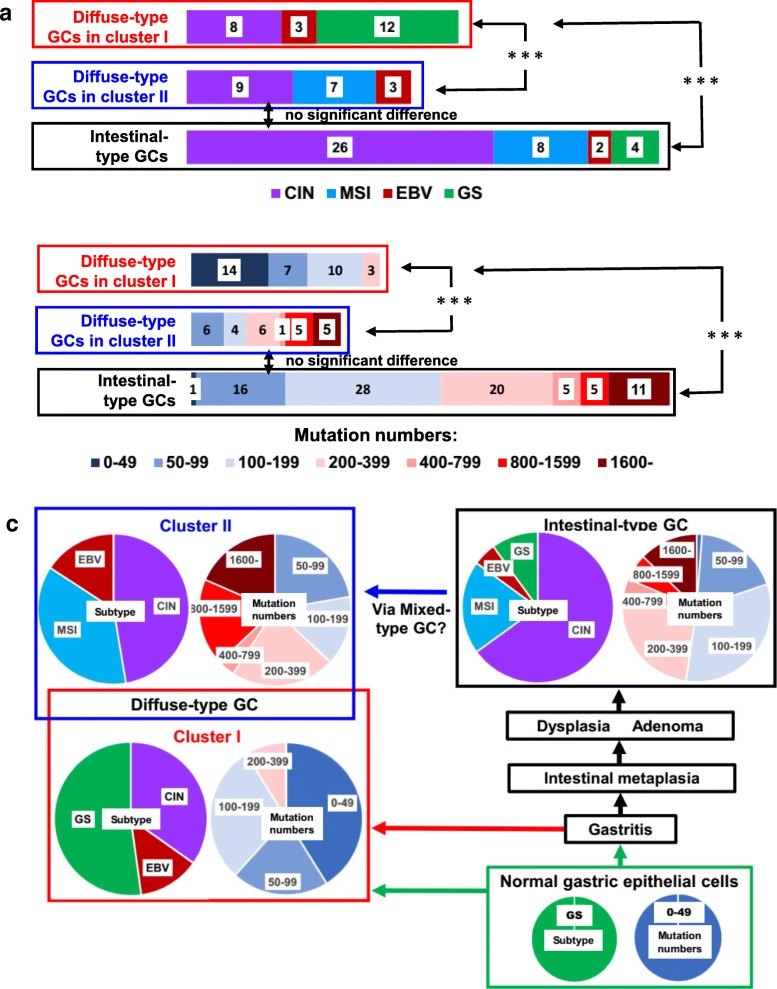
A hypothesis on the relationship between diffuse-type GCs in clusters I and II, and intestinal-type GCs in TCGA cohort. **a** Distribution of CIN, MSI, EBV, and GS subtypes in diffuse-type GCs in clusters I and II, and intestinal-type GCs in TCGA cohort. *** *P* < 0.001 (Chi-square test between two populations). **b** Distribution of GC cells with various mutation numbers in diffuse-type GCs in clusters I and II, and intestinal-type GCs in TCGA cohort. *** *P* < 0.001 (Chi-square test between two populations). **c** A hypothesis on the development of diffuse-type GCs in clusters I and II, and intestinal-type GCs from normal gastric epithelial cells

We found in the present study that expression of DNA mismatch repair proteins was suppressed in diffuse-type C-II GCs, and that PDX cells were MSI or CIN. These results are not consistent with TCGA report that GS subtype is enriched (73%), and MSI and CIN subtypes are minor in diffuse-type GCs [28]. We checked TCGA dataset, and found that classification of GCs has been changed greatly after their paper was published in 2014. Forty-four cases were removed from diffuse-type GCs, 21 cases were changed from non-diffuse- to diffuse-type GCs, and 21 new diffuse-type GC cases were added. Subtypes of newly added GC cases are not described in TCGA. At present, 42 diffuse-type GC cases are available for subtype analysis in TCGA, composed of 17 CIN, 12 GS, 7 MSI, and 6 EBV cases. We examined how four TCGA subtypes were included in diffuse-type C-I and C-II GCs. We found that GS and MSI subtypes were exclusively included in diffuse-type C-I and C-II GCs, respectively (Fig. 6a), and that the distribution of four subtypes was significantly (*P* < 0.001) different between two clusters. Then, our results are consistent with TCGA subtype classification. We examined the distribution of four subtypes in intestinal-type GCs, and found that the CIN and MSI subtypes were predominant in intestinal-type GCs, similar to diffuse-type C-II GCs, but their distribution was significantly (*P* < 0.001) different from that of C-I GCs (Fig. 6a).

Data on the number of mutations in primary GCs are available in TCGA. We compared mutation numbers of diffuse-type C-I and C-II GCs, and found that those of diffuse-type C-I GCs (mean = 88, range = 0~358, *n* = 34) were significantly (*p* < 0.001) smaller than those of diffuse-type C-II GCs (mean = 748, range = 61~3651, *n* = 27) in TCGA cohort (Fig. 6b). In addition, the mutation numbers of intestinal-type GCs (mean = 504, range = 52~5923, *n* = 76) were similar to those of diffuse-type C-II GCs, and they were significantly (*p* < 0.001) greater than those of diffuse-type C-I GCs (Fig. 6b).

The ratio of four subtypes and the ratio of GCs with various mutation numbers are summarized in Fig. 6c. Both ratios were significantly different between diffuse-type C-I and C-II GCs, and between diffuse-type C-I and intestinal-type GCs, but both were very similar between diffuse-type C-II GCs and intestinal-type GCs. Considering that histologic type was often altered from intestinal- to diffuse-type with progression of the tumor [4], and that more than 20% of GCs were mixed-type composed of intestinal- and diffuse-type GCs [6, 7], we speculated that diffuse-type GCs were composed of two populations with distinct developmental pathways; some diffuse-type C-II GCs may develop from intestinal-type GCs while diffuse-type C-I GCs from normal gastric epithelial cells (Fig. 6c).

### PD-L1 is expressed by some diffuse-type GCs and is regulated by mTOR and cell-cell interaction

In the present study, we found that diffuse-type C-II GC-initiating cells including HGC-3 and -20 cells had features of MSI or CIN subtypes with many mutations (Fig. 5f, Additional file 1: Figure S16e). We and others have reported that MSI GC cells express PD-L1 [54]. Expression of PD-L1 has been reported to be associated with poor prognosis [55], and programmed death-1 /PD-L1 pathway is now considered an important target for the treatment of GCs [56]. Moreover, several researchers have reported that expression of PD-L1 is regulated by mTOR in several tumors [57]. We thus examined whether diffuse-type GCs express PD-L1 and whether their expression is regulated by mTOR.

**Fig. 7:**
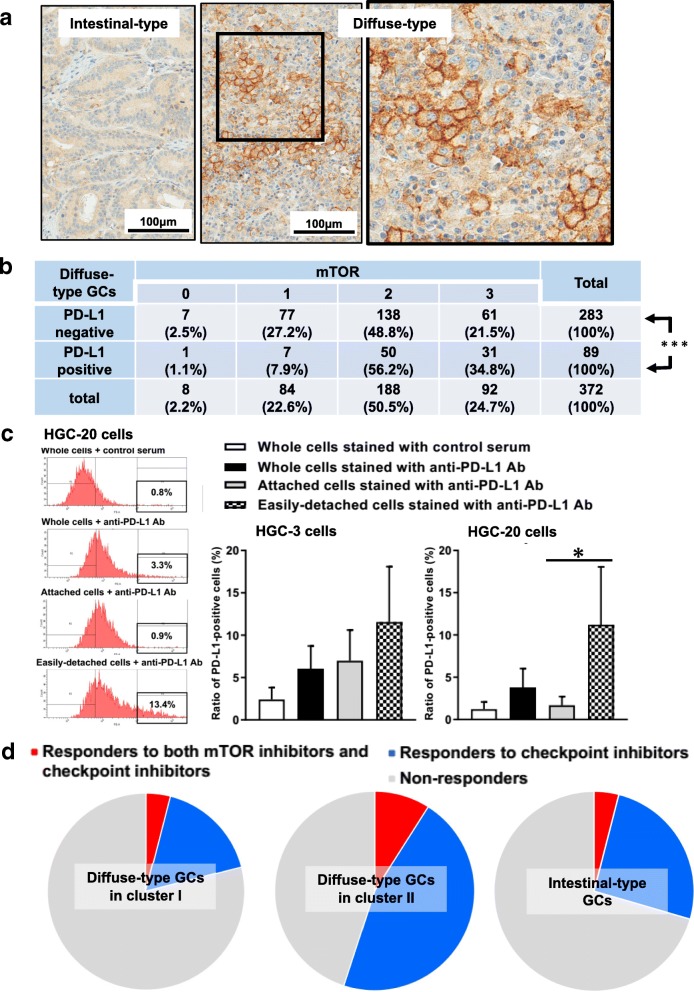
PD-L1 expression in GCs, and estimation of responders to immune checkpoint inhibitors and mTOR inhibitors in patients with various GCs. **a** Light micrographs showing PD-L1 expressions in intestinal- and diffuse-type GCs. An area shown by a square in the diffuse-type GC is enlarged on the right. **b** The relationship between PD-L1 and mTOR expressions in diffuse-type GCs. A significant difference was found between PD-L1-positive and -negative patients on the expression of mTOR. *** *P* < 0.001 by Chi-square test between two populations. **c** The ratio of PD-L1-positive cells in whole (attached + easily-detached), attached, and easily-detached cell fractions in HGC-3 and -20 cells. Percentages of PD-L1-positive cells in the rectangle in the left figures were determined, and compared. Mean ± SD of five independent experiments. * *P* < 0.05 by two sample t-test. **d** Estimation of responders to immune checkpoint inhibitors and mTOR inhibitors in patients with various GCs. Since all *PIK3CA* mutated GCs were EBV or MSI subtypes in TCGA cohort, and all patients with EBV or MSI subtype GC were responders to checkpoint inhibitors, all responders to mTOR inhibitors were responders to checkpoint inhibitors

We found that most intestinal-type GC cells expressed PD-L1 homogenously, while diffuse-type GC cells expressed it heterogeneously (Fig. 7a). We compared *PD-L1* expression levels in diffuse-type C-I and C-II GCs in TCGA cohort, and we found that *PD-L1* was expressed by GCs in both clusters (Additional file 1: Figure S19). We could not find significant differences between them though strong PD-L1 expression (> 1) was found only in diffuse-type C-II GCs. Tissue array analysis showed that about 24% (89/372) of diffuse-type GCs expressed PD-L1 (Fig. 7b), consistent with our previous report. This indicates that some diffuse-type C-II GCs express PD-L1 strongly. A significant correlation was found between mTOR and PD-L1 expressions (Fig. 7b), suggesting that mTOR is involved in the regulation of PD-L1 in diffuse-type GCs.

FACS analysis showed that about 3–7% of HGC-3 and -20 cells expressed PD-L1 strongly, and PD-L1 expression was suppressed by temsirolimus in HGC-3 cells, but not in HGC-20 cells (Additional file 1: Figure S20).

We found that the ratio of PD-L1-positive cells in HGC-20 cells was decreased by washing. Further analysis showed that PD-L1-positive cells were enriched in the easily-detached cell fraction (about 15% of total cells) in HGC-20 cells, but this was not found in HGC-3 cells (Fig. 7c). PD-L1-positive cells were also enriched in the easily-detached cell fraction in MKN45 cells (Additional file 1: Figure S21). As stated previously, diffuse-type GC-initiating cells formed KLSs when maintained for weeks in culture (Fig. 1k, Additional files 3, 4, 5, 6, 7: Movies S1-S5), and KLS-forming cells were included in the easily-detached cell fraction. This indicates that KLS-forming cells express PD-L1 strongly, and that PD-L1 expression is regulated by cell-cell interactions in these cells. It has been repeatedly reported that PD-L1-expressing cells are more resistant to chemotherapy [58, 59]. Then, KLS-forming PD-L1-expressing HGC-20 cells may be more resistant to temsirolimus than attached HGC-20 cells or HGC-3 cells. If the hypothesis is right, it may be natural that KLS-forming HGC-20 cells were more resistant to temsirolimus than HGC-3 cells on the expression of PD-L1 (Additional file 1: Figure S20).

Considering that PD-L1 overexpression is significantly related to lymph-node invasion and poor prognosis in GC patients [55], KLS-forming cells may be an important target for the development of new therapies against diffuse-type GCs.

### Patients with diffuse-type GCs in cluster II may be responders to immune checkpoint inhibitors

In the present study, we found that MSI GCs were exclusively included in diffuse-type C-II GCs in TCGA cohort (Fig. 6a). It is well established that tumor mutation burden is strongly correlated with response to therapy with immune checkpoint inhibitors [60]. Kim et al. recently reported that in metastatic GCs, strong responses to programmed death-1 inhibition were found in all cases with MSI and EBV subtype GCs, while responses were found only in 5 and 12% of cases with CIN and GS subtype GCs, respectively, by using TCGA subtype classification [61]. We combined their results with our analysis shown in Fig. 6a to estimate the ratio of responders to checkpoint inhibitors in GC patients. We found that about 21, 55, and 29% of patients with diffuse-type C-I, C-II, and intestinal-type GCs, respectively, may be responders to the immunotherapy (Additional file 1: Figure S22). This indicates that the immune checkpoint inhibitors may be useful for the treatment of diffuse-type C-II GCs, but may not good for C-I GCs.

### mTOR inhibitors and checkpoint inhibitors may be useful for the treatment of a subset of diffuse-type GC patients

As shown in Fig. 5b, about 11, 26, and 10.5% of diffuse-type C-I, C-II, and intestinal-type GCs exhibited *PIK3CA* mutations in TCGA cohort, and the overall responsive rate was reported 35% for cancer patients with *PIK3CA* mutations treated with PI3K/AKT/mTOR pathway inhibitors [51]. By combining these results, we estimated that about 4, 9, and 4% of the respective GCs may be responders to mTOR inhibitors. *PIK3CA* mutations were frequently found in MSI GCs [62]. Thus, responders to mTOR inhibitors may also be responders to immune checkpoint inhibitors. In TCGA cohort, we found that all GCs with *PIK3CA* mutations were MSI or EBV subtypes in diffuse-type C-I (3/3), C-II (6/6), and intestinal-type GCs (5/5). This is consistent with previous reports that *PIK3CA* mutations are significantly more frequently found in MSI and EBV subtype GCs [28, 62–64]. Our results also indicates that responders to mTOR inhibitors are also responders to immune checkpoint inhibitors. We thus estimated that about 4, 9, and 4% of patients with respective GCs are responders to both inhibitors (Fig. 7d). We concluded that both inhibitors may be useful for the treatment of a subset of diffuse-type GC patients.

## Discussion

In the present study, we established PDX lines from diffuse-type GCs, and found that the features of diffuse-type and mixed-type GC-initiating cells were different from those of intestinal-type cells. We also found that HGC-20 cells formed scirrhous-type tumors (Fig. 1). There are many papers on the identification of GC stem cells [16, 65–68], but to the best of our knowledge, the formation of scirrhous-type tumors has not been reported when cultured GC-initiating cells were implanted into immuno-deficient animals. HGC-20 cells may thus be useful to analyze how scirrhous-type tumors are formed and to identify drugs that regulate their growth. In the present study, we found that all three diffuse-type PDX lines examined were MSI or CIN subtypes, inconsistent with TCGA report. It remains to be examined whether mTOR inhibitors are useful for the treatment of diffuse-type GCs with other phenotypes. More experiments are needed to examine it with more PDX lines.

Integrative gastric subtyping studies have been reported from several groups, [28, 69, 70], but diffuse-type GCs have not been classified into subtypes, except recent papers where diffuse-type GCs were classified into early-onset and late-onset cancers [48], and they were classified into three types based on proteome analysis [71]. In the present study, we classified diffuse-type GCs into two subtypes by hierarchical cluster analysis, and we obtained strong evidence that diffuse-type C-II GCs develop from intestinal-type GCs, and diffuse-type C-I GCs from normal gastric epithelial cells (Fig. 6c), by mutation profile, subtype analysis, and mutation number analysis. Our results are consistent with those of Cho et al. [48] that mutations in *CDH1* and *TGFBR1* were exclusively found in early-onset diffuse-type GCs, and indicate that early-onset diffuse-type GCs may develop from normal gastric epithelial cells. We speculate that some late-onset diffuse-type GCs may be formed via intestinal-type GCs. This may be the first report suggesting that some diffuse-type GCs may develop from intestinal-type GCs, though this idea has long been suggested by histological analysis [4]. We found in this study that expression of DNA mismatch repair proteins was suppressed by genetic and/or epigenetic mechanisms in diffuse-type C-II GCs. This may be a cause why MSI GCs were exclusively found in this cluster. Further analyses are needed to clarify why such changes are induced in GCs during the development and progression of tumors.

By screening drugs that suppress the growth of diffuse-type GC-initiating cells, we identified mTOR inhibitor temsirolimus as a potentially useful drug. This is consistent with previous reports that mTOR plays an important role in the growth regulation of GCs [22, 40, 72, 73]. In a previous study, mTOR inhibitor everolimus was effective for a subset of diffuse-type GC patients [43], though it was not effective for overall GC patients [41, 42]. We found *PIK3CA* mutations more frequently in diffuse-type C-II GCs (Fig. 5b), indicating that a subset of diffuse-type GCs may be responders to mTOR inhibitors. In the present study, temsirolimus was superior to everolimus in suppressing the growth of diffuse-type GC-initiating cells in vivo. Both temsirolimus and everolimus selectively inhibit mTOR signaling with similar molecular mechanisms, but with distinct clinical profiles [72]. It remains to be determined whether temsirolimus is useful for the treatment of diffuse-type GC patients.

In the present study, we found that expression of p-mTOR was significantly correlated with clinical aggressiveness and worse prognosis in diffuse-type GC patients, but such relationship was not found in non-diffuse-type GC patients (Fig. 4b, d). By analyzing the behavior of PDX cells in primary culture, we found that the Wnt-mTOR pathway was strongly involved in the growth regulation of diffuse-type GC-initiating cells (Fig. 4e). This may be the first report suggesting that mTOR functions differently between intestinal- and diffuse-type GC-initiating cells.

Carneiro et al. [74] reported that β-catenin was not found in the in situ carcinomas of *E-cadherin* mutation carriers with diffuse-type GC, and we previously found that poorly differentiated carcinoma cells were β-catenin-negative in a mouse diffuse-type GC model [75]. Together, these observations suggest that the growth of diffuse-type GC is regulated by a β-catenin-independent pathway in both humans and mice. In contrast, nuclear accumulation of β-catenin was specifically found in intestinal-type GC from early clinical stages [76], and growth of intestinal-type GC seems to be regulated by β-catenin-dependent pathway. Frequent somatic mutation of the APC and loss of heterozygosity on chromosome 5q, where the APC is located, have been frequently detected in intestinal-type GC but not in diffuse-type GC [77, 78], and somatic mutations of the β-catenin gene is exclusively detected in intestinal-type GC [79]. Our results are consistent with these previous reports, and suggest that Wnt-mTOR axis may play an essential role in the development of some diffuse-type GC.

Diffuse-type GCs have been considered non-responders to the checkpoint inhibitors because GS subtypes were reported to be major in them [28]. In the present study, we found that GS and MSI subtypes were exclusively included in diffuse-type C-I and C-II GCs, respectively (Fig. 6a). By combining results that the ratio of responders to immune checkpoint inhibitors was significantly associated with TCGA subtypes [61], we estimated that more than 50% of patients with diffuse-type C-II GC were responders to the immunotherapy (Fig. 7d). We hope that our research will be useful for the development of targeted therapies against diffuse-type GC.

## Conclusions

Previous studies suggested that mTOR inhibitors and checkpoint inhibitors were not effective for the treatment of diffuse-type GCs. Diffuse-type GCs could be classified into two clusters with different developmental pathways. Importantly, we obtained evidence in this study that these inhibitors may be useful for the treatment of a subset of diffuse-type GC.

## Additional files

Additional file 1: **Figure S1.** Organoid formation by non-tumor gastric epithelial cells. Phase contrast micrographs of the cells on days 7, 9, 14, and 18 (a scratch with an arrow indicates that the same organoid was photographed), and light micrographs showing an organoid consisted of gastric epithelial cells with various degrees of differentiation. Alcian blue (pH2.5)-PAS-hematoxylin. **Figure S2.** Growth of GC-initiating cells in REBM-based medium (R-medium) and in ADF-based medium (A-medium). Mean ± SD of five independent experiments. **, *P* < 0.01 by Student’s t-test compared with cell numbers in the A-medium. **Figure S3.** Expression of stem cell marker genes in HGC cells. **Figure S4.** Changes in the ratio of CD49f^high^ cells in culture. Significant increases in the ratio of CD49f^high^ cells were found in culture of intestinal-type (HGC-1, -2, and -4) cells. In culture of diffuse- and mixed-type (HGC-3, − 18, and − 20) cells, the ratio was significantly increased only in HGC-20 cells. Mean ± SD of five independent experiments. *, *P* < 0.05; **, *P* < 0.01 by Student’s t-test between before and after cultivation. **Figure S5.** HGC-3, -18, and -20 PDX cells, and MKN45 and NUGC-4 cells were treated with various concentrations of 5-fluorouracil (5-FU), doxorubicin and doxifluridine in REBM-based medium, and cell numbers were determined on day 14 by MTT assay. Mean ± SD of five independent experiments. *, *P* < 0.05; **, *P* < 0.01 by Student’s t-test compared with MKN45 cells (pink asterisks), and with NUGC-4 cells (blue asterisks) at corresponding drug concentrations. **Figure S6.** Phase contrast micrographs of HGC-3 and -20 cells in Matrigel, on day 14 and on day 19, respectively, in culture. **Figure S7.** Identification of drugs that suppress the growth of HGC-20 cells in primary culture. **Figure S8.** HGC-20 cells were seeded on day 0, drugs were added on day 1, and cell numbers were determined by MTT assay on day 5. If a drug completely suppresses the cell growth without toxic effect, the cell number on day 5 should be equal to that on day 1. Temsirolimus at 3 μM (3000 nM) completely suppressed the growth of HGC-20 cells but was not toxic to the cells because the cell number on day 5 was nearly equal to that on day 1. In contrast, bortezomib at 30 nM not only completely inhibited their growth but also was toxic to the cells because the cell number was decreased to almost zero on day 5. Mean ± SD of five independent experiments. *, *P* < 0.05; **, *P* < 0.01 by Student’s t-test compared with controls (Cont). **Figure S9.** Effect of temsirolimus on the growth of diffuse- and intestinal-type GC-initiating cells in primary culture. Mean ± SD of 5 independent experiments. *, *P* < 0.05; **, *P* < 0.01 by Student’s t-test compared with controls (Cont; black asterisks), with HGC-3 (blue asterisks), and with HGC-20 cells (pink asterisks) at corresponding drug concentrations. **Figure S10.** RNA sequencing analysis of HGC-20-specific gene set, showing that Wnt-signaling pathway (shown by red square) is enriched in it. **Figure S11.** Effect of Wnt3a- and R-spondin1-CM (+CM) on the growth of diffuse- and intestinal-type GC-initiating cells in primary culture. Mean ± SD of five independent experiments. **, *P* < 0.01 by Student’s t-test compared with cell numbers in basal medium without CM (basal). **Figure S12.** Effect of CHIR99021 on the growth of diffuse- and intestinal-type GC-initiating cells in primary culture. Mean ± SD of five independent experiments. *, *P* < 0.05; **, *P* < 0.01 by Student’s t-test compared with controls (Cont; black asterisks), with HGC-3 (blue asterisks), and with HGC-20 cells (pink asterisks) at corresponding drug concentrations. **Figure S13.** Effect of temsirolimus on the CHIR99021 (2 μM)-induced growth of diffuse- and intestinal-type GC-initiating cells in primary culture. Mean ± SD of five independent experiments. *, *P* < 0.05; **, *P* < 0.01 by Student’s t-test compared with controls (Cont; black asterisks) and with HGC-20 cells (pink asterisks) at corresponding drug concentrations. **Figure S14.** Effect of FH535 on the growth of diffuse- and intestinal-type GC-initiating cells in primary culture. Mean ± SD of five independent experiments. *, *P* < 0.05; **, *P* < 0.01 by Student’s t-test compared with controls (Cont). **Figure S15.** Features of GC patients in TCGA cohort. **a** Patients with intestinal-type GCs were significantly older than those with diffuse-type GCs in cluster I. *, *P* < 0.05 by Student’s t-test. **b** There was no difference between patients with intestinal- or diffuse-type GCs on the ratio of male to females. **Figure S16.** Prediction of molecular subtype of diffuse- and mixed-type GC-initiating cells using TCGA data set. **a** A heat map of gene expression patterns in EBV, MSI, GS and CIN subtypes in TCGA samples and diffuse- and mixed-type GC-initiating cells (HGC-20, -3, and -18 cells). **b** Schematic diagram of TCGA subtype prediction model. A decision tree approach was employed for categorizing patients in test cohorts into the four subtypes of GCs. **c** The performance of a Bayesian compound covariate prediction classifiers during cross-validation in TCGA dataset. **d** The Bayesian probability of each signature was plotted for sensitivity and specificity. **e** Results of subtype prediction. **Figure S17.** MSI chromatogram for the microsatellites BAT25, BAT26, and BAT40. DNAs from HGC-18 and -20 cells exhibited allelic shifts for these three microsatellites compared with DNAs from non-tumor gastric epithelial cells, while no allelic shift was found with DNAs from HGC-3 cells. **Figure S18.** Chromosome number analysis of HGC-3 cells in primary culture. **Figure S19.** Expression of *PD-L1* in diffuse-type GCs in clusters I and II in TCGA cohort. Strong *PD-L1* expression (> 1) was found only in diffuse-type GCs in cluster II, but statistical significance was not observed between the two clusters. *P* = 0.1 by two sample t-test. **Figure S20.** Effect of temsirolimus (TEM) on the ratio of PD-L1-positive cells in HGC-3 and -20 cells. Percentages of PD-L1-positive cells in the rectangle in the left figures were determined, and compared. Mean ± SD of at least 5 independent experiments. *, *P* < 0.05 by two sample t-test. **Figure S21.** PD-L1-positive cells were enriched in easily-detached cell fraction in MKN45 cells. Percentages of PD-L1-positive cells in the rectangle in the left figures were determined, and compared. In NUGC-4 cells which expressed PD-L1 very weakly, significant difference was not found between cell fractions. Mean ± SD of five independent experiments. *, *P* < 0.05 by two sample t-test. **Figure S22.** Estimation of responders to immune checkpoint inhibitors in patients with various GCs. Ratios of GC subtypes in each cluster were obtained from Fig. 6a, and ratio of responders in each subtype was obtained from Kim et al. [61]. “Estimated ratio of responders” was calculated by multiplying “Ratio of GC subtypes” and “Ratio of responders in subtypes”. The ratio of responders was estimated by adding “Estimated ratios of responders”. (PDF 3804 kb)

Additional file 2: **Table S1.** Clinicopathological features of diffuse-type GC patients whose cancers were used to establish PDX lines. **Table S2.** Differentially expressed 1048 genes in diffuse- and mixed-type GC-initiating cells compared with established cell lines. **Table S3.** Clinicopathological features of 610 GC cases used to prepare tissue array analysis. p-mTOR expression was significantly associated with venous invasion (*P* = 0.045) and perineural invasion (*P* = 0.011) in total cases, and perineural invasion in non-diffuse carcinomas (*P* = 0.027). *P* values were determined by Pearson’s chi-square test. The Spearman rank correlation analysis was used for pT and pTMN stages. **Table S4.** Univariate and multivariate survival analyses of diffuse-type GC (227 cases). p-mTOR was significantly associated with prognosis in univariate analysis, but it was not significantly associated with prognosis in multivariate analysis. P values were determined by Pearson’s chi-square test. The Spearman rank correlation analysis was used for pT and pTMN stages. **Table S5.** Univariate and multivariate survival analyses of total gastric carcinomas (610 cases). p-mTOR was not significantly associated with prognosis in univariate or multivariate analyses. P values were determined by Pearson’s chi-square test. The Spearman rank correlation analysis was used for pT and pTMN stages. **Table S6.** Candidate driver gene mutations and copy number variations in PDX cells. Please refer to https://www.ncbi.nlm.nih.gov/clinvar/variation/12582/ for pathogenic (#1), https://www.ncbi.nlm.nih.gov/clinvar/variation/24832/ for pathogenic (#2), https://www.ncbi.nlm.nih.gov/clinvar/variation/12580 for pathogenic (#3), and https://www.ncbi.nlm.nih.gov/clinvar/variation/39706/ for pathogenic (#4). (PDF 406 kb)

## Abbreviations

ADF: Advanced Dulbecco’s modified Eagle medium /F12
CCLE: The Cancer Cell Line Encyclopedia
C-I GCs: GCs in cluster I
C-II GCs: GCs in cluster I
CIN: Chromosome instablility
CM: Conditioned medium
EBV: Epstein-Barr virus
GC: Gastric cancer
GS: Genomically stable
KLS: Kelp-like structure
MSI: Microsatellite instability
mTOR: Mechanistic target of rapamycin
PD-L1: Programmed cell death-ligand 1
PDX: Patient-derived xenograft
REBM: Renal Epithelial Basal Medium
TCGA: The Cancer Genome Atlas
