# Supplementary material for: A subset of diffuse-type gastric cancer is susceptible to mTOR inhibitors and checkpoint inhibitors
Source: J Exp Clin Cancer Res. 2019 Mar 12;38:127. doi: 10.1186/s13046-019-1121-3 (PMC6416873; doi:10.1186/s13046-019-1121-3)
Supplement: Supplementary file 6 — Figure S1. Organoid formation by non-tumor gastric epithelial cells. Phase contrast micrographs of the cells on days 7, 9, 14, and 18 (a scratch with an arrow indicates that the same organoid was photographed), and light micrographs showing an organoid consisted of gastric epithelial cells with various degrees of differentiation. Alcian blue (pH2.5)-PAS-hematoxylin. Figure S2. Growth of GC-initiating cells in REBM-based medium (R-medium) and in ADF-based medium (A-medium). Mean ± SD of five independent experiments. **, P < 0.01 by Student’s t-test compared with cell numbers in the A-medium. Figure S3. Expression of stem cell marker genes in HGC cells. Figure S4. Changes in the ratio of CD49fhigh cells in culture. Significant increases in the ratio of CD49fhigh cells were found in culture of intestinal-type (HGC-1, -2, and -4) cells. In culture of diffuse- and mixed-type (HGC-3, − 18, and − 20) cells, the ratio was significantly increased only in HGC-20 cells. Mean ± SD of five independent experiments. *, P < 0.05; **, P < 0.01 by Student’s t-test between before and after cultivation. Figure S5. HGC-3, -18, and -20 PDX cells, and MKN45 and NUGC-4 cells were treated with various concentrations of 5-fluorouracil (5-FU), doxorubicin and doxifluridine in REBM-based medium, and cell numbers were determined on day 14 by MTT assay. Mean ± SD of five independent experiments. *, P < 0.05; **, P < 0.01 by Student’s t-test compared with MKN45 cells (pink asterisks), and with NUGC-4 cells (blue asterisks) at corresponding drug concentrations. Figure S6. Phase contrast micrographs of HGC-3 and -20 cells in Matrigel, on day 14 and on day 19, respectively, in culture. Figure S7. Identification of drugs that suppress the growth of HGC-20 cells in primary culture. Figure S8. HGC-20 cells were seeded on day 0, drugs were added on day 1, and cell numbers were determined by MTT assay on day 5. If a drug completely suppresses the cell growth without toxic effect, the cell number on day 5 [file 13046_2019_1121_MOESM1_ESM.pdf]

Figure S1

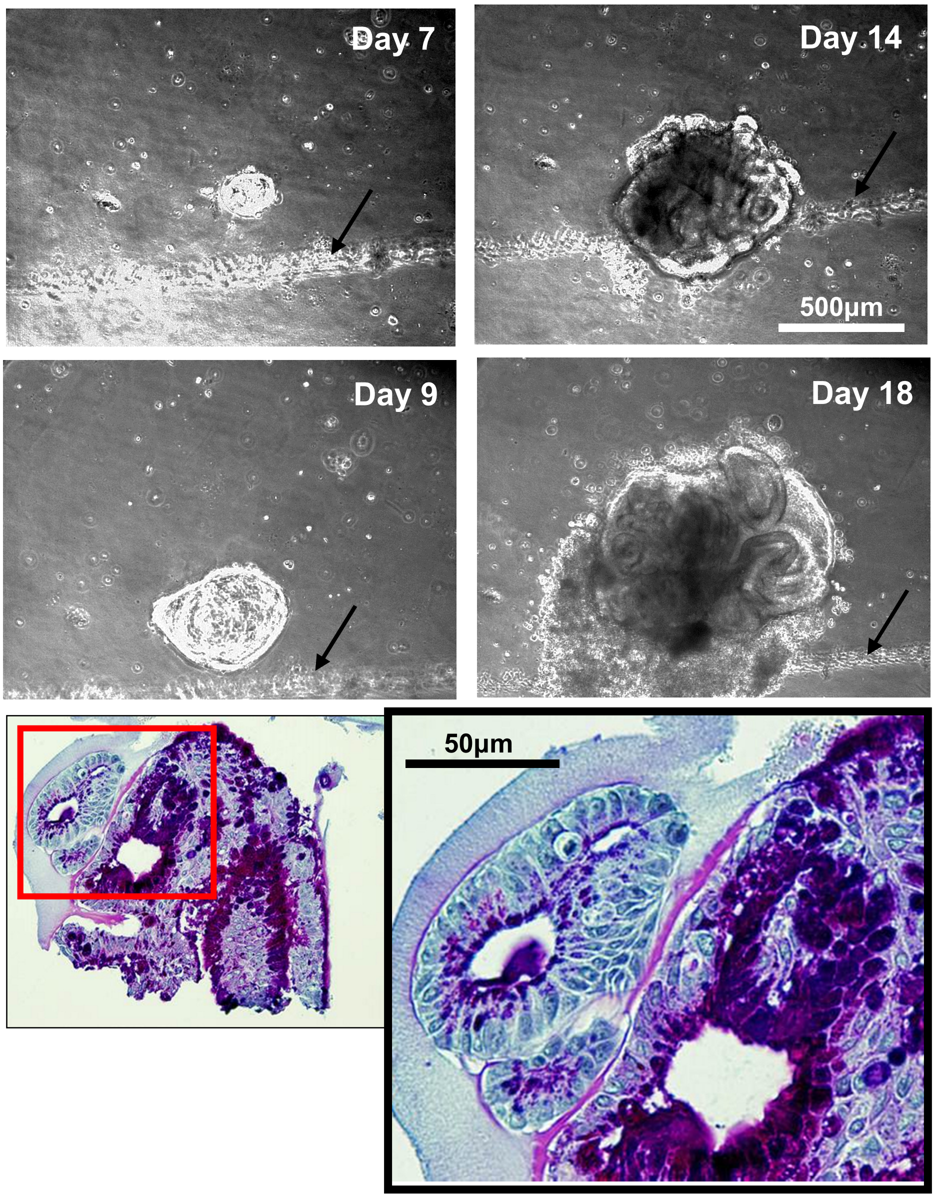

Figure S2

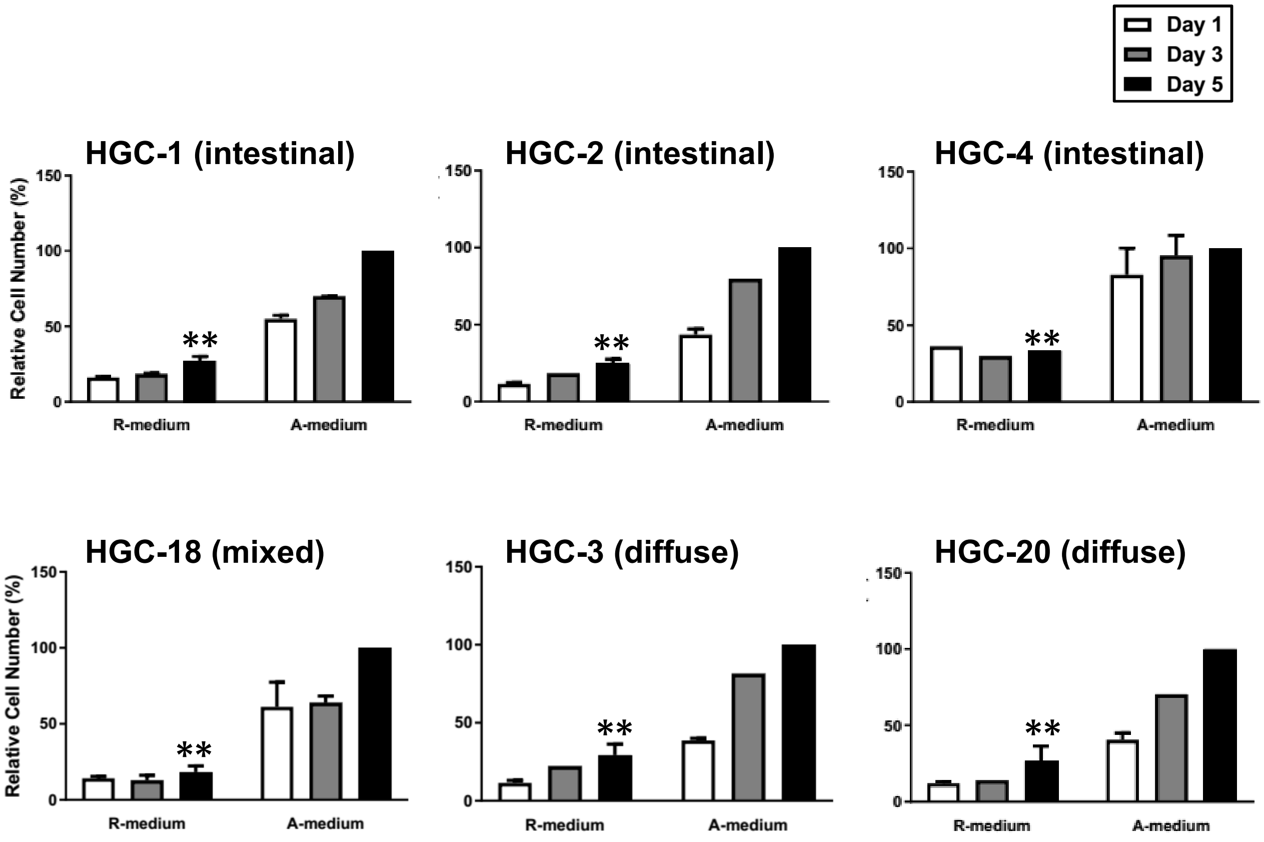
Day 1
Day 3
Day 5
HGC-1 (intestinal)
HGC-2 (intestinal)
HGC-4 (intestinal)
HGC-18 (mixed)
HGC-3 (diffuse)
HGC-20 (diffuse)
Relative Cell Number (%)
150
100
50
0
**
R-medium
A-medium

Figure S3

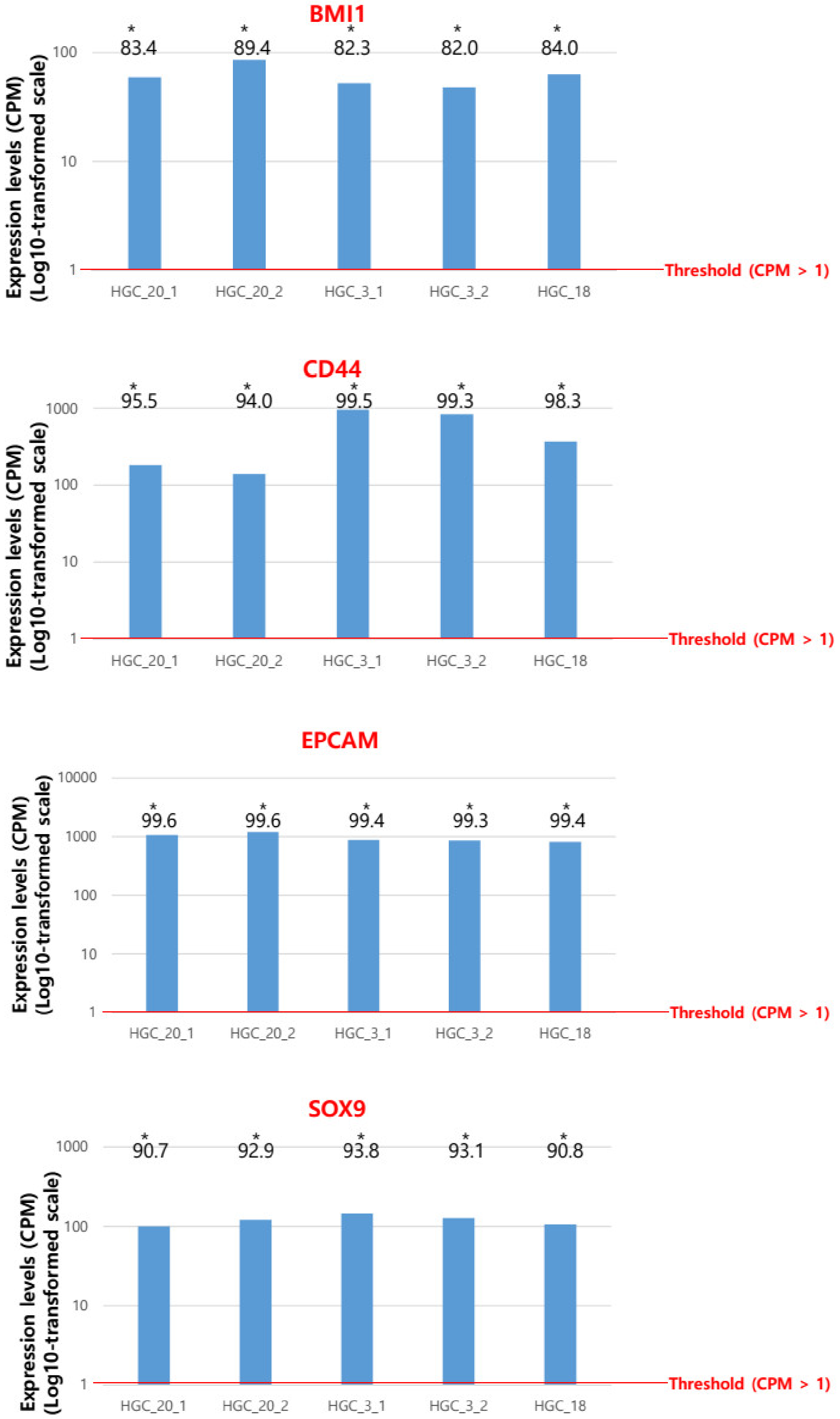
BMI1
Expression levels (CPM)
(Log10-transformed scale)
100
10
1
*
83.4
89.4
82.3
82.0
84.0
HGC_20_1
HGC_20_2
HGC_3_1
HGC_3_2
HGC_18
Threshold (CPM > 1)
CD44
Expression levels (CPM)
(Log10-transformed scale)
1000
100
10
1
95.5
94.0
99.5
99.3
98.3
HGC_20_1
HGC_20_2
HGC_3_1
HGC_3_2
HGC_18
Threshold (CPM > 1)
EPCAM
Expression levels (CPM)
(Log10-transformed scale)
10000
1000
100
10
1
99.6
99.6
99.4
99.3
99.4
HGC_20_1
HGC_20_2
HGC_3_1
HGC_3_2
HGC_18
Threshold (CPM > 1)
SOX9
Expression levels (CPM)
(Log10-transformed scale)
1000
100
10
1
90.7
92.9
93.8
93.1
90.8
HGC_20_1
HGC_20_2
HGC_3_1
HGC_3_2
HGC_18
Threshold (CPM > 1)

Figure S4

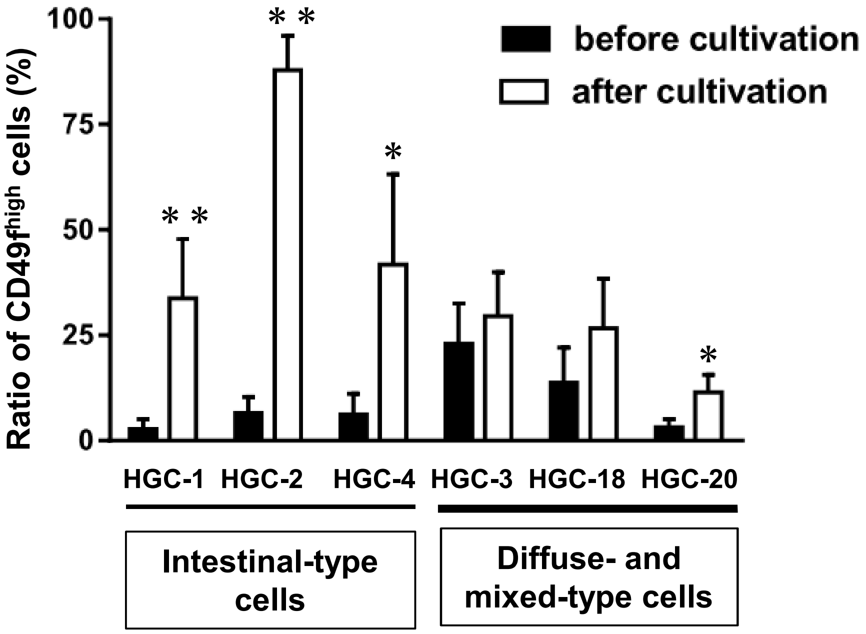

Ratio of CD49f^high cells (%)
100
75
50
25
0
* *
* *
*
*
before cultivation
after cultivation
HGC-1
HGC-2
HGC-4
HGC-3
HGC-18
HGC-20
Intestinal-type cells
Diffuse- and mixed-type cells

Figure S5

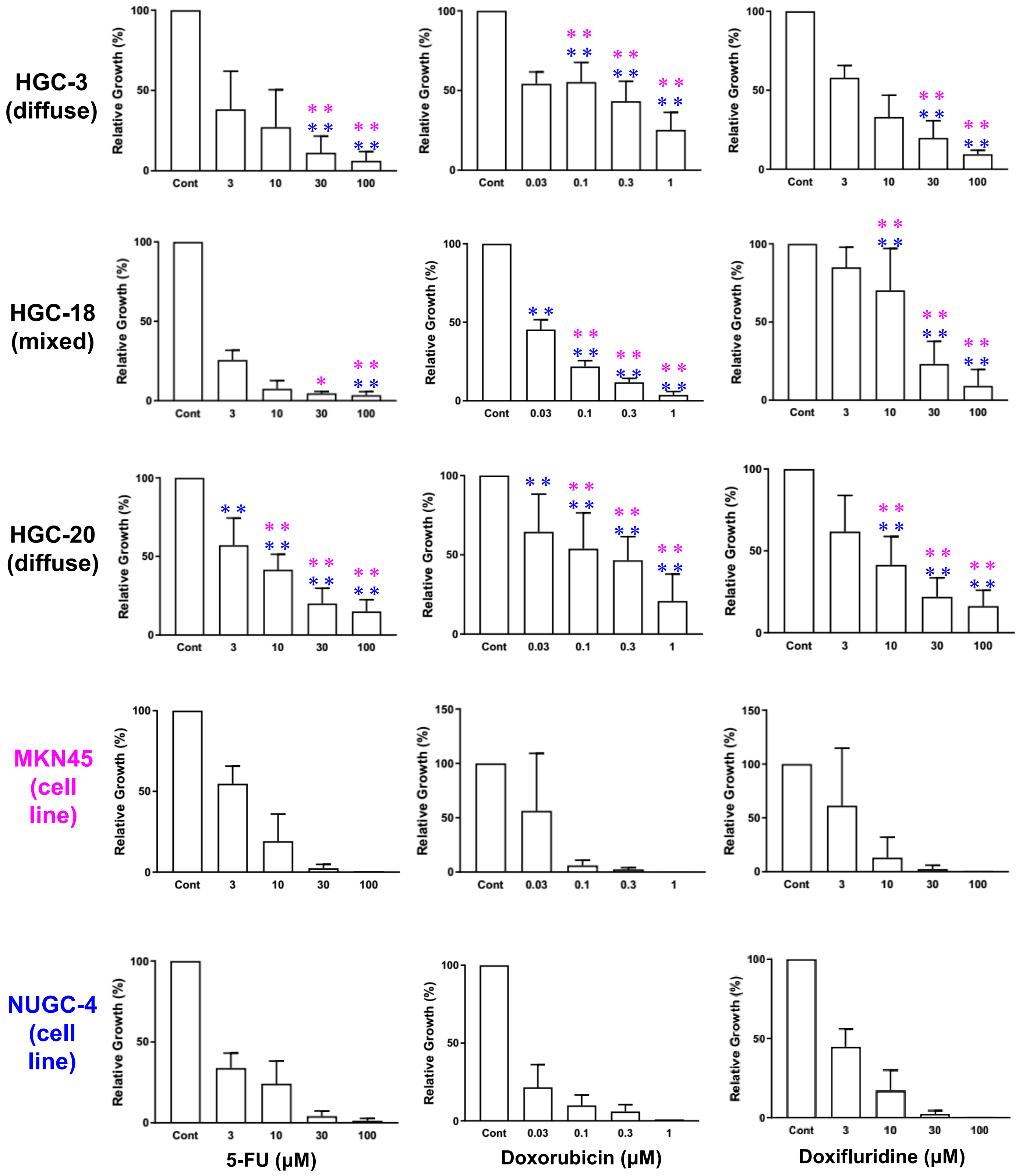
HGC-3
(diffuse)
HGC-18
(mixed)
HGC-20
(diffuse)
MKN45
(cell
line)
NUGC-4
(cell
line)
Relative Growth (%)
Cont
3
10
30
100
0.03
0.1
0.3
1
5-FU (μM)
Doxorubicin (μM)
Doxifluridine (μM)

Figure S6

HGC-3 cells on day 14

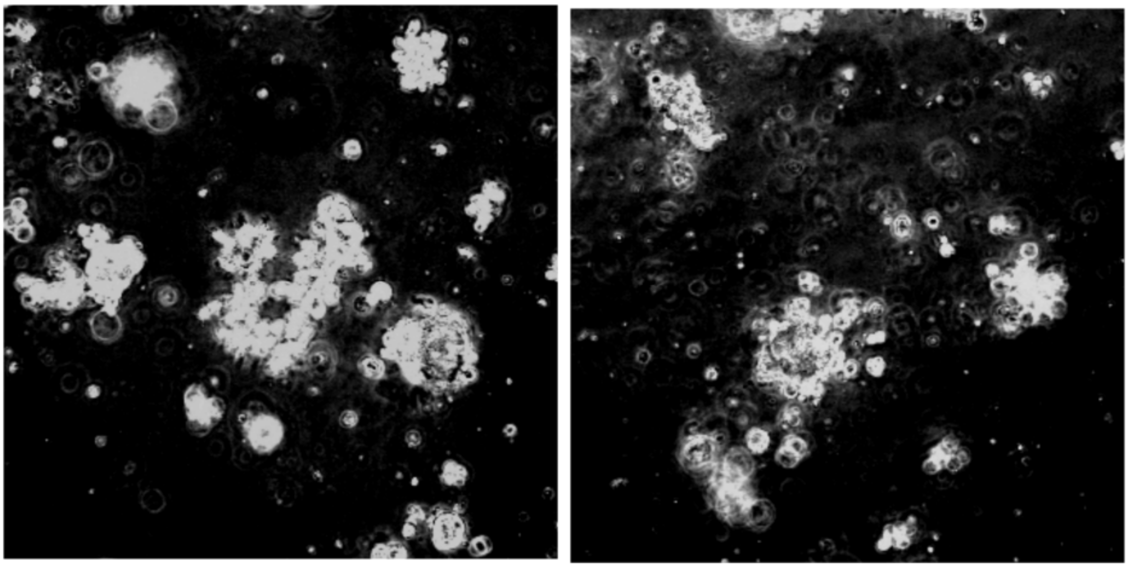

HGC-20 cells on day 19

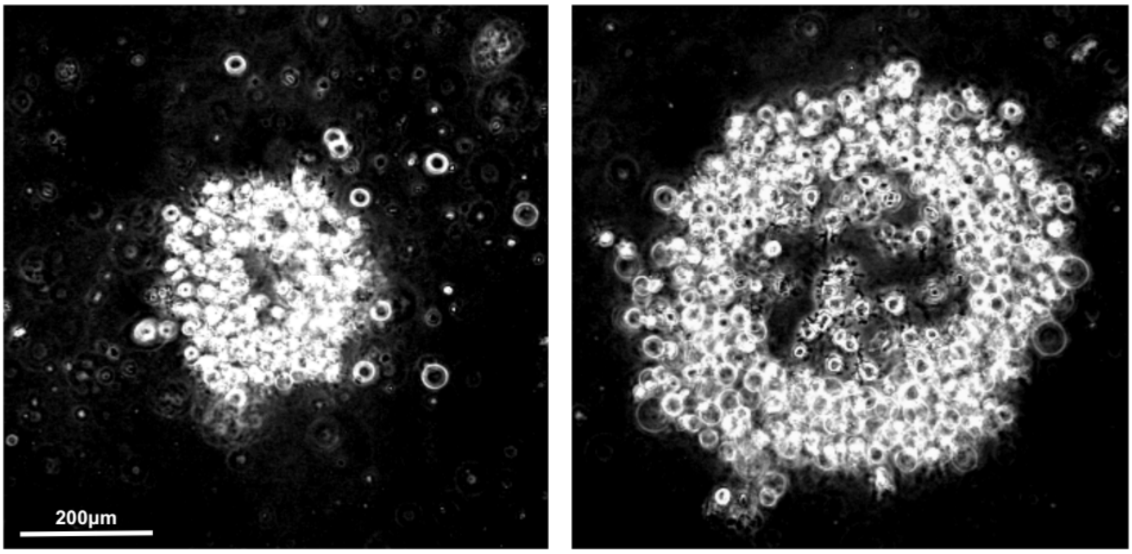

Figure S7

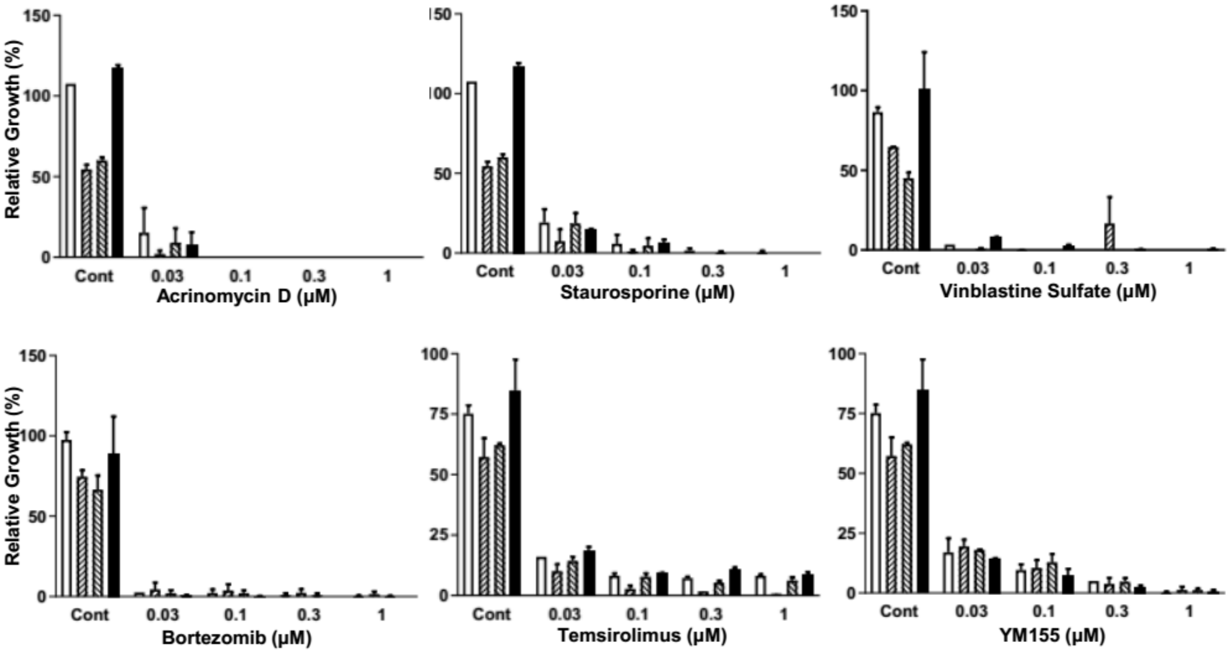

Relative Growth (%)
150
100
50
0
Cont
0.03
0.1
0.3
1
Acrinomycin D (μM)
Staurosporine (μM)
Vinblastine Sulfate (μM)
Bortezomib (μM)
Temsirolimus (μM)
YM155 (μM)
75
25

Figure S8

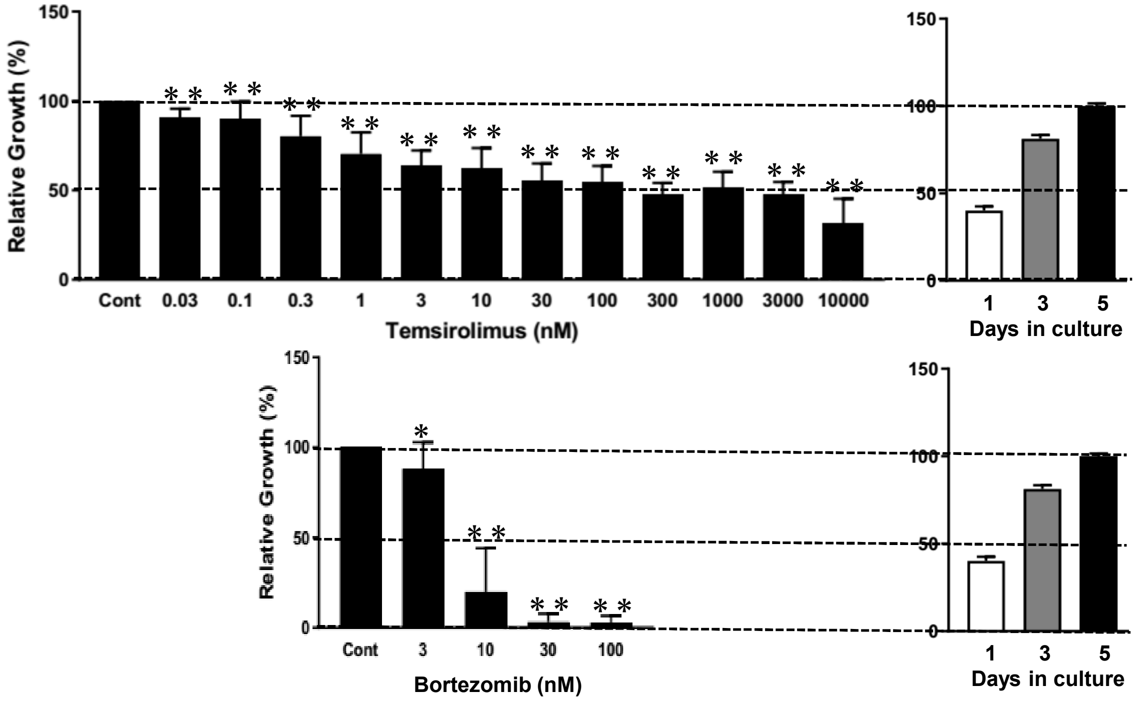
Relative Growth (%)
150
100
50
0
Cont
0.03
0.1
0.3
1
3
10
30
100
300
1000
3000
10000
Temsirolimus (nM)
1
3
5
Days in culture
Cont
3
10
30
100
Bortezomib (nM)

Figure S9

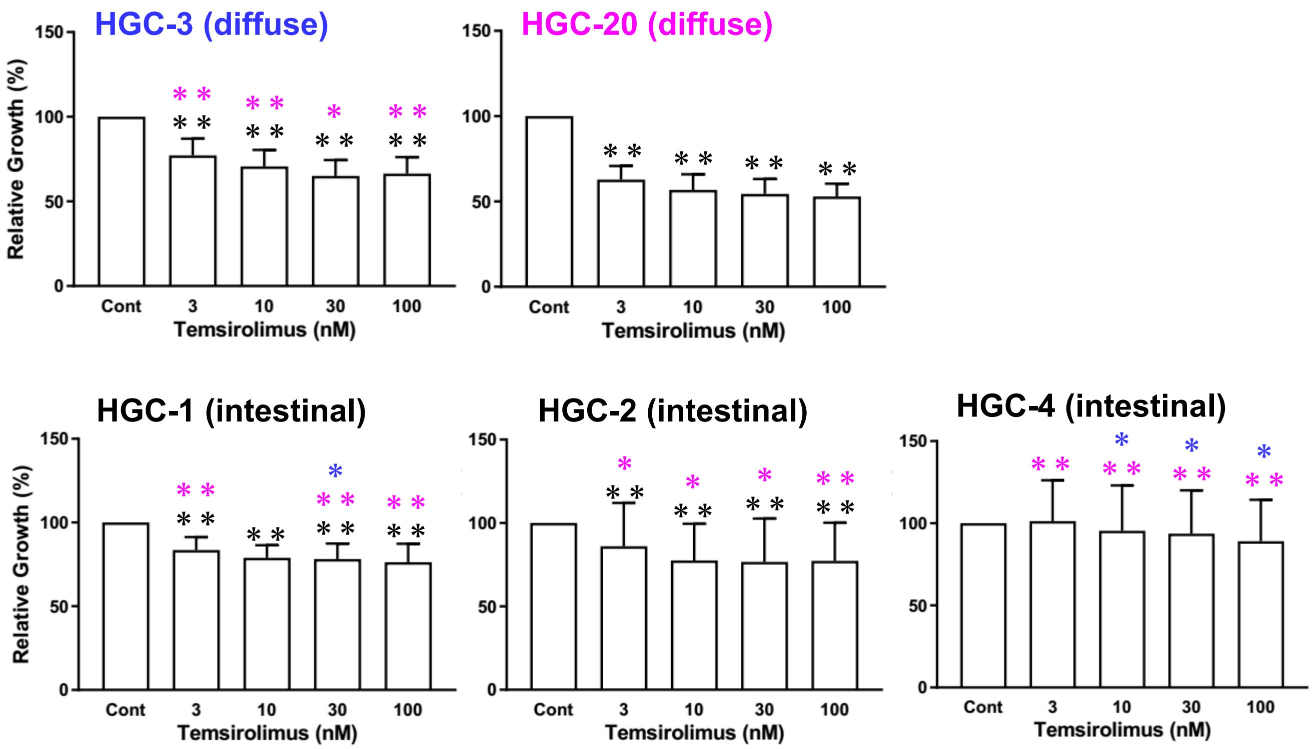
HGC-3 (diffuse)
HGC-20 (diffuse)
HGC-1 (intestinal)
HGC-2 (intestinal)
HGC-4 (intestinal)
Relative Growth (%)
150
100
50
0
Cont
3
10
30
100
Temsirolimus (nM)

Figure S10

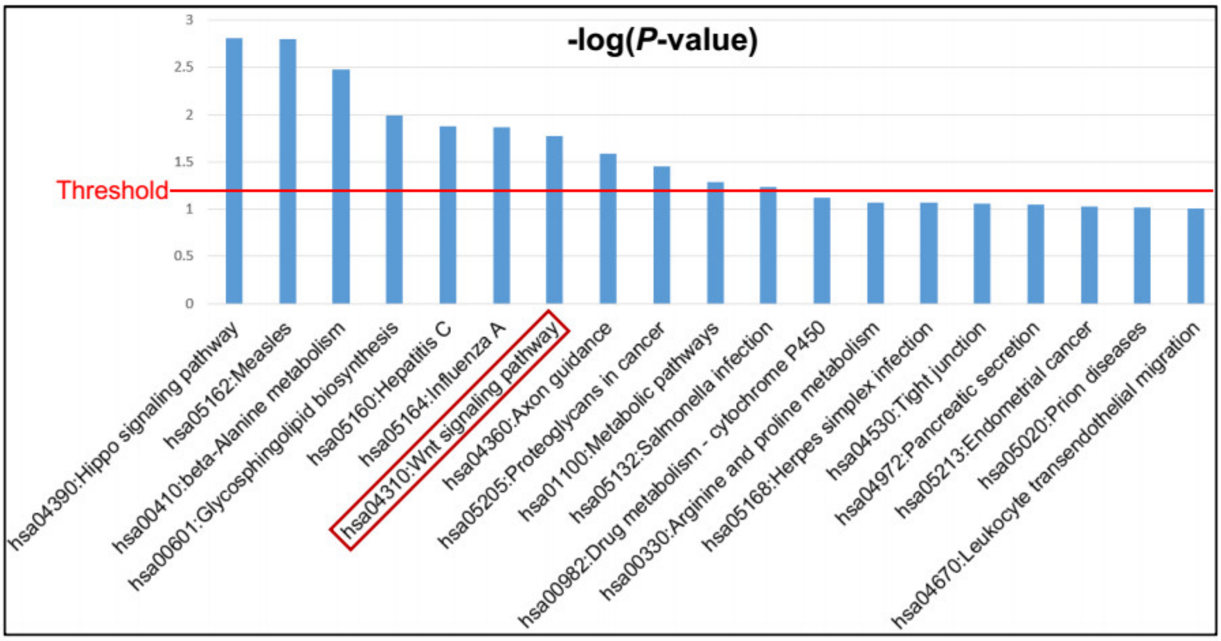

-log(P-value)
Threshold
3
2.5
2
1.5
1
0.5
0
hsa04390:Hippo signaling pathway
hsa05162:Measles
hsa00410:beta-Alanine metabolism
hsa00601:Glycosphingolipid biosynthesis
hsa05160:Hepatitis C
hsa05164:Influenza A
hsa04310:Wnt signaling pathway
hsa04360:Axon guidance
hsa05205:Proteoglycans in cancer
hsa01100:Metabolic pathways
hsa05132:Salmonella infection
hsa00982:Drug metabolism - cytochrome P450
hsa00330:Arginine and proline metabolism
hsa05168:Herpes simplex infection
hsa04530:Tight junction
hsa04972:Pancreatic secretion
hsa05213:Endometrial cancer
hsa05020:Prion diseases
hsa04670:Leukocyte transendothelial migration

Figure S11

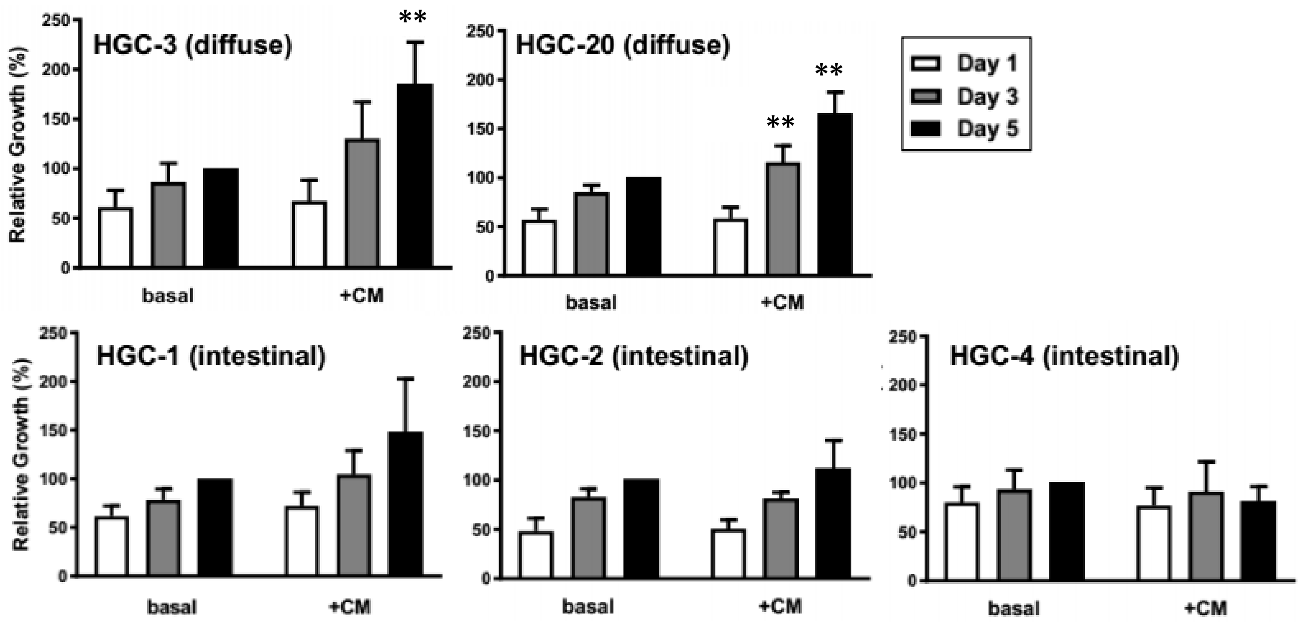

HGC-3 (diffuse)
HGC-20 (diffuse)
Day 1
Day 3
Day 5
HGC-1 (intestinal)
HGC-2 (intestinal)
HGC-4 (intestinal)
Relative Growth (%)
basal
+CM
**

Figure S12

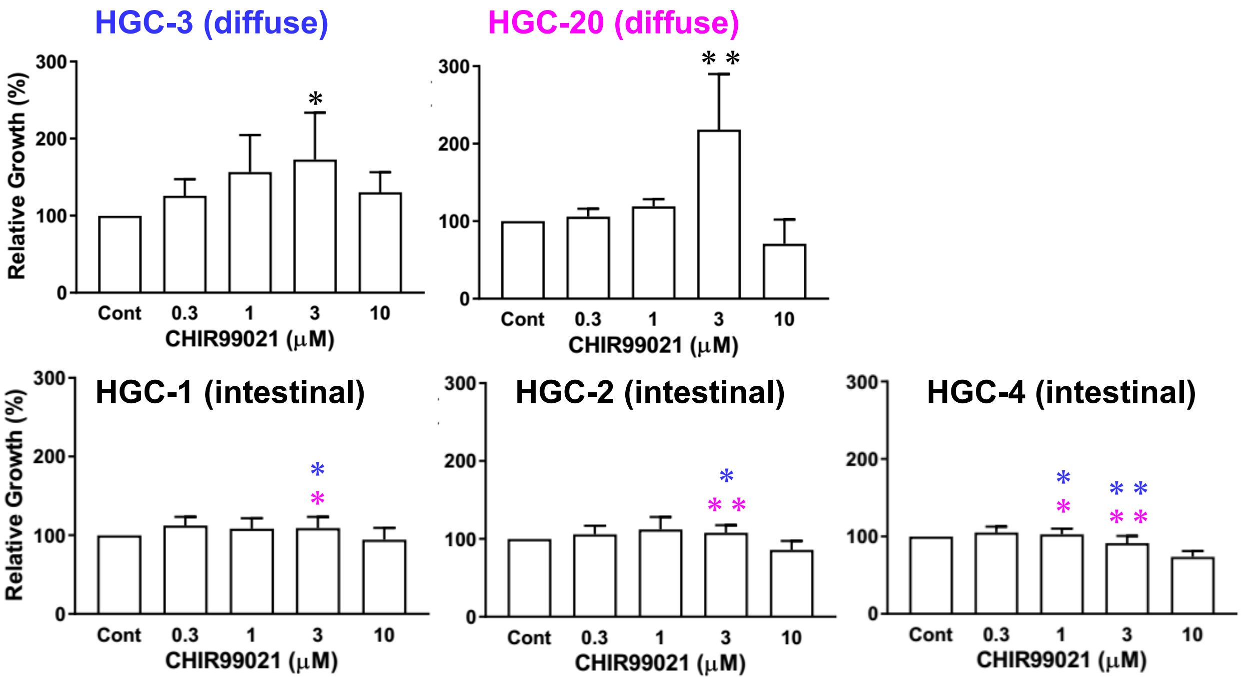

HGC-3 (diffuse)
HGC-20 (diffuse)
HGC-1 (intestinal)
HGC-2 (intestinal)
HGC-4 (intestinal)
Relative Growth (%)
300
200
100
0
*
* *
Cont
0.3
1
3
10
CHIR99021 (μM)

Figure S13

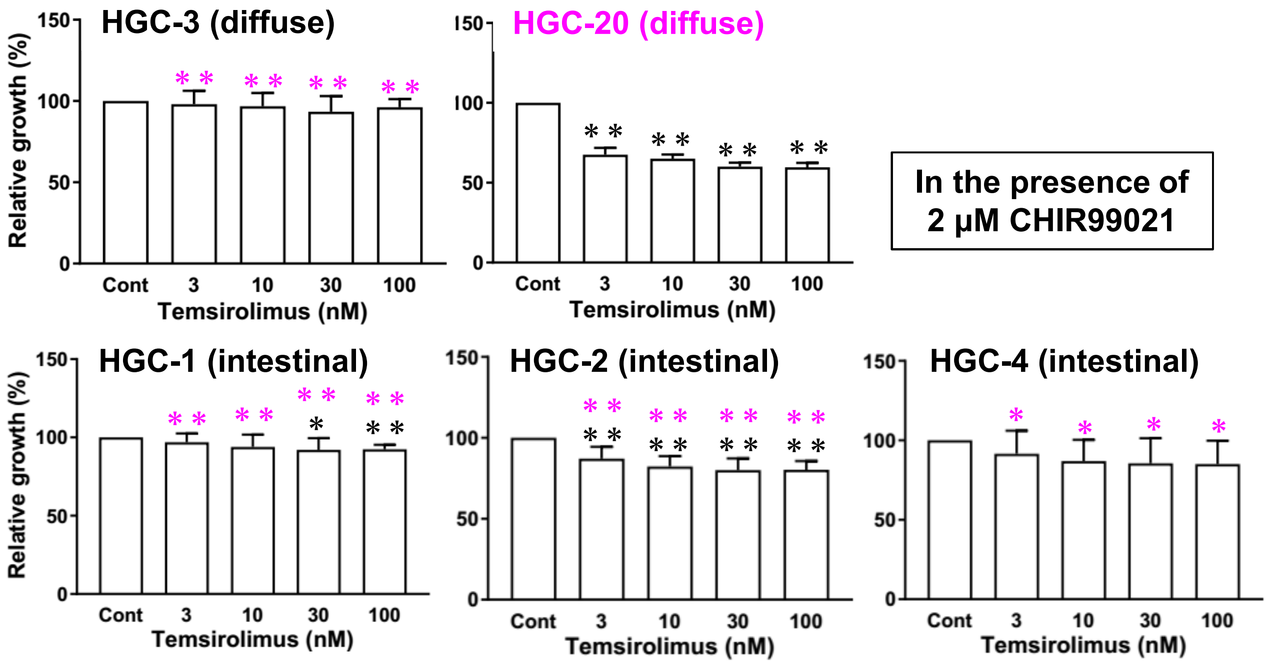
HGC-3 (diffuse)
HGC-20 (diffuse)
In the presence of
2 μM CHIR99021
HGC-1 (intestinal)
HGC-2 (intestinal)
HGC-4 (intestinal)
Relative growth (%)
150
100
50
0
Cont
3
10
30
100
Temsirolimus (nM)

Figure S14

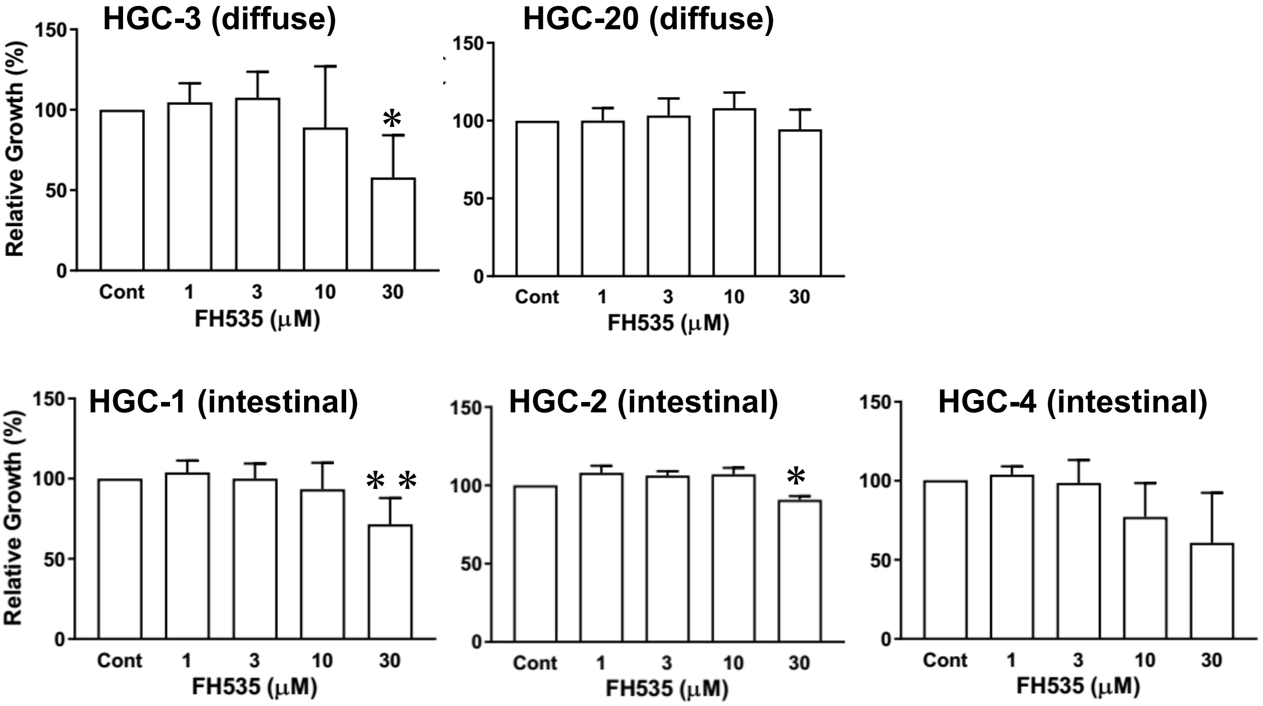
HGC-3 (diffuse)
HGC-20 (diffuse)
HGC-1 (intestinal)
HGC-2 (intestinal)
HGC-4 (intestinal)
Relative Growth (%)
150
100
50
0
*
**
Cont
1
3
10
30
FH535 (μM)

Figure S15

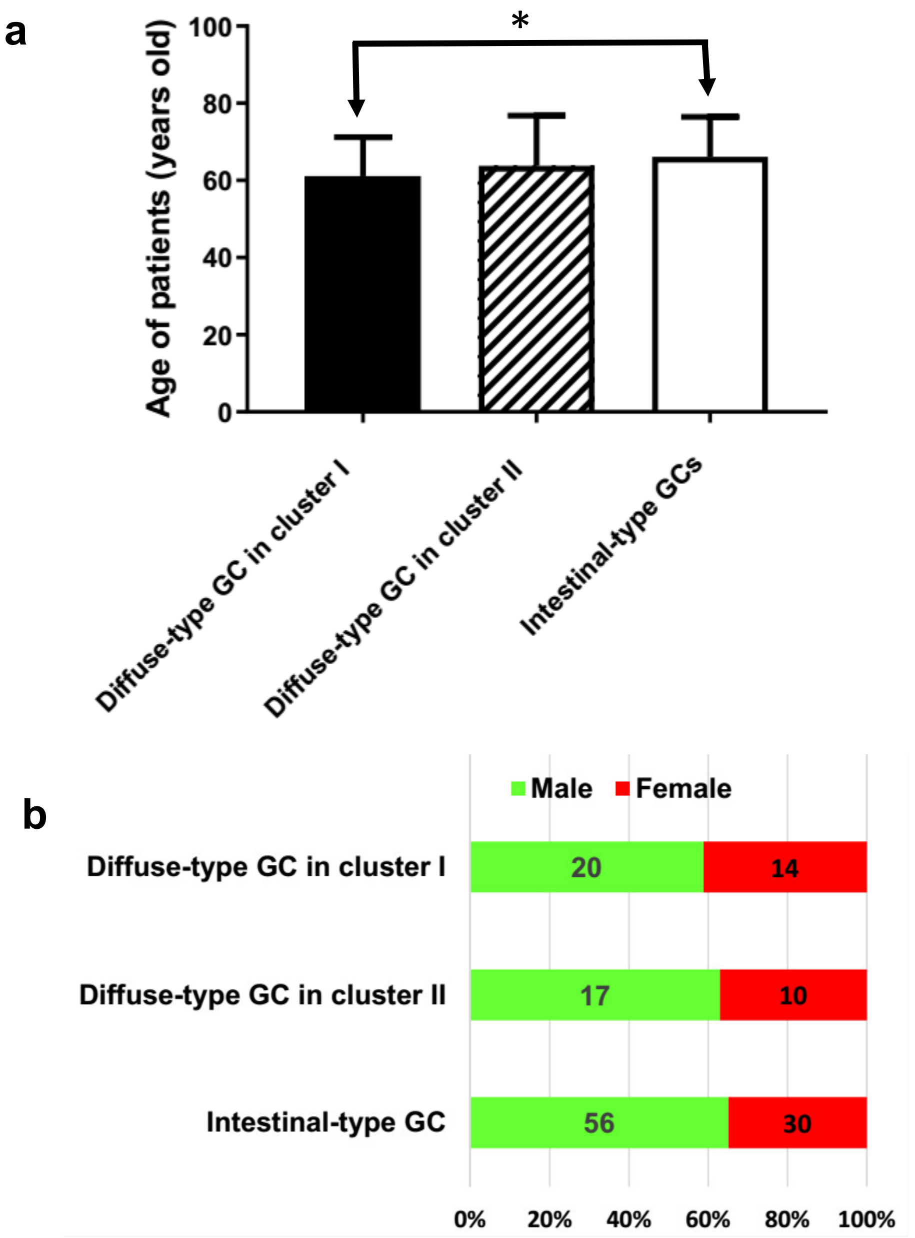
a
b
100
80
60
40
20
0
Age of patients (years old)
*
Diffuse-type GC in cluster I
Diffuse-type GC in cluster II
Intestinal-type GCs
Male
Female
Diffuse-type GC in cluster I
Diffuse-type GC in cluster II
Intestinal-type GC
20
14
17
10
56
30
0%
20%
40%
60%
80%
100%

Figure S16

a

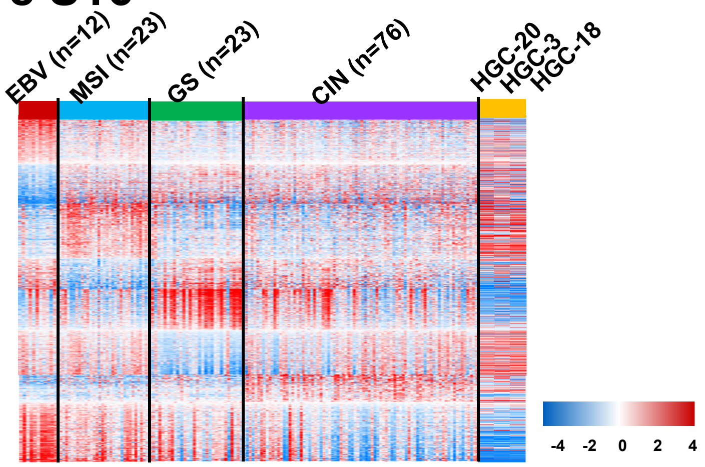

b

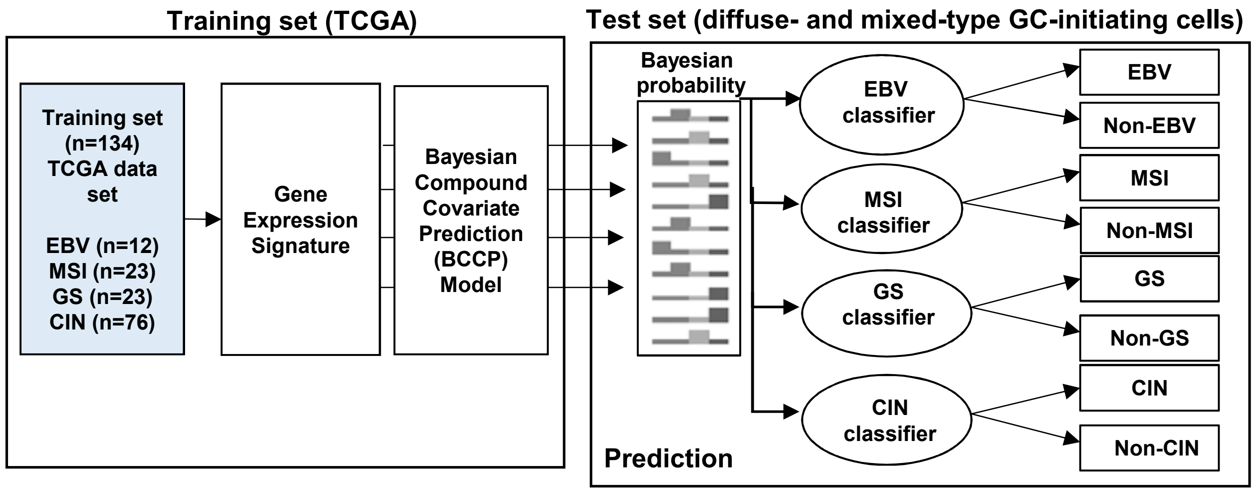

c

Performance of the BCCP:

Class	Sensitivity	Specificity	PPV	NPV
Non-EBV	0.984	0.917	0.992	0.846
EBV	0.917	0.984	0.846	0.992

Class	Sensitivity	Specificity	PPV	NPV
Non-MSI	0.838	0.739	0.939	0.486
MSI	0.739	0.838	0.486	0.939

Class	Sensitivity	Specificity	PPV	NPV
Non-GS	0.624	0.68	0.895	0.293
GS	0.68	0.624	0.293	0.895

Class	Sensitivity	Specificity	PPV	NPV
Non-CIN	0.433	0.473	0.4	0.507
CIN	0.473	0.433	0.507	0.4

d

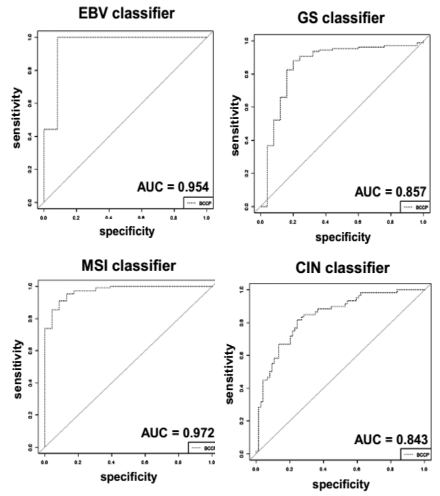

e

Array ID	EBV classifier	EBV	Non-EBV	MSI classifier	MSI	Non-MSI
HGC-3	Non-EBV	0.00%	100.00%	MSI	93.30%	6.70%
HGC-18	Non-EBV	0.00%	100.00%	MSI	100.00%	0.00%
HGC-20	Non-EBV	0.00%	100.00%	MSI	100.00%	0.00%

Array ID	GS classifier	GS	Non-GS	CIN classifier	CIN(%)	Non-CIN
HGC-3	Non-GS	0.90%	99.10%	CIN	94.70%	5.30%
HGC-18	Non-GS	0.80%	99.20%	CIN	90.70%	9.30%
HGC-20	Non-GS	0.80%	99.20%	CIN	91.00%	9.00%

Figure S17

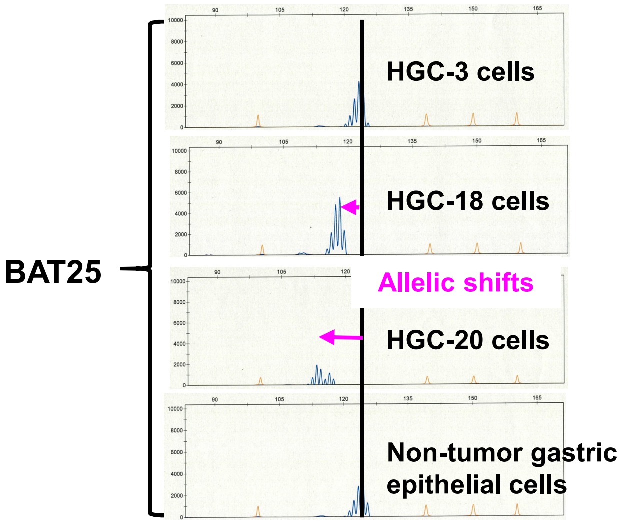
BAT25
HGC-3 cells
HGC-18 cells
Allelic shifts
HGC-20 cells
Non-tumor gastric epithelial cells

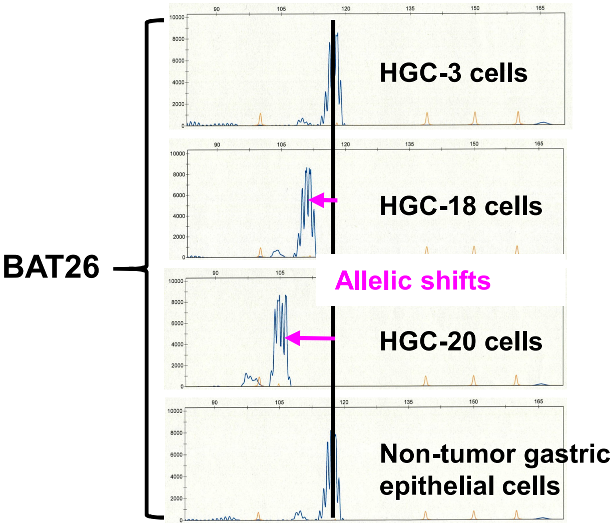
BAT26
HGC-3 cells
HGC-18 cells
Allelic shifts
HGC-20 cells
Non-tumor gastric epithelial cells

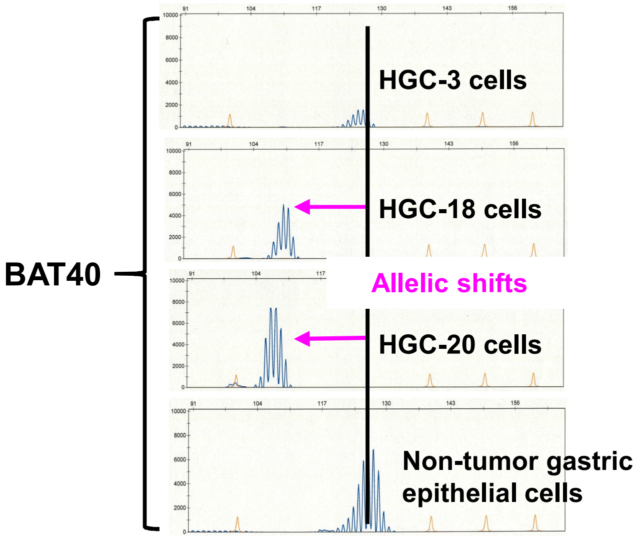
BAT40
HGC-3 cells
HGC-18 cells
Allelic shifts
HGC-20 cells
Non-tumor gastric epithelial cells

Figure S18

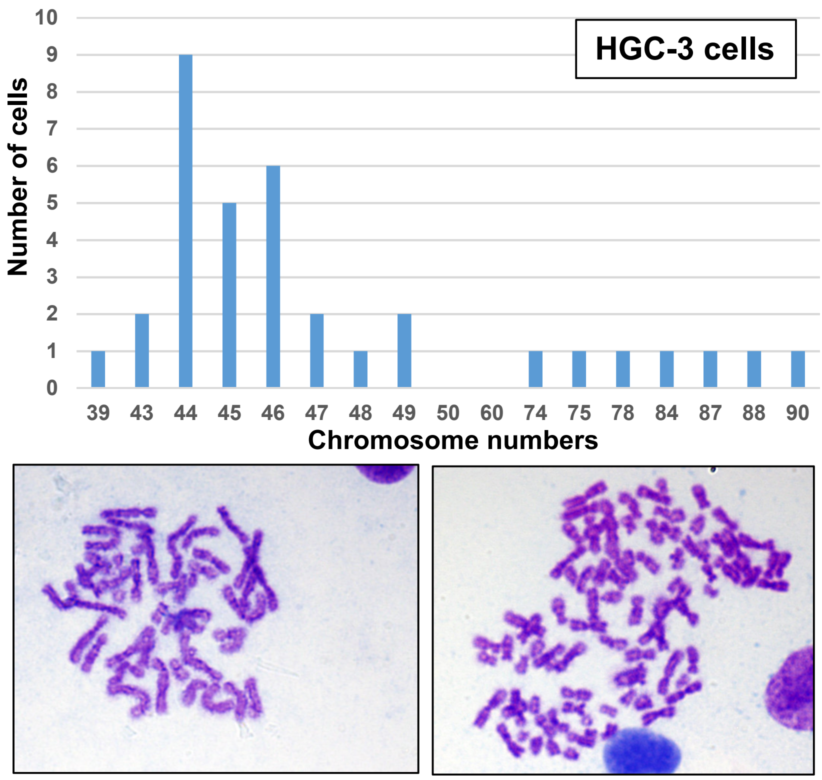

A cell with 49 chromosomes

A cell with 88 chromosomes

Figure S19

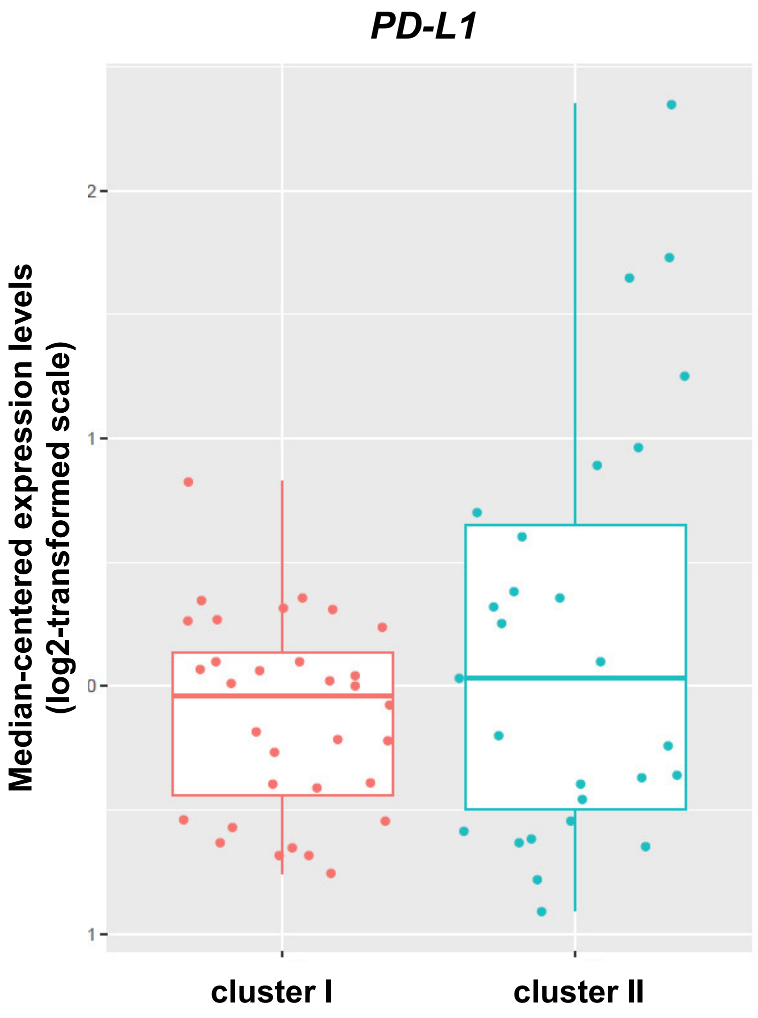

PD-L1
Median-centered expression levels
(log2-transformed scale)
2
1
0
1
cluster I
cluster II

Figure S20

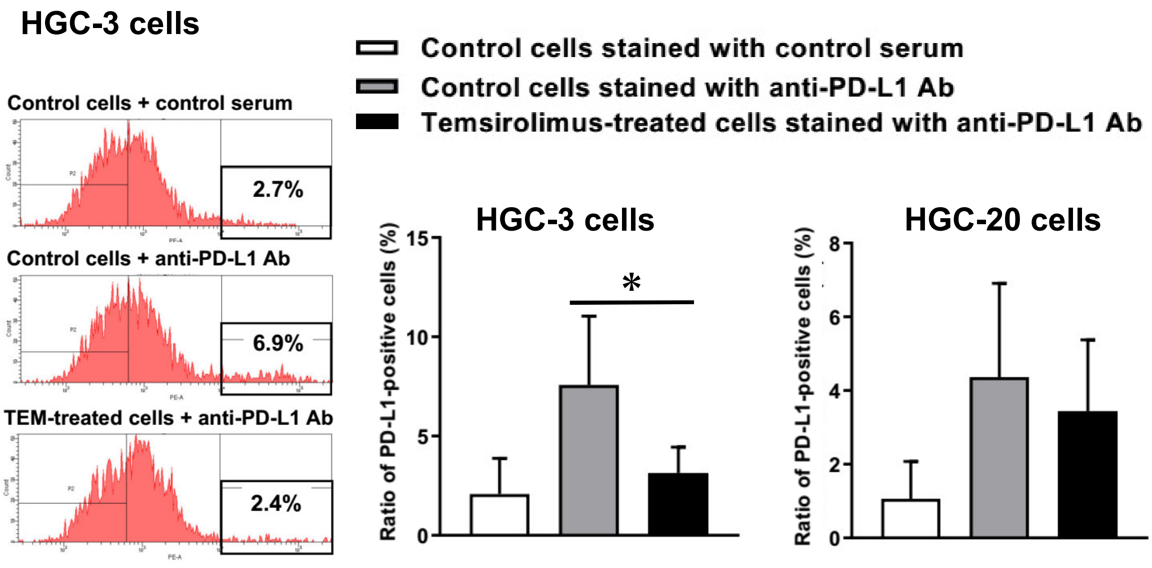

HGC-3 cells
Control cells + control serum
2.7%
Control cells + anti-PD-L1 Ab
6.9%
TEM-treated cells + anti-PD-L1 Ab
2.4%
Control cells stained with control serum
Control cells stained with anti-PD-L1 Ab
Temsirolimus-treated cells stained with anti-PD-L1 Ab
HGC-3 cells
Ratio of PD-L1-positive cells (%)
*
HGC-20 cells
Ratio of PD-L1-positive cells (%)

Figure S21

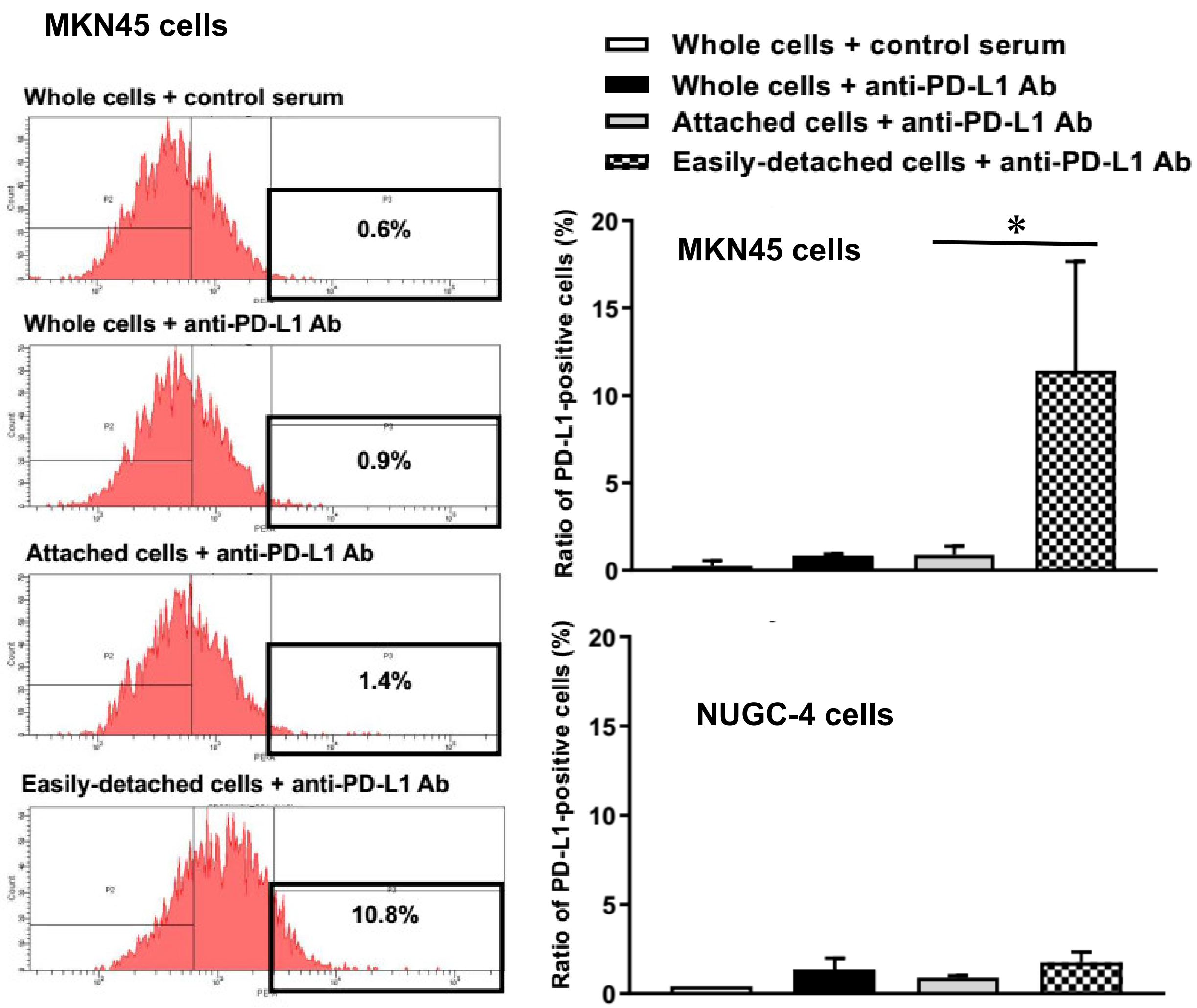
MKN45 cells
Whole cells + control serum
0.6%
Whole cells + anti-PD-L1 Ab
0.9%
Attached cells + anti-PD-L1 Ab
1.4%
Easily-detached cells + anti-PD-L1 Ab
10.8%
Whole cells + control serum
Whole cells + anti-PD-L1 Ab
Attached cells + anti-PD-L1 Ab
Easily-detached cells + anti-PD-L1 Ab
Ratio of PD-L1-positive cells (%)
MKN45 cells
*
20
15
10
5
0
Ratio of PD-L1-positive cells (%)
NUGC-4 cells
20
15
10
5
0

Figure S22

	Diffuse-type GC in Cluster I				Diffuse-type GC in Cluster II				Intestinal-type GC			
GC subtype	CIN	MSI	EBV	GS	CIN	MSI	EBV	GS	CIN	MSI	EBV	GS
Ratio of GC subtypes	35%	0%	13%	52%	47%	37%	16%	0%	65%	20%	5%	10%
Ratio of responders in subtypes	5%	100 %	100 %	12%	5%	100 %	100 %	12%	5%	100 %	100 %	12%
Estimated ratios of responders	1.75 %	0%	13%	6.24 %	2.35 %	37%	16%	0%	3.25 %	20%	5%	1.20 %
The ratio of responders	20.99%				55.35%				29.45%			
