# Supplementary material for: A subset of diffuse-type gastric cancer is susceptible to mTOR inhibitors and checkpoint inhibitors
Source: J Exp Clin Cancer Res. 2019 Mar 12;38:127. doi: 10.1186/s13046-019-1121-3 (PMC6416873; doi:10.1186/s13046-019-1121-3)
Supplement: Supplementary file 7 — Table S1. Clinicopathological features of diffuse-type GC patients whose cancers were used to establish PDX lines. Table S2. Differentially expressed 1048 genes in diffuse- and mixed-type GC-initiating cells compared with established cell lines. Table S3. Clinicopathological features of 610 GC cases used to prepare tissue array analysis. p-mTOR expression was significantly associated with venous invasion (P = 0.045) and perineural invasion (P = 0.011) in total cases, and perineural invasion in non-diffuse carcinomas (P = 0.027). P values were determined by Pearson’s chi-square test. The Spearman rank correlation analysis was used for pT and pTMN stages. Table S4. Univariate and multivariate survival analyses of diffuse-type GC (227 cases). p-mTOR was significantly associated with prognosis in univariate analysis, but it was not significantly associated with prognosis in multivariate analysis. P values were determined by Pearson’s chi-square test. The Spearman rank correlation analysis was used for pT and pTMN stages. Table S5. Univariate and multivariate survival analyses of total gastric carcinomas (610 cases). p-mTOR was not significantly associated with prognosis in univariate or multivariate analyses. P values were determined by Pearson’s chi-square test. The Spearman rank correlation analysis was used for pT and pTMN stages. Table S6. Candidate driver gene mutations and copy number variations in PDX cells. Please refer to https://www.ncbi.nlm.nih.gov/clinvar/variation/12582/ for pathogenic (#1), https://www.ncbi.nlm.nih.gov/clinvar/variation/24832/ for pathogenic (#2), https://www.ncbi.nlm.nih.gov/clinvar/variation/12580 for pathogenic (#3), and https://www.ncbi.nlm.nih.gov/clinvar/variation/39706/ for pathogenic (#4). (PDF 406 kb) [file 13046_2019_1121_MOESM2_ESM.pdf]

Table S1

	HGC-3	HGC-18	HGC-20
Operation day	2009-03-19	2014-01-06	2015-01-08
Age/ sex	72/ Male	82/ Male	65/ Female
Type	IV	II	II
Histology of GC	non-solid diffuse-type GC >> moderately-differentiated tubular GC	non-solid diffuse-type GC > moderately-differentiated tubular GC	non-solid diffuse-type GC >> signet-ring cell carcinoma > mucinous adenocarcinoma
pT stage	pT3	pT4a	pT3
Features of PDX	Signet ring cells	Mixed-type tumor	Fibrosis

Table S2

Symbol	P-value	FC.log2ratio
GALC	0.042023412	3.313746667
PRTFDC1	0.038408522	2.918793333
GSTT1	0.019555574	2.84854
VIM	0.003211191	2.479746667
PLA2G16	0.046972902	2.330033333
FSTL1	0.020851327	2.171106667
LOXL1	0.034722678	2.114306667
CXCL5	2.69377E-05	2.065513333
TGFBI	0.002336239	2.001926667
BASP1	0.049544934	1.955286667
CCDC28B	0.00598879	1.768213333
BCAT1	0.003685712	1.723353333
COL5A2	0.024544461	1.64028
UCHL1	0.008149773	1.634506667
CXCL2	0.017262182	1.63208
CCK	0.010012763	1.62282
MAP1B	0.012479239	1.548413333
PAQR8	0.015503815	1.503713333
FAM198B	0.002705931	1.483866667
EPHB2	0.007134805	1.475886667
GNG11	0.001205955	1.47234
SRPX	0.008427247	1.4545
CTHRC1	0.045084759	1.442386667
NUPR1	0.030439528	1.3649
FBP1	0.023375594	1.351233333
POPDC3	0.002027056	1.350233333
GSTA4	0.020623117	1.349073333
KLHDC8B	0.040824991	1.346686667
ALPP	0.045144262	1.32332
MIB2	0.043081991	1.315726667
TIMP3	0.032625438	1.28448
KRT20	0.006200654	1.270186667
DOCK4	0.001059124	1.26086
SLITRK6	0.011593183	1.240793333
HYI	0.019821739	1.22334
DKK3	0.008633634	1.218746667
UST	0.000518697	1.182233333
ZBTB20	0.005227438	1.165893333
FAM69A	0.01535305	1.165393333
MAGEH1	0.017611135	1.1329
CPE	0.014155573	1.122293333
CDC25B	0.032492825	1.113786667
SERPINI1	0.01899551	1.113126667
SEMA5A	0.007210242	1.112553333
ATL1	0.029828293	1.10888
FAM40B	0.003105431	1.0883
NPFFR2	3.00758E-05	1.076713333
ODZ2	3.36571E-06	1.0748
ZEB1	0.045137641	1.063233333
WNT5A	0.005268061	1.06306
BCHE	3.49871E-05	1.059186667
FGF20	1.33857E-06	1.050833333
CENPB	0.017909098	1.04988
CXCR7	0.045856731	1.048133333
WWC3	0.044604154	1.0472
TBCB	0.029195778	1.035526667
PDGFRA	0.001635387	1.034213333
LOC100505806	0.044489655	1.03288
SRGN	3.48239E-05	1.02576
TPTE	2.38856E-05	1.024233333
ZNF883	0.033006542	1.01928
TCF4	0.018756669	1.00824
IDH2	0.010460504	0.99098
GALNT2	0.000806294	0.976246667
CTAG2	6.24412E-07	0.9718
GPCPD1	0.028793958	0.969893333
SDC2	0.007611192	0.969493333
NID1	0.003759755	0.962193333
NUCB2	0.004484313	0.957746667
MCM5	0.024544167	0.951546667
FHOD3	0.018907474	0.949373333
ZSCAN18	2.40603E-06	0.946
FKBP1A	0.023191976	0.944906667
ENPP2	0.02243477	0.93454
C16orf95	0.012660368	0.929506667
ARFRP1	0.006759434	0.921386667
FSTL5	4.19406E-05	0.911673333
MAGEA10	0.000165451	0.906073333
HMCN1	0.005168188	0.892593333
NSFL1C	0.03742668	0.892453333
CDH2	0.017332575	0.887553333
NUCB1	0.010550119	0.884986667
GPC6	7.58431E-05	0.884466667
CABYR	0.013213512	0.880813333
NRTN	0.002752687	0.879853333
MRPS26	0.044034074	0.879753333
TNFRSF19	0.033318676	0.87812
GSTM4	0.003242594	0.877133333
HES6	0.038351677	0.875713333
MCAM	0.005213354	0.874646667
FKTN	0.027966908	0.872266667
DLX1	0.00178245	0.85864
SMC2	0.035288667	0.856453333
TMEM115	0.000791414	0.85316
BMP2K	0.006874582	0.85166
SNRNP25	0.017115176	0.847773333
HLA-DRA	0.003265153	0.840753333
KANK4	4.8708E-05	0.839013333
GLDC	0.001088526	0.83296
FAM176A	0.018102901	0.8298
LOC100507463	0.012184525	0.8254
MAMDC2	0.000138777	0.82402
ENOX1	0.000377158	0.814726667
SLIT2	0.000427888	0.81444
RBP1	0.031987886	0.814366667
KLHDC4	0.006558882	0.810606667
LRRC34	0.023182229	0.80826
PCDHB5	0.000114742	0.8059
DUS3L	0.034965612	0.805633333
PRICKLE2	0.023250862	0.803666667
TCFL5	0.028784406	0.803633333
SRD5A3	0.006997266	0.801353333
PTX3	0.027105404	0.795986667
ARMCX1	0.006673515	0.790593333
ZGPAT	0.018115594	0.78286
TMSB15A	0.033549896	0.778993333
HYAL2	0.047840803	0.778906667
CDH12	0.005066988	0.778086667
CYP24A1	0.000718325	0.76604
PPP3CB	0.036555686	0.760946667
ADAMTS1	0.000116687	0.76092
PEX2	0.018208379	0.759106667
LOC113230	0.002120188	0.748373333
C9orf169	0.005436085	0.742273333

Table S2 (continued)

**REG1A**	**0.033508247**	**0.73368**
**ZNF788**	0.000157061	0.725906667
**NPW**	0.000544154	0.72282
**NRM**	0.000123784	0.721133333
**LIPC**	0.010011387	0.714906667
**CDCA2**	0.036885751	0.71296
**KAL1**	0.021917026	0.71076
**SDPR**	0.007821103	0.707506667
**HLA-DPA1**	3.65286E-06	0.706833333
**MCM8**	0.004320703	0.70024
**PKIA**	0.000255034	0.69796
**NEXN**	0.045187054	0.695013333
**GHR**	0.005921763	0.694286667
**GHR**	0.005921763	0.694286667
**FLRT2**	0.005129279	0.694013333
**STAU2**	0.018851201	0.693933333
**TUBG2**	0.045822889	0.693893333
**EYA2**	0.033519547	0.692966667
**LOC255512**	0.037229405	0.68892
**CAND2**	0.005554175	0.684346667
**LOX**	0.028635722	0.682426667
**BEND6**	0.000171095	0.680653333
**CYBASC3**	0.047878576	0.67938
**ARMCX2**	0.00222177	0.677926667
**RUNX1-IT1**	0.00250879	0.670126667
**GLT8D2**	0.001708204	0.66708
**LOC728819**	0.03954345	0.663613333
**AGPAT5**	0.027728118	0.662373333
**POSTN**	0.009588919	0.661986667
**FGG**	0.023913764	0.658566667
**FILIP1L**	0.008324506	0.657966667
**PROX1**	0.038074883	0.64814
**C1QTNF6**	0.044953671	0.641206667
**PSMG4**	0.027643856	0.638913333
**ACSS3**	0.000721792	0.628613333
**WIF1**	0.012835748	0.626186667
**RGS4**	0.009625293	0.62208
**SPG7**	0.046213454	0.620833333
**PREP**	0.022793061	0.618873333
**APOA2**	7.02864E-08	0.6171
**RECK**	0.020356148	0.61638
**CDKAL1**	0.016851526	0.611106667
**ANKRD16**	0.047098279	0.60986
**LRCH2**	5.71127E-05	0.604206667
**CNOT7**	0.002762623	0.6041
**THAP8**	0.011512456	0.5991
**CTU2**	0.019136609	0.596993333
**BMP1**	0.011944651	0.59358
**C19orf60**	0.030773774	0.59108
**CCNB2**	0.00649617	0.58222
**CENPN**	0.042161971	0.5816
**SNORA64**	0.038998791	0.576106667
**SNORA64**	0.038998791	0.576106667
**COL6A3**	6.10402E-05	0.575993333
**SNCAIP**	0.031252268	0.57422
**LIN28B**	0.000787527	0.57388
**STEAP1B**	0.01335737	0.573493333
**SLBP**	0.02325326	0.572266667
**HIST1H2AM**	0.007030756	0.569786667
**HCP5**	0.024577712	0.5692
**LOC84856**	1.62175E-05	0.566933333
**PCDH18**	0.001366499	0.559826667
**SMARCB1**	0.011845636	0.55824

**CPA4**	**0.017051534**	**0.558226667**
**ZNF428**	0.005312217	0.557533333
**ASMTL**	0.027650511	0.557033333
**TELO2**	0.045825398	0.556506667
**CENPA**	0.049877314	0.55594
**LY96**	0.003548306	0.55424
**COX7B2**	2.04252E-06	0.553333333
**ITFG1**	0.004590799	0.55312
**E4F1**	0.009582729	0.552386667
**CLEC2B**	0.013280494	0.547073333
**MRPL15**	0.04214553	0.545706667
**FABP4**	0.000162742	0.5415
**PGP**	0.030449176	0.53942
**ZC3H18**	0.01389858	0.538426667
**GPR133**	0.006304732	0.535106667
**DOM3Z**	0.000296442	0.5345
**ZNF569**	0.004355244	0.53328
**HSD17B6**	0.026742268	0.53194
**C17orf101**	0.038983744	0.531213333
**FOXF1**	0.043881569	0.530766667
**PIGT**	0.046261447	0.530246667
**VEGFC**	0.003545544	0.529686667
**VAT1**	0.0435201	0.529086667
**PCSK1N**	0.044056464	0.527166667
**CXorf69**	0.005395246	0.523573333
**DEFA5**	0.001040148	0.522373333
**EDNRB**	0.00133898	0.52044
**PPP1R14A**	0.043800974	0.51956
**TSEN34**	0.006416615	0.517926667
**FOXL2**	0.021800203	0.517153333
**CAHM**	0.002124402	0.512986667
**HSD3B1**	0.006360125	0.509966667
**SYCE2**	0.040959402	0.50932
**IL7R**	0.022576525	0.50548
**RARRES2**	0.015027328	0.502753333
**PCP4**	5.83587E-05	0.501
**GCOM1**	0.021185156	0.500866667
**PBXIP1**	0.032969744	0.500253333
**GUCY1B3**	0.000223536	0.499433333
**FAM133A**	0.000753597	0.498133333
**EFHA2**	0.008277026	0.497586667
**PDCL**	0.049250407	0.497266667
**ZNF804A**	0.000227609	0.495066667
**VCAM1**	0.024732304	0.489493333
**POLR3K**	0.037901462	0.48802
**CHRDL1**	0.002947685	0.48778
**ATP8B2**	0.046933801	0.485426667
**LAYN**	0.003137406	0.4851
**NEUROD1**	0.02921373	0.482566667
**CUTA**	0.002521384	0.482166667
**NUP133**	0.018665693	0.48062
**FGF9**	0.035828936	0.4805
**BEX4**	0.001657418	0.4804
**DPY19L2**	0.005109021	0.480206667
**BEX1**	0.031543977	0.479046667
**GAS1**	0.01514569	0.478853333
**PCDH9**	0.000116248	0.477566667
**MAGEC1**	0.009967941	0.473273333
**KIFC1**	0.000323387	0.4669
**ZNF385B**	0.003671431	0.466166667
**TEX15**	0.028285602	0.465813333
**CADPS**	1.83412E-05	0.463266667
**NAP1L3**	2.2655E-05	0.4544

Table S2 (continued)

**FAM13C**	**0.026664385**	**0.450386667**
**ST6GALNAC3**	0.004218252	0.448113333
**ZNF879**	0.000231335	0.446
**FGB**	0.005467022	0.44584
**GABRB1**	6.26175E-05	0.4455
**MTTP**	0.008018975	0.442766667
**PLXDC2**	0.000150684	0.440333333
**CCR10**	0.004708908	0.43994
**SAGE1**	0.00115108	0.437233333
**DIO2**	0.011081924	0.43584
**C5orf55**	0.033371426	0.43336
**SSX1**	0.020756415	0.429366667
**WIPF1**	0.003193864	0.427053333
**SND1-IT1**	0.000374829	0.426773333
**CHMP1A**	0.046843096	0.42534
**NLGN1**	0.02664792	0.424173333
**DYNLRB2**	8.66245E-05	0.423166667
**FAM182B**	0.036630363	0.422786667
**RTN1**	0.04188502	0.422666667
**ZNF570**	0.004412991	0.420033333
**ZNF521**	0.001567908	0.41968
**LDOC1**	2.03216E-06	0.418466667
**S1PR3**	0.032802811	0.41836
**COL2A1**	0.001211781	0.416513333
**GPR143**	0.004444335	0.415573333
**LRRN3**	0.003388414	0.412873333
**FOLH1**	0.002003202	0.411773333
**MUC15**	2.72986E-05	0.4082
**PTN**	0.016341747	0.405166667
**LOC339535**	0.000116272	0.4045
**PAH**	0.001349898	0.400306667
**MYH4**	7.48372E-06	0.3972
**MBTPS1**	0.044333297	0.396706667
**RPL23AP32**	0.049184892	0.393833333
**KCNT2**	0.039092407	0.393353333
**PLCXD3**	0.001147791	0.393033333
**ITGBL1**	0.039650803	0.39278
**MAB21L1**	3.11707E-06	0.391433333
**KRBOX1**	0.008165683	0.388333333
**SMAD9**	0.032060183	0.387873333
**GPX7**	0.002164481	0.38734
**FAM26F**	0.03124122	0.38364
**LOC100128098**	0.024400816	0.3824
**LOC100130275**	0.03802962	0.378446667
**SYT11**	0.033957786	0.37838
**HS3ST3A1**	0.008058255	0.37674
**H2AFZ**	0.030495453	0.376446667
**ORM1**	0.003295577	0.376273333
**PCDHB18**	0.041785682	0.375926667
**GABRA2**	0.009839624	0.375666667
**ZFP82**	0.004663669	0.373633333
**MEOX2**	0.006640053	0.37212
**ELOVL4**	0.004123063	0.36998
**FAT3**	0.001131446	0.368893333
**LOC284408**	0.004619334	0.366733333
**PSG5**	0.000767926	0.366406667
**SMOC2**	3.37885E-06	0.365533333
**SCG3**	0.014492495	0.365053333
**PAGE1**	0.00037883	0.364833333
**UBE2I**	0.017721534	0.363413333
**SYT4**	1.29909E-06	0.363333333
**CLEC2L**	0.000176967	0.360833333
**MTSS1L**	0.014796695	0.36038

**MAPK7**	**0.04465493**	**0.35926**
**PCDHB7**	0.038478467	0.3581
**FLJ14107**	0.002898885	0.357553333
**HLA-E**	0.000594941	0.355366667
**SMEK3P**	0.002479424	0.354866667
**HBQ1**	0.017637256	0.3548
**RGS17**	0.006580395	0.35388
**PDZRN3**	0.035731142	0.353773333
**RBM20**	0.049777992	0.35244
**SLC10A4**	0.010012882	0.352146667
**FAM70A**	0.004326517	0.3517
**SLITRK5**	0.004163363	0.345906667
**DHRSX**	4.42961E-05	0.338133333
**RGS7**	5.58121E-06	0.337266667
**RNF217**	0.040317973	0.335513333
**SMPX**	0.03292314	0.33428
**ZNF542**	0.025794844	0.333673333
**ADAMTS3**	0.011494442	0.332953333
**GALNTL1**	0.02887451	0.332953333
**PAQR9**	0.046929846	0.33186
**CPAMD8**	0.023509203	0.33072
**TF**	0.022172498	0.324346667
**ZFPM2**	0.030484408	0.324226667
**TNNT3**	0.006370933	0.3241
**NAALAD2**	0.005134029	0.323513333
**PAGE4**	0.006529702	0.322933333
**AOX1**	0.034196146	0.32014
**EYA1**	0.030183387	0.319313333
**TUBA3C**	0.015666075	0.317633333
**KIF1A**	0.015867181	0.312113333
**DAPL1**	0.031098144	0.309533333
**BGN**	0.032212589	0.307453333
**GRIK2**	0.03057124	0.304853333
**NLGN4X**	0.001103153	0.303966667
**SH3GL3**	0.046931774	0.30386
**C1orf114**	0.013438091	0.300666667
**CDH19**	0.007294882	0.300373333
**EGFLAM**	0.015610709	0.296066667
**ISX**	0.000414859	0.294966667
**KLK5**	0.002723057	0.294806667
**NDNF**	0.02684967	0.294473333
**HLA-DMB**	0.000487729	0.293966667
**EDNRA**	0.000193559	0.292966667
**SLC6A15**	0.0007923	0.2922
**C10orf82**	0.043515209	0.292153333
**ESM1**	0.004463947	0.291833333
**DLGAP1**	0.007028194	0.291446667
**GAGE1**	4.0463E-05	0.290333333
**CHI3L1**	0.001205342	0.290133333
**INA**	0.009807406	0.28854
**ST8SIA1**	0.012016937	0.288486667
**JAKMIP2**	0.008673188	0.288333333
**CDH18**	0.043018918	0.2855
**PNMAL1**	0.004296169	0.284973333
**FGF3**	0.011307101	0.2833
**DOCK10**	0.038858641	0.282953333
**LOC100507410**	0.008893252	0.280173333
**PRR16**	0.000450232	0.279433333
**MGC16121**	0.003403103	0.276766667
**LINC00478**	0.000326784	0.2761
**MUC7**	0.039875957	0.275773333
**APCS**	0.040101066	0.2748
**SYNPR**	8.59965E-06	0.273333333

Table S2 (continued)

**NEGR1**	**0.047034842**	**0.271286667**
CNRIP1	0.006788729	0.270393333
FOXR2	0.003889672	0.2696
CRISP3	0.01627999	0.26784
DLL3	0.049611209	0.267293333
TTC29	0.014754065	0.264713333
KERA	0.006897222	0.2626
TRPC6	0.023237476	0.262586667
C3AR1	0.019060752	0.261866667
KCNK12	0.028295781	0.25546
CTNNA2	0.017690385	0.254366667
LGSN	0.045031137	0.248966667
HSD11B1	0.014295097	0.248593333
NCAM2	0.001266406	0.2448
PABPC4L	0.000773453	0.243633333
RALYL	0.009988909	0.242266667
PRRT1	0.024495638	0.240766667
SUSD5	0.032764324	0.240533333
ANKFN1	0.032632445	0.238646667
BLID	0.044857903	0.2373
FAM167B	0.01193584	0.236226667
PCP2	0.040924237	0.235326667
NEFH	0.027636224	0.233586667
NOL4	0.011226088	0.233506667
LCN12	0.041173953	0.233006667
KCNB1	7.40308E-05	0.230933333
CYP7B1	0.012802309	0.230713333
LOC389332	0.005622732	0.229233333
C20orf197	0.008850054	0.228606667
KIRREL2	0.010738687	0.228133333
DMRT1	0.026725856	0.227873333
KCNK2	0.008849831	0.2273
C1orf105	0.010553555	0.224966667
C21orf37	0.024873449	0.224766667
C9orf135	0.015071187	0.221733333
PPP1R16B	0.025406864	0.22102
LINC00173	0.041662878	0.21604
MYL7	0.006187441	0.2157
LINC00226	0.004009006	0.214666667
BHMT2	0.001262615	0.214
SLC27A6	0.024246609	0.2123
SCGB1D2	0.017628247	0.2119
ADAMTS5	0.028165313	0.210146667
COL21A1	0.000140362	0.210033333
NEUROG1	3.27816E-05	0.209566667
PHACTR1	0.031118744	0.208993333
C11orf88	0.008256741	0.208066667
ASIP	0.022193597	0.206913333
MAG	0.031123401	0.200833333
GPR88	5.98881E-05	0.198766667
AADACL2	0.038389384	0.1985
BRDT	0.000128218	0.196466667
FAM162B	0.006571556	0.1959
ZNF853	0.038874331	0.194793333
GABRA1	0.009747967	0.194033333
NTM	0.020383347	0.193266667
FEV	0.04734967	0.192466667
PPP1R3A	0.000433422	0.192366667
KLHL14	0.000660628	0.191166667
CYSLTR1	0.010982747	0.189966667
PF4V1	0.012470907	0.188126667
SPG20OS	0.002792813	0.186333333
RUNX1T1	0.037227679	0.186173333

**SPG20**	**0.010059364**	**0.1837**
MS4A7	0.000139851	0.183266667
NBLA00301	0.006859134	0.182633333
ZFP28	0.024942229	0.181533333
PRNT	0.012369505	0.181133333
EVC2	0.008584067	0.180366667
DNM3OS	0.030516743	0.179813333
HAPLN1	0.015159598	0.178166667
RNF175	0.045708829	0.177926667
DLK1	0.036608751	0.177006667
KRT40	0.001190156	0.175566667
SLITRK1	0.001850698	0.174366667
BNC1	0.047616629	0.173766667
FAM26E	0.036200692	0.173326667
CDH5	0.004420931	0.173
GRAP2	0.024903603	0.1729
HAND2	0.001622466	0.170066667
HP	1.08331E-05	0.169166667
NELL1	0.037882815	0.1668
CD7	0.003655935	0.164566667
CYP4F8	0.041855432	0.16416
C14orf23	0.005402646	0.160766667
LOC389023	0.018578869	0.158433333
RPRML	0.009275136	0.158233333
SHISA6	0.000781341	0.1578
TNFRSF13B	0.041892805	0.156466667
RSPO1	9.16862E-05	0.155266667
ASXL3	0.037523817	0.154533333
FAM170B	0.028778638	0.1541
GZMH	0.002872705	0.1535
SERPINI2	0.000125194	0.152266667
ZNF568	0.035185462	0.151533333
HMX1	0.017652816	0.148433333
PEG3	0.041911145	0.148266667
LIX1	0.004255303	0.1472
PRAC	0.002130664	0.146933333
PTGFR	0.005281698	0.144733333
NHLH2	0.008483494	0.144566667
TBX5	0.010475997	0.1433
MARCH11	0.043345041	0.138233333
SLC18A1	0.002902665	0.1377
LOC285796	0.012873149	0.137633333
NDN	0.013957996	0.136366667
EPHA5	0.0007422	0.136133333
CRISP2	0.049516793	0.136
ZBTB22	0.008105907	0.1353
IL17B	0.044033551	0.134866667
POU3F2	0.026974178	0.1348
SCUBE1	0.000567756	0.134533333
CDRT15L2	0.037535936	0.133866667
FAM9C	0.0366268	0.132433333
C16orf54	0.003531818	0.127466667
MLN	0.002202983	0.1273
PROK2	0.010709865	0.1255
PAR5	0.000143084	0.125133333
NKX2-1	0.007282001	0.125066667
GPR15	0.011919661	0.124466667
STL	0.038402732	0.123733333
RP1-177G6.2	0.017081468	0.121433333
GRIA2	0.00918493	0.121333333
GPR45	0.047060688	0.118766667
POMC	0.013498154	0.1148
LOC286177	0.016494821	0.112966667

Table S2 (continued)

**LOC100192378**	**0.024199495**	**0.111066667**
**MNDA**	0.019606341	0.110766667
**LINC00320**	0.007863277	0.109733333
**TMEM35**	0.025809813	0.109733333
**TSPAN16**	0.014532315	0.109466667
**C5orf48**	0.042831445	0.106833333
**LGI1**	0.001109401	0.106566667
**BHLHE23**	0.007855826	0.104333333
**CNKSR2**	0.004352049	0.1024
**SLC6A11**	0.000688316	0.102266667
**PDLIM3**	0.033132606	0.099966667
**ASTN1**	0.007026096	0.0987
**HOXC12**	0.04600254	0.0966
**DPP10**	0.035517775	0.093733333
**KRTAP4-11**	0.025421197	0.092566667
**OR1Q1**	0.026479867	0.092466667
**PRSS54**	0.015972464	0.091233333
**ECSCR**	0.048235201	0.089866667
**LOC100505782**	0.020991372	0.089766667
**ABCA8**	0.017335593	0.089233333
**TNP2**	0.006811524	0.089
**LOC283392**	0.013992803	0.088733333
**WFDC11**	0.003701689	0.085666667
**LOC554201**	0.038489568	0.083566667
**KRT222**	0.02830291	0.082533333
**OR1F2P**	0.00123847	0.0824
**KRTAP9-3**	0.003045747	0.0823
**CCIN**	0.04994175	0.078366667
**SNORD114-3**	0.006944878	0.0776
**LOC100507433**	0.018253899	0.0775
**CXorf21**	0.016152425	0.0772
**KCNA4**	0.006613024	0.075933333
**PLN**	0.008564347	0.075066667
**CALCA**	0.010853791	0.0745
**CRYGC**	0.043503565	0.0692
**TBX20**	0.01378922	0.067333333
**ADAM33**	0.004509875	0.067233333
**ECRP**	0.041340472	0.063133333
**RNF212**	0.033172058	0.062766667
**VPS52**	0.029512171	0.062566667
**GNG8**	0.047188848	0.062566667
**C18orf42**	0.025237554	0.061333333
**PLA2G2E**	0.003612232	0.0613
**RMST**	0.024255634	0.06
**IQCF2**	0.032143306	0.059733333
**CWH43**	0.045379517	0.0595
**LOC254312**	0.006638706	0.059233333
**DAZL**	0.007583907	0.0584
**DMRTC2**	0.008120824	0.0578
**COL15A1**	0.032071289	0.055733333
**TPPP2**	0.033298282	0.054
**FCRLA**	0.045288901	0.048966667
**ADGB**	0.011635418	0.046766667
**EFCAB1**	0.040637318	0.044
**REG3A**	0.048799883	0.0333
**CCDC36**	0.029477379	-0.036166667
**SRY**	0.031678954	-0.0433
**MYH8**	0.028697612	-0.043733333
**LILRB2**	0.010969156	-0.045233333
**NPVF**	0.023357599	-0.046866667
**SLC5A7**	0.008823247	-0.0501
**DRD3**	0.006781241	-0.0545
**KRTAP4-3**	0.012854485	-0.0565

**OR2H1**	**0.045612891**	**-0.0607**
**LOC646268**	0.045378284	-0.0615
**NXPH1**	0.027326483	-0.063733333
**CYLC2**	0.033924425	-0.063766667
**KRT38**	0.038003197	-0.063866667
**KCNT1**	0.020640152	-0.064433333
**C19orf75**	0.035516759	-0.065733333
**LY86-AS1**	0.00337371	-0.065766667
**ZNF479**	0.011108125	-0.066233333
**HTR2A**	0.008111446	-0.0668
**GPR12**	0.035955957	-0.067166667
**LOC100507066**	0.011240951	-0.0701
**SPO11**	0.019820192	-0.070233333
**LOC100128176**	0.001917571	-0.071566667
**LOC401134**	0.009989425	-0.0731
**CSN1S1**	0.02287984	-0.0769
**GPR50**	0.007021642	-0.078733333
**OPN1SW**	0.012146443	-0.078733333
**ASIC5**	0.005598349	-0.081533333
**LTA**	0.005937752	-0.082066667
**MOG**	0.00398018	-0.082333333
**HTR3C**	0.013845107	-0.0828
**FFAR1**	0.020228778	-0.083666667
**C2orf71**	0.032970554	-0.084566667
**PMP2**	0.009415987	-0.0861
**MOS**	0.023162151	-0.086933333
**CYP11B1**	0.032014207	-0.088933333
**HIST1H2BA**	0.022810424	-0.0908
**PPP3R2**	0.048778719	-0.091
**NKG7**	0.04134967	-0.095466667
**SLC17A6**	0.003501842	-0.095966667
**SPHKAP**	0.001976753	-0.097466667
**SPANXA2-OT1**	0.032341014	-0.0977
**CES5A**	0.003224798	-0.099966667
**SERPINA7**	0.035592797	-0.100033333
**TAS2R7**	0.037021018	-0.1018
**NR0B1**	0.047975918	-0.1018
**LOC100506895**	0.001242781	-0.102333333
**CST11**	0.047612994	-0.102333333
**LOC100505545**	0.00711095	-0.103666667
**KIRREL3-AS3**	0.036402864	-0.103666667
**CCL18**	0.000603267	-0.106533333
**GUCA1C**	0.023701392	-0.1067
**ADARB2**	0.042730268	-0.106866667
**C9orf62**	0.001423636	-0.106966667
**ZSWIM2**	0.032859726	-0.109166667
**MGC2889**	0.038525452	-0.1099
**DKFZp451B082**	0.007601072	-0.11
**MS4A3**	0.009941842	-0.1109
**MAGEB4**	0.00076869	-0.114866667
**FAM19A1**	0.025128554	-0.115066667
**PSG11**	0.005706787	-0.1151
**LOC285401**	0.039774718	-0.1158
**SPATA3**	0.009037715	-0.117166667
**DEFB123**	0.037550942	-0.1182
**LRTM1**	0.000390682	-0.119166667
**TGM7**	0.005115422	-0.1201
**NPHP3-AS1**	0.023721206	-0.1202
**BPIFA1**	6.40121E-05	-0.120966667
**FGF14-IT1**	0.000401709	-0.121366667
**PDE6H**	0.012782644	-0.1245
**PRSS37**	0.011031492	-0.1246
**OR2B3**	0.037376738	-0.125

Table S2 (continued)

**LOC100144597**	**0.001549021**	**-0.125033333**
**ASB5**	0.036359136	-0.1252
**HTR1E**	0.00533986	-0.1257
**LOC283143**	0.038153364	-0.126366667
**DAOA**	0.000463421	-0.130566667
**IFNG**	7.09703E-05	-0.132933333
**C11orf94**	0.030363154	-0.132966667
**PRRG3**	0.001959014	-0.133166667
**PRDM9**	0.045131592	-0.13348
**MS4A6E**	0.013190936	-0.136233333
**LOC221122**	0.033046392	-0.14
**UROC1**	0.043153605	-0.140366667
**C20orf173**	0.021010937	-0.141166667
**MC4R**	0.021003153	-0.1412
**LINC00163**	0.019020538	-0.1425
**GABRG1**	0.029399219	-0.143666667
**KRT35**	0.025632664	-0.143766667
**SLC34A1**	0.028458187	-0.144666667
**KBTBD5**	0.020480898	-0.146066667
**LOC257358**	0.000359071	-0.1467
**OR5E1P**	0.024444635	-0.148
**LPO**	0.000567564	-0.1482
**SPINT3**	0.005420935	-0.1485
**HLA-DQB2**	2.2404E-05	-0.148566667
**DNAJC5B**	0.001151335	-0.149
**TAS2R1**	0.005426022	-0.1509
**PATE2**	0.01485085	-0.1512
**ITGA11**	0.039359281	-0.1514
**PHOX2B**	0.000268404	-0.152033333
**CD300LG**	0.036954709	-0.152606667
**LCN10**	0.000714555	-0.153066667
**SYN3**	0.023658199	-0.153366667
**MORC1**	0.038821541	-0.154706667
**OR10J1**	0.036070395	-0.155066667
**C7orf34**	0.028439153	-0.156933333
**IL5**	0.036187939	-0.157133333
**LOC339166**	0.044541905	-0.157326667
**KIAA0125**	0.022173781	-0.1603
**SP7**	0.026974547	-0.161366667
**LRRC18**	0.035150056	-0.161966667
**KRTAP13-1**	0.021336614	-0.162
**PARVG**	0.041602325	-0.1624
**LOC283194**	0.014569537	-0.162866667
**TAS2R41**	0.020549742	-0.163233333
**OR6W1P**	0.018550635	-0.16426
**SPATA8**	0.004970017	-0.1666
**CXXC11**	0.047217323	-0.167133333
**OR8B8**	0.022862943	-0.167166667
**PLA2G5**	3.88731E-05	-0.167366667
**PSKH2**	0.002968979	-0.167666667
**RHO**	0.014779038	-0.168266667
**SPACA7**	0.000299578	-0.168866667
**LOC340357**	0.037824223	-0.169166667
**HTA**	0.002101283	-0.169533333
**CCL8**	0.022371678	-0.170766667
**STH**	0.048822744	-0.1717
**OR52D1**	0.046330402	-0.172533333
**CEACAM21**	0.012698008	-0.172933333
**T**	0.018556528	-0.173
**LOC100288122**	0.047961563	-0.1732
**LOC1720**	0.002073855	-0.173866667
**SCN10A**	0.035935272	-0.174133333
**BFSP2**	0.000358268	-0.174866667

**OR6B1**	**0.034684996**	**-0.175366667**
**PPY2**	0.000950534	-0.1759
**ATP6V0D2**	0.038145623	-0.175926667
**PLA2G2D**	0.016522381	-0.1769
**IL20**	0.016841845	-0.176993333
**BSN-AS2**	0.001033184	-0.1777
**RBMY2FP**	0.02680664	-0.178433333
**NUP210P1**	0.021844673	-0.178533333
**LY86**	0.002532258	-0.1795
**LINC00112**	0.018067153	-0.179733333
**CETP**	0.036394294	-0.18072
**LOC152578**	0.017394276	-0.180766667
**CHRM2**	0.010969325	-0.1808
**LOC285084**	0.030434632	-0.18106
**KLF17**	0.009244311	-0.18236
**BRS3**	0.001935198	-0.1836
**SLC17A1**	0.011970238	-0.184566667
**NCR2**	0.000233108	-0.185166667
**LOC339568**	0.003923167	-0.185266667
**WNT8A**	0.021837804	-0.185733333
**TTTY12**	0.014319312	-0.187333333
**LOC100505536**	0.021300612	-0.1896
**POM121L12**	0.030068992	-0.1899
**TBC1D26**	0.014466939	-0.1907
**IQCF3**	0.016857025	-0.191066667
**LOC284798**	0.025204005	-0.191266667
**LECT2**	0.017784674	-0.191433333
**ANKUB1**	0.002011985	-0.193266667
**OR12D3**	0.016018848	-0.194733333
**C8B**	0.009883353	-0.194966667
**KRTAP1-5**	0.049516323	-0.1973
**ODF4**	0.005739197	-0.197733333
**LOC645355**	0.00519062	-0.1998
**CCL13**	0.020415705	-0.201
**LOC283089**	0.046494447	-0.202273333
**WFDC8**	0.000173067	-0.202733333
**MRPS18B**	0.039139608	-0.205933333
**LST1**	0.006229961	-0.2063
**C22orf42**	0.017560525	-0.206333333
**SLA**	0.01935954	-0.209433333
**OR51E1**	0.000289181	-0.2115
**VWA7**	0.045713665	-0.213766667
**SLC26A8**	0.032694617	-0.21532
**LOC100128554**	0.000892817	-0.2157
**CCR8**	0.019330741	-0.2163
**OR5L2**	0.014434142	-0.216766667
**CIB4**	0.005766682	-0.216833333
**GK2**	0.020241241	-0.216866667
**CCR4**	0.000361672	-0.2169
**RTP3**	0.007029015	-0.2169
**HTR3B**	0.040613721	-0.219
**RGS8**	0.001479583	-0.2195
**FMO6P**	0.032784443	-0.2205
**SPATA19**	0.016947639	-0.2205
**OR52A1**	0.04224936	-0.2209
**NR1I3**	0.024679578	-0.221953333
**LOC154822**	0.02524649	-0.2245
**ADORA3**	0.008368875	-0.225166667
**APOL5**	0.012232139	-0.225166667
**MS4A5**	0.000658063	-0.225233333
**BPIFA4P**	0.013873871	-0.226466667
**OR8D2**	0.001301707	-0.2282
**GH2**	0.006892674	-0.2283

Table S2 (continued)

**ARHGEF15**	**0.028294426**	**-0.2307**
**LOC100271832**	0.005820091	-0.23656
**ERVV-1**	0.016496977	-0.236726667
**UBE2DNL**	0.022007122	-0.237166667
**OR8G1**	0.04739069	-0.2374
**KRT37**	0.016392471	-0.237466667
**LRRC52**	0.005800846	-0.239166667
**TREM1**	0.014224626	-0.240366667
**GPR52**	0.015928894	-0.241286667
**GPR52**	0.015928894	-0.241286667
**IL1F10**	0.013898065	-0.243013333
**LINC00330**	0.04352181	-0.2431
**INHBC**	0.032724458	-0.243793333
**PRKACG**	0.003338714	-0.244266667
**TAS1R2**	0.000414109	-0.244266667
**SLC8A3**	0.044432519	-0.2443
**LOC100507140**	0.008769323	-0.244466667
**STAP1**	0.014645763	-0.246146667
**KCNQ3**	0.009132613	-0.246253333
**TAS2R4**	0.024637274	-0.247006667
**LOC285000**	0.000223222	-0.2474
**LINC00310**	0.041906437	-0.24784
**TAAR8**	0.00641043	-0.2484
**TMCO2**	0.041065588	-0.248733333
**CCR3**	0.000285523	-0.2497
**OR2C1**	0.025824989	-0.252166667
**OR1C1**	0.005574681	-0.252733333
**ACTL7A**	0.03021005	-0.2528
**LINC00523**	0.010048776	-0.257066667
**SIGLEC1**	0.041847871	-0.2576
**KIF2B**	0.009784915	-0.259266667
**KIF2B**	0.009784915	-0.259266667
**MRGPRX2**	0.008432675	-0.259633333
**CSN2**	0.00669546	-0.2598
**ACTL9**	0.018111768	-0.262066667
**LOC100507605**	0.01489844	-0.265033333
**ANKRD26P3**	0.005346684	-0.26506
**LOC254559**	0.00999679	-0.270286667
**GIF**	0.000197699	-0.271166667
**PTGIR**	0.010171835	-0.2712
**OR2J3**	7.83185E-06	-0.271333333
**LOC100288079**	0.000487045	-0.271533333
**C7orf69**	0.019189058	-0.272066667
**NAV2-AS4**	2.87631E-05	-0.274133333
**COL11A2**	0.028878778	-0.274666667
**OR2L1P**	0.000827734	-0.275066667
**SYT6**	0.02153966	-0.275166667
**LOC100129427**	0.03802971	-0.276273333
**GPR61**	0.021342949	-0.277233333
**CD1B**	0.009304162	-0.2774
**C2orf53**	0.006862377	-0.2779
**CACNG6**	0.006979314	-0.27922
**CSN1S2AP**	0.010556799	-0.279733333
**LOC728084**	0.002882857	-0.282826667
**FLJ31485**	0.023942725	-0.283766667
**GPRC6A**	0.016273191	-0.288386667
**CEACAM3**	0.030289801	-0.290006667
**LILRA4**	0.042603344	-0.293633333
**MGC16025**	0.012424226	-0.298406667
**CXorf36**	0.001824651	-0.299866667
**GALR3**	0.01844829	-0.300033333
**SPATA16**	0.010470615	-0.30302
**KRTAP4-2**	0.025812873	-0.304166667

**SLCO1C1**	**0.031675359**	**-0.304193333**
**CIB3**	0.001187088	-0.3066
**OR10A4**	0.008992517	-0.306666667
**MGC4473**	0.001046091	-0.307566667
**ECEL1**	0.000124936	-0.309
**KNCN**	0.035580354	-0.3093
**CD1C**	0.008678506	-0.3104
**ASMT**	0.027372039	-0.311866667
**LHX3**	0.003336151	-0.315393333
**LOC100128946**	0.017797979	-0.316933333
**ERCC3**	0.044764777	-0.3205
**C20orf203**	0.011609895	-0.320726667
**FASTKD2**	0.02309532	-0.324593333
**KRT75**	0.021800455	-0.32774
**LOC730227**	0.010708419	-0.328086667
**RNASE3**	0.03765017	-0.330626667
**TDRG1**	0.002504538	-0.336233333
**FCGR2B**	0.000464144	-0.337433333
**MGC45922**	0.013967987	-0.33748
**MAS1**	0.037185731	-0.344766667
**NPBWR1**	0.01286871	-0.347333333
**RAB1A**	0.0484208	-0.351686667
**NPHP3**	0.026795575	-0.35224
**PRPF40A**	0.025018163	-0.354546667
**LCT**	0.027211227	-0.359946667
**GDF5**	1.25154E-06	-0.360766667
**KRTAP11-1**	0.002669295	-0.360766667
**AGXT2**	0.047385227	-0.3683
**OR1D2**	0.028951323	-0.371466667
**NLRP14**	0.000758899	-0.371793333
**MAPK1IP1L**	0.037817356	-0.388613333
**VPS33A**	0.027807827	-0.391626667
**NOL11**	0.003742765	-0.39774
**CSNK1G1**	0.026338141	-0.399173333
**USP34**	0.012329996	-0.400986667
**CAB39**	0.025032752	-0.4068
**PPP1R10**	0.002033604	-0.409766667
**PTPRT**	0.047830067	-0.4102
**POLR2D**	0.039856154	-0.411926667
**ESCO1**	0.041757544	-0.41452
**TMPRSS11D**	0.008214312	-0.4168
**LOC283761**	0.00370457	-0.417033333
**EIF2B2**	0.049262798	-0.418413333
**TAS2R14**	0.046945595	-0.41902
**RSPH6A**	0.045170576	-0.423833333
**ZFR**	0.039447899	-0.424093333
**TAS2R39**	0.039997199	-0.429533333
**SEC24B**	0.028185281	-0.430646667
**PUS1**	0.008858506	-0.433006667
**ZNF491**	0.020423082	-0.433693333
**DUSP21**	0.032560519	-0.4343
**PILRB**	0.035451401	-0.434346667
**PILRB**	0.035451401	-0.434346667
**WDR33**	0.047684732	-0.44502
**UBXN4**	0.012183843	-0.447066667
**SCYL3**	0.004897158	-0.44822
**BTF3P11**	0.001452032	-0.456233333
**BAG1**	0.028502093	-0.456253333
**ACTR3**	0.014434033	-0.461986667
**TAF9B**	0.041068107	-0.475473333
**BCL9L**	0.024595888	-0.48094
**PPIL3**	0.027499652	-0.489686667
**C1orf194**	0.001884812	-0.491533333

Table S2 (continued)

**WDR12**	**0.027702738**	**-0.491533333**
**ZNF713**	0.019084847	-0.493766667
**WDR75**	0.017422067	-0.49466
**TSN**	0.03049958	-0.499886667
**TCF7L2**	0.045308341	-0.50566
**SYF2**	0.037259245	-0.50732
**IWS1**	0.032002116	-0.519393333
**CCBP2**	0.034694113	-0.52266
**DIS3**	0.011578097	-0.52308
**MRPL30**	0.020020895	-0.52404
**OSBP**	0.014486337	-0.531726667
**GTF2B**	0.037710484	-0.53674
**CNNM3**	0.023472521	-0.540373333
**TPSAB1**	0.000884065	-0.5417
**CCDC91**	0.04777233	-0.54424
**SLC11A2**	0.021450676	-0.545733333
**ZNF84**	2.44771E-06	-0.559166667
**ZNF84**	2.44771E-06	-0.559166667
**CARD9**	0.03636763	-0.560506667
**ZNF622**	0.045989963	-0.5623
**PUS7L**	0.019221676	-0.565353333
**MC5R**	0.00140697	-0.566766667
**THUMPD1**	0.018688811	-0.56916
**ZC3H8**	0.047666661	-0.57654
**CCNT1**	0.049043428	-0.582586667
**PPP4R2**	0.030045364	-0.582953333
**LOC100129361**	0.027143908	-0.583906667
**SNHG8**	0.037205768	-0.584873333
**ZSCAN16**	0.017341334	-0.58918
**POC5**	0.028665198	-0.592773333
**LOC100132707**	0.036269263	-0.595073333
**MMGT1**	0.022253083	-0.602653333
**RANBP2**	0.01898273	-0.612193333
**ATAD1**	0.046462715	-0.616966667
**EDEM1**	0.001831672	-0.61722
**SEC24A**	0.030305355	-0.620393333
**MRPL35**	0.029764764	-0.62412
**TMEM167A**	0.008586123	-0.626726667
**ELF1**	0.033937402	-0.62918
**METTL15**	0.017334041	-0.630846667
**RPS27L**	0.049809998	-0.634313333
**EDF1**	0.026099353	-0.646026667
**SLC29A2**	0.005121484	-0.648433333
**SEC23IP**	0.034781818	-0.6506
**FRG1**	0.027743188	-0.651493333
**ARHGAP27**	0.000422504	-0.653506667
**ARHGAP27**	0.000422504	-0.653506667
**LIPT1**	0.047281679	-0.657066667
**CEP57**	0.001112445	-0.661273333
**VPS41**	0.010389755	-0.667306667
**PLEKHB2**	0.029410207	-0.68144
**CASP8**	0.016277623	-0.687246667
**PIP4K2C**	0.022831818	-0.69802
**NECAP1**	0.028043804	-0.700073333
**SEC16A**	0.018808009	-0.704306667
**NBR1**	0.040424129	-0.704893333
**STK40**	0.046506477	-0.707906667
**ZNF26**	0.025680495	-0.727666667
**ZNF26**	0.025680495	-0.727666667
**ZNF259**	0.01321673	-0.730153333
**CCT6A**	0.028089893	-0.741466667
**MED17**	0.016427772	-0.742206667
**TJP2**	0.016266341	-0.744973333

**SH3RF1**	**0.026908363**	**-0.74584**
**JRKL**	0.048005951	-0.7513
**RBM41**	0.014389156	-0.755193333
**CMTM8**	0.024832311	-0.758353333
**ATG4A**	0.033311367	-0.764406667
**ORC4**	0.021612304	-0.76898
**MAP7**	0.030683575	-0.775066667
**TNS3**	0.014515191	-0.795393333
**HCG4**	0.025061458	-0.8042
**FTSJD1**	0.007617171	-0.81598
**C15orf57**	0.003804502	-0.821633333
**EPB41L4A-AS1**	0.012493593	-0.82428
**ALKBH3**	0.018932428	-0.83042
**ARHGEF16**	0.045790511	-0.852753333
**ESRP2**	0.030881328	-0.852966667
**MTMR10**	0.04129175	-0.870446667
**TAF1D**	0.009071216	-0.876066667
**TAF1D**	0.009071216	-0.876066667
**PER2**	0.012038689	-0.892106667
**SLC22A5**	0.029162448	-0.894713333
**ZNF502**	0.030387747	-0.904306667
**MCL1**	0.030673642	-0.904886667
**C9orf142**	0.008186447	-0.910833333
**CDKN1A**	0.047250192	-0.911086667
**RSL1D1**	0.00248459	-0.926806667
**DDX52**	0.011433056	-0.927186667
**RNF135**	0.002474048	-0.928946667
**CDC42SE2**	0.044859079	-0.93866
**SNX19**	0.00376708	-0.95066
**PRRG4**	0.040505719	-0.951026667
**PSPH**	0.020004414	-0.968846667
**BCL2L1**	0.025974409	-0.973006667
**ANKRD49**	0.02109448	-0.973386667
**C1orf116**	0.021798021	-0.983593333
**ATXN7L3B**	0.049665788	-1.005733333
**SLC7A11**	0.011530387	-1.0094
**TMEM125**	0.045532069	-1.016193333
**TMEM159**	0.025955493	-1.01734
**ELMO3**	0.044101985	-1.024293333
**GJB2**	0.041991956	-1.026006667
**ST14**	0.029767647	-1.03286
**LOC728613**	0.002199123	-1.035286667
**SCRN3**	0.011134778	-1.040593333
**AP1M2**	0.000736479	-1.04922
**USP53**	0.004289373	-1.050766667
**PAK6**	0.029327877	-1.082466667
**FGFR2**	0.029359995	-1.103066667
**HSD17B12**	0.025657683	-1.10704
**SOWAHC**	0.021772073	-1.12298
**MDM2**	0.045251079	-1.133293333
**C12orf5**	0.043428222	-1.13706
**GPR160**	0.049205473	-1.1411
**PLEKHA7**	0.047478109	-1.150886667
**COL18A1**	0.01890599	-1.15204
**MSLN**	0.045921702	-1.152353333
**TBC1D30**	0.007495645	-1.16922
**HSBP1L1**	0.048098074	-1.175886667
**JUP**	0.031326801	-1.19096
**MXD1**	0.027906552	-1.193433333
**RAB17**	0.003222303	-1.200593333
**ITGB4**	0.018906599	-1.214993333
**BBS12**	0.006642683	-1.216413333
**NMI**	0.021442461	-1.229953333

Table S2 (continued)

**CREB5**	**0.005077111**	**-1.23918**
**LYPD6B**	0.045117232	-1.24106
**FGD4**	0.03883165	-1.280333333
**GRTP1**	0.009745964	-1.291713333
**LIPH**	0.040526119	-1.302786667
**PIP5K1B**	0.029147166	-1.329726667
**SLC44A3**	0.016390202	-1.33542
**C11orf54**	0.02265851	-1.34928
**F2RL1**	0.000138078	-1.35366
**LAMB3**	0.002992011	-1.361906667
**KLF5**	0.019048321	-1.386533333
**VAMP8**	0.001111162	-1.402386667
**CDKN2AIP**	0.038432456	-1.412653333
**C19orf33**	0.016669932	-1.436446667
**GPRC5A**	0.024845818	-1.453013333
**RAB3IP**	0.005099546	-1.458286667
**SLC16A14**	0.047259796	-1.463673333
**STARD10**	0.030548933	-1.470993333
**A2LD1**	0.001380124	-1.505373333
**EPCAM**	0.000260136	-1.531373333
**GALNT3**	0.003081572	-1.539853333
**CSRNP1**	0.047071882	-1.5435
**TMEM45B**	0.017246524	-1.584473333
**CGN**	0.001007878	-1.589
**ZNF140**	0.021046726	-1.5932
**USP43**	0.012812435	-1.63432
**GRHL2**	0.007615309	-1.643753333
**ELOVL7**	0.020446583	-1.65674
**MAL2**	0.011048869	-1.677426667
**GOLGA8A**	0.001337656	-1.68118
**C1orf210**	0.002947084	-1.7071
**SPINT1**	0.009308482	-1.71642
**TC2N**	0.010880611	-1.729
**ZNF605**	1.78727E-06	-1.742933333
**GDF15**	0.02634712	-1.788973333
**SGPP2**	0.002367352	-1.95228
**RAB25**	5.19134E-05	-1.994513333
**CAV2**	0.009874957	-2.003306667
**ESRP1**	0.004755092	-2.08226
**PPP1R1C**	0.029804345	-2.168873333
**S100P**	0.00185888	-2.292613333
**C3orf14**	0.000933695	-3.03554

Table S3

	Total cases				Non-diffuse carcinoma				Diffuse carcinoma			
	p-mTOR				p-mTOR				p-mTOR			
	Total	Low	High	P value	Total	Low	High	P value	Total	Low	High	P value
Age												
60 or low	280	170 (60.7)	110 (39.3)	0.345	133	80 (60.2)	53 (39.8)	0.586	142	87 (61.3)	55 (38.7)	0.778
over 60	330	194 (58.8)	136 (41.2)		244	139 (57.0)	105 (43.0)		85	54 (63.5)	31 (36.5)	
Gender												
Female	210	119 (56.7)	91 (43.3)	0.297	110	62 (56.4)	48 (43.6)	0.731	98	56 (57.1)	42 (42.9)	0.214
Male	400	245 (61.2)	155 (38.8)		267	157 (58.8)	110 (41.2)		129	85 (65.9)	44 (34.1)	
Lauren's classification												
Diffuse	227	141 (62.1)	86 (37.9)	0.347					227	141 (62.1)	86 (37.9)	
Non-diffuse	377	219 (58.1)	158 (41.9)		377	219 (58.1)	158 (41.9)					
Ming's classification												
Expanding	64	40 (62.5)	24 (37.5)	0.365	53	30 (56.6)	23 (43.4)	0.881	11	10 (90.9)	1 (9.1)	0.056
Infiltrative	546	324 (59.3)	222 (40.7)		324	189 (58.3)	135 (41.7)		216	131 (60.6)	85 (39.4)	
Tumor invasion												
EGC	187	117 (62.6)	70 (37.4)	0.190	127	76 (59.8)	51 (40.2)	0.659	59	40 (67.8)	19 (32.2)	0.350
AGC	423	247 (58.4)	176 (41.6)		250	143 (57.2)	107 (42.8)		168	101 (60.1)	67 (39.9)	
Lymphatic invasion												
Absent	281	174 (61.9)	107 (38.1)	0.168	162	94 (58.0)	68 (42.0)	1.000	118	80 (67.8)	38 (32.2)	0.076
Present	329	190 (57.8)	139 (42.2)		215	125 (58.1)	90 (41.9)		109	61 (56.0)	48 (44.0)	
Venous invasion												
Absent	508	295 (58.1)	213 (41.9)	0.045*	314	177 (56.4)	137 (43.6)	0.084	190	116 (61.1)	74 (38.9)	0.579
Present	102	69 (67.6)	33 (32.4)		63	42 (66.7)	21 (33.3)		37	25 (67.6)	12 (32.4)	
Perineural invasion												
Absent	384	243 (63.3)	141 (36.7)	0.011*	272	168 (61.8)	104 (38.2)	0.027*	108	72 (66.7)	36 (33.3)	0.218
Present	226	121 (53.5)	105 (46.5)		105	51 (48.6)	54 (51.4)		119	69 (58.0)	50 (42.0)	
pT stage												
pT1	187	117 (62.6)	70 (37.4)	0.094	127	76 (59.8)	51 (40.2)	0.211	59	40 (67.8)	19 (32.2)	0.405
pT2	113	65 (57.5)	48 (42.5)		84	47 (56.0)	37 (44.0)		29	18 (62.1)	11 (37.9)	
pT3	179	115 (64.2)	64 (35.8)		105	67 (63.8)	38 (36.2)		69	45 (65.2)	24 (34.8)	
pT4	131	67 (51.1)	64 (48.9)		61	29 (47.5)	32 (52.5)		70	38 (54.3)	32 (45.7)	
pN stage												
pN0	292	184 (63.0)	108 (37.0)	0.267	185	110 (59.5)	75 (40.5)	0.922	104	73 (70.2)	31 (29.8)	0.063
PN1	101	55 (54.5)	46 (45.5)		71	39 (54.9)	32 (45.1)		30	16 (53.3)	14 (46.7)	
pN2	96	59 (61.5)	37 (38.5)		56	33 (58.9)	23 (41.1)		39	25 (64.1)	14 (35.9)	
pN3	121	66 (54.5)	55 (45.5)		65	37 (56.9)	28 (43.1)		54	27 (50.0)	27 (50.0)	
pM stage												
pM1	567	341 (60.1)	226 (39.9)	0.242	357	209 (58.5)	148 (41.5)	0.490	204	128 (62.7)	76 (37.3)	0.651
pM2	43	23 (53.5)	20 (46.5)		20	10 (50.0)	10 (50.0)		23	13 (56.5)	10 (43.5)	
pTNM stage												
I	238	146 (61.3)	92 (38.7)	0.768	165	94 (57.0)	71 (43.0)	0.682	73	52 (71.2)	21 (28.8)	0.279
II	159	96 (60.4)	63 (39.6)		94	59 (62.8)	35 (37.2)		61	35 (57.4)	26 (42.6)	
III	170	99 (58.2)	71 (41.8)		98	56 (57.1)	42 (42.9)		70	41 (58.6)	29 (41.4)	
IV	43	23 (53.5)	20 (46.5)		20	10 (50.0)	10 (50.0)		23	13 (56.5)	10 (43.5)	

Table S4

Diffuse-type GC (227 cases)		Univariate analysis		Multivariate analysis	
Clinical parameters	Category	P value	HR (range)	P value	HR (range)
Gender	Male vs. Female	0.914	0.973 (0.594-1.594)		
Tumor invasion	Early GC vs. Advanced GC	0.001	38.068 (4.309-336.288)		
Lymphatic invasion	Absent vs. Present	0.000	6.176 (3.359-11.354)		
Venous invasion	Absent vs. Present	0.000	5.512 (3.297-9.215)		
Perineural invasion	Absent vs. Present	0.000	5.961 (3.111-11.420)		
pT class	pT1 vs. pT2,3,4	0.001	38.068 (4.309-336.288)		
pN class	pN0 vs. pN1,2,3	0.000	6.135 (2.713-13.674)		
pM class	pM0 vs. pM1	0.000	8.869 (5.174-15.202)		
Age	60 or lower vs. higher	0.017	1.808 (1.111-2.942)	0.004	2.111 (1.271-3.504)
pTNM stage	I vs II, III, V	0.000	13.397 (4.204-42.699)	0.000	35.585 (4.920-357.088)
p-mTOR	Low vs. High	0.044	1.676 (1.010-2.782)	0.159	1.439 (0.867-2.389)

Table S5

Total GC (610 cases)		Univariate analysis		Multivariate analysis	
Clinical parameters	Category	P value	HR (range)	P value	HR (range)
Gender	Male vs. Female	0.752	0.946 (0.670-1.336)		
Ming	Infiltrative vs. Expanding	0.036	0.444 (0.208-0.950)		
Tumor invasion	Early GC vs. Advanced GC	0.000	7.373 (3.987-13.635)		
Lymphatic invasion	Absent vs. Present	0.000	3.586 (2.452-5.245)		
Venous invasion	Absent vs. Present	0.000	3.076 (2.163-4.375)		
Perineural invasion	Absent vs. Present	0.000	3.750 (2.677-5.252)		
pT class	pT1 vs. pT2,3,4	0.000	7.373 (3.987-13.635)		
pN class	pN0 vs. pN1,2,3	0.000	5.883 (3.823-9.052)		
pM class	pM0 vs. pM1	0.000	8.723 (5.892-12.913)		
Age	60 or lower vs. higher	0.013	1.531 (1.095-2.140)	0.000	1.954 (1.350-2.829)
Lauren	Non-diffuse vs. Diffuse	0.022	1.470 (1.058-2.044)	0.015	1.558 (1.092-2.224)
pTNM stage	I vs II, III, V	0.000	6.275 (3.830-10.280)	0.000	6.545(3.758-11.399)
p-mTOR	Low vs. High	0.107	1.318 (0.942-1.844)	0.082	1.352(0.963-1.898)

Table S6

PDX	Gene	Protein variant	Coding	Mutation type	COSMIC ID	ClinVar	Variant frequency (%)	CNV
HGC-3	CDH1	p.Thr364fs	c.1089_1090delCA	Frameshift deletion	1379169		95.2 (256/269)	loss
	TP53	p.Glu11Gln	c.31G>C	Missense	11606		48.2 (593/1231)	
	ARID1A	p.Thr118Pro	c.352A>C	Missense	-		15.4 (70/454)	
		p.Ser317Gly	c.949A>G	Missense	-		33.6 (346/1031)	
		p.Ala349Val	c.1046C>T	Missense	-		15.6 (41/263)	
		p.Gln1579Pro	c.4736A>C	Missense	-		41.5 (227/547)	
		p.Asn2160fs	c.6473_6474insA	Frameshift deletion	1341432		49.6 (390/787)	
	CDKN2A							loss
	KRAS							gain
	PIK3CA							gain
HGC-18	KRAS	p.Gly12Asp	c.35G>A	Missense	521	pathogenic (#1)	46.1 (280/608)	
	MSH2	p.Glu569fs	c.1704_1705delAG	Frameshift Deletion	4972073		43.6 (95/218)	
	CTNNB1	p.Gly575Arg	c.1723G>A	Missense	4117552		49.2 (741/1505)	
	SMAD4	p.Arg361His	c.1082G>A	Missense	14122	pathogenic (#2)	100 (322/322)	loss
HGC-20	ARID1A	p.Ser1390Ile	c.4169G>T	Missense	-		46.8 (271/579)	
	PMS1	p.Arg277Gln	c.830G>A	Missense	442008		29.5 (33/112)	
	KRAS	p.Gly13Asp	c.38G>A	Missense	532	pathogenic (#3)	49.4 (245/496)	
	PIK3CA	p.Glu453del	c.1356_1358delAGA	Inframe deletion	5029127	pathogenic (#4)	46.0 (58/126)	
	SMAD4							loss
